# Imidazol-2-ylidene-Based NCCN Ligands for Chiral-at-Iron Catalysis

**DOI:** 10.1021/acs.organomet.6c00132

**Published:** 2026-06-05

**Authors:** Lukas Hinterlang, Sergei I. Ivlev, Eric Meggers

**Affiliations:** Fachbereich Chemie, Philipps-Universität Marburg, Hans-Meerwein-Strasse 4, 35043 Marburg, Germany

## Abstract

A new class of achiral tetradentate NCCN ligands containing two imidazol-2-ylidene moieties tethered by a tetraphenylene linker is reported. Upon coordination to iron­(II), these ligands enforce a *C*_2_-symmetric *cis*-α coordination geometry, where the helical twist of the tetradentate ligand induces metal-centered chirality. The racemic complexes were resolved into their individual enantiomers using a chiral auxiliary approach. Notably, the tetradentate framework provides significantly enhanced configurational stability compared to previously reported bis-bidentate analogues, showing negligible racemization in the presence of air and water. Finally, the catalytic utility of these chiral-at-iron complexes was demonstrated across three distinct asymmetric transformations, namely an intramolecular Cannizzaro reaction (up to 82% ee), an enantioselective C–H amidation (up to 81% ee), and a hetero-Diels–Alder reaction (up to 94% ee under open air conditions). Collectively, these results demonstrate the versatility of the new tetradentate NCCN architecture in stabilizing iron-based catalysts and facilitating efficient asymmetric induction through purely metal-centered chirality.

## Graphical abstract

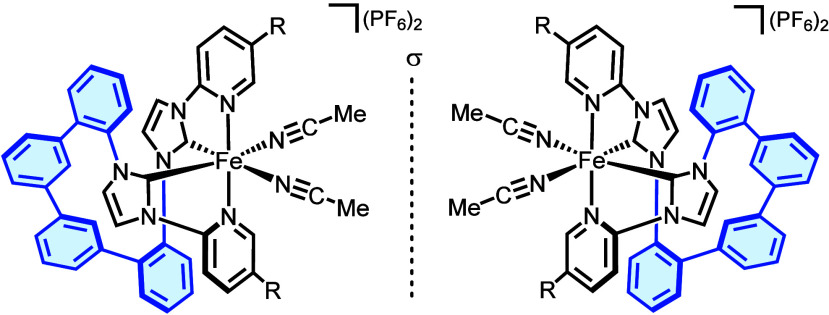

## Introduction

The inherent versatility of chiral transition metal complexes stems from a vast array of available metals, oxidation states, and coordination environments which renders them indispensable tools for asymmetric catalysis.^1^ Central to their success is ligand design, which dictates reactivity, modes of activation and the efficiency of asymmetric induction.^2^ Acyclic tetradentate ligands with four sequential donor atoms (often referred as linear tetradentate ligands), typically in combination with two ancillary monodentate ligands, have emerged as a popular choice for generating chiral octahedral catalysts.^3^ A key advantage of symmetrical linear tetradentate ligands is their ability to enforce *C*_2_ symmetry through *cis*-α coordination, in which the terminal donor atoms are arranged *trans* to each other, rather than through *cis*-β or *trans* coordination (Figure 1). This coordination mode effectively minimizes the number of diastereomeric transition states during catalysis. Furthermore, *cis*-α coordination induces a helical topology with Λ (left-handed helix) or Δ configuration (right-handed helix) which governs the stereochemical outcome of the catalyzed reaction.^3,4^

**Figure 1:**
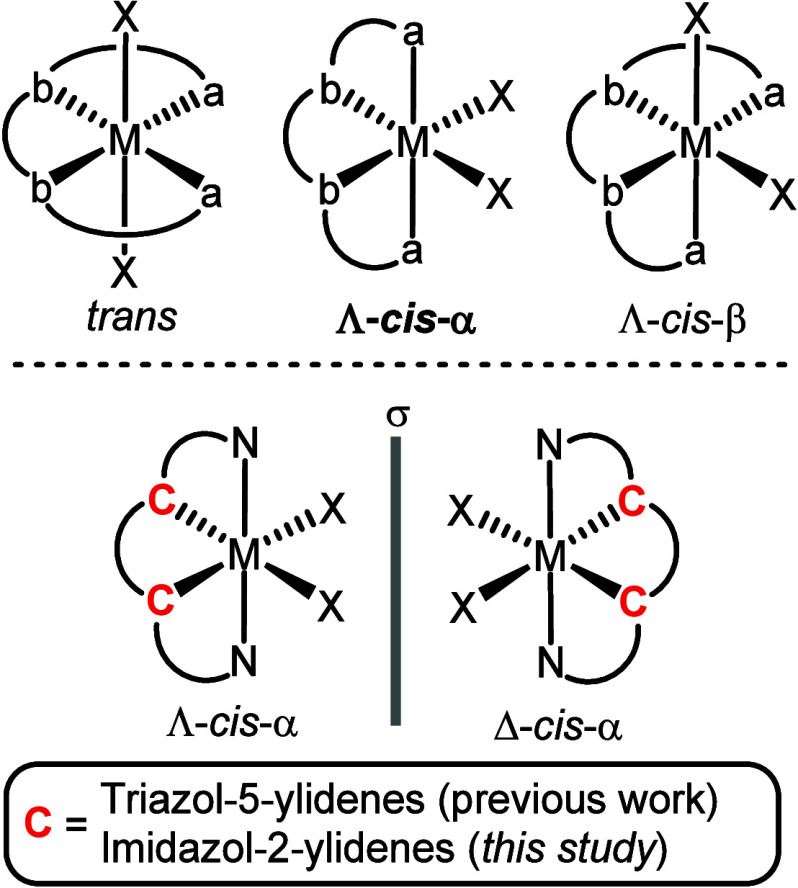
Octahedral metal complexes with symmetrical linear tetradentate ligands. Previous work and this study: NCCN linear tetradentate ligands with triazol-5-ylidenes or imidazol-2-ylidenes as C-donor ligands adopting a *cis*-α topology.

In the context of sustainable catalysis with iron, linear tetradentate ligands containing four nitrogen donor sites (N_4_ ligands), such as chiral bis­(iminomethyl)­diamines, have been extensively utilized because the chelate effect compensates for the kinetic lability of iron­(II) centers.^5^ To further modulate the electronic properties of multidentate ligands for iron, researchers have turned to N-heterocyclic carbenes (NHCs).^6–8^ Early work by Danopoulos^9^ and Hahn^10^ established the foundation for iron-NHC pincers and bidentate systems.^9^ Subsequent developments by Chen^11^ and Kühn^12^ explored tetradentate NCCN frameworks. However, these ligands typically resulted in square-planar geometries or, in the case of longer propylene linkers, sawhorse-type *cis*-β coordination (terminal donors *cis* to each other). While Glorius and co-workers recently utilized a CNNC ligand to achieve a distorted octahedral iron complex,^13^ a *C*_2_-symmetric *cis*-α NCCN iron catalyst has remained a synthetic challenge and we were recently the first to introduce such complexes based on mesoionic carbene ligands.^14^

In this work, we introduce a new class of linear NCCN ligands incorporating two imidazol-2-ylidene type NHC donor moieties. We demonstrate that these ligands coordinate to iron in a *cis*-α fashion to yield configurationally stable, *C*_2_-symmetric complexes. Significantly, even though the ligand framework itself is achiral, it induces metal-centered chirality, providing chiral-at-iron catalysts which are active in asymmetric transformations as demonstrated for a Cannizzaro reaction, a C–H amidation and a hetero-Diels–Alder reaction.

## Results and Discussion

### NCCN Ligand Design

We recently reported a novel class of chiral-at-iron catalysts wherein overall chirality originates solely from the stereogenic metal center, utilizing exclusively achiral ligands.^15–17^ In our initial design (**FeNHC**, Figure 2), the iron­(II) center is *cis*-coordinated by two bidentate *N*-(2-pyridyl)-substituted N-heterocyclic carbene (PyNHC) ligands and two labile acetonitrile molecules, with the dicationic complex balanced by hexafluorophosphate counterions. The absolute configuration (Λ or Δ) is determined by the helical twist of the PyNHC ligands. Crucially, while the PyNHC framework remains constitutionally and configurationally inert, the labile acetonitrile sites enable asymmetric transition metal catalysis without requiring chiral ligand precursors. Within the **FeNHC** architecture, the two mesityl substituents engage in interligand π-stacking, which positions two of their methyl groups in close proximity. Molecular modeling indicated that replacing these methyl groups with a biphenyl moiety would create an effective linker, yielding a new family of tetradentate ligands. To streamline the scaffold, the remaining mesityl methyl substituents were removed, resulting in a structure where two NHC moieties are tethered by a linker composed of four phenyl moieties. This design is analogous to our previous reports on an NCCN ligand containing 1,2,3-triazol-5-ylidene moieties.^14^

**Figure 2:**
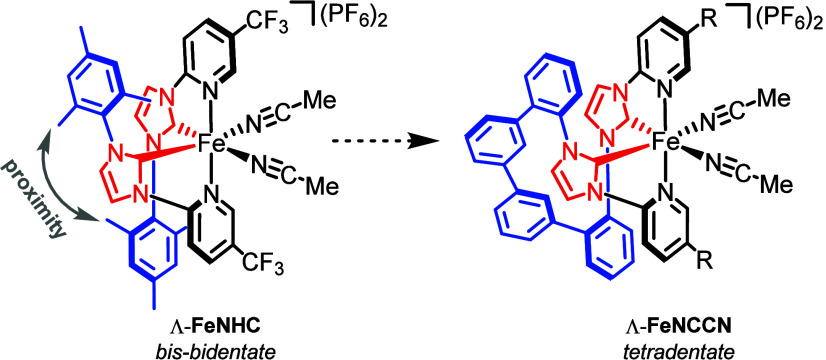
Linker design. Previous chiral-at-iron catalyst containing two pyridyl-substituted NHC ligands (**FeNHC**) (left) and corresponding iron complexes containing an NCCN ligand framework (**FeNCCN**) (right).

### Ligand Synthesis and Iron Coordination

Starting from commercially available 2-bromoaniline (**1**), the imidazole **2** was synthesized via a Debus-Radziszewski reaction using formaldehyde, glyoxal, and ammonium acetate (91%) (Scheme 1).^18^ The resulting bromo-phenyl-substituted imidazole **2** was then subjected to a double Suzuki cross-coupling with biphenyldiborane **3**, providing the coupling product **4** in 92% yield.^19^ The final ligands were then obtained via nucleophilic aromatic substitutions with the corresponding 2-bromo-5-pyridine derivatives **5**-**7**, and subsequent anion exchange furnished the hexafluorophosphate salts **8**–**10** in 73–94% yields.^20^

**Scheme 1:**
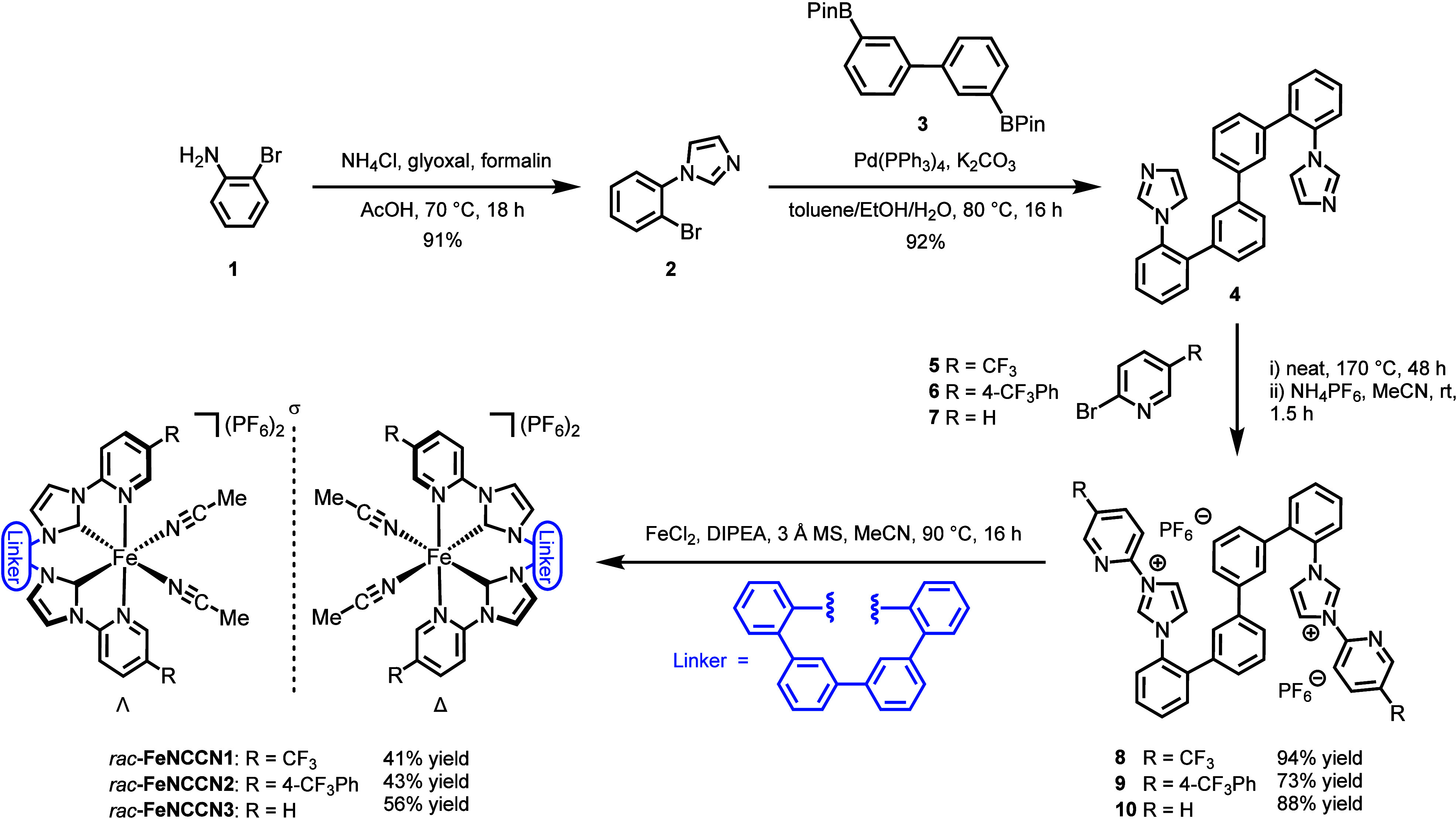
Ligand Synthesis and Coordination Chemistry to Generate Racemic FeNCCN Complexes^a^

a DIPEA = diisopropylethylamine.

The iron complexes were obtained by reacting the tetradentate bis-imidazolium salts with FeCl_2_ in the presence of the base diisopropylethylamine (DIPEA) thereby avoiding the use of expensive silver salts.^17^ Increasing the reaction temperature to 90 °C reduced the reaction time to 16 h and afforded the iron­(II) complexes *rac*-**FeNCCN1** and *rac*-**FeNCCN2** in isolated yields of 41 and 43%, respectively. A significantly higher yield of 56% was obtained for *rac*-**FeNCCN3**, which lacks a substituent on the pyridine ring.

The racemic complexes are air-stable and show no signs of decomposition when stored in acetonitrile under ambient conditions for several weeks (see Supporting Information for details). Importantly, even in the noncoordinating solvent dichloromethane, *rac*-**FeNCCN1** and *rac*-**FeNCCN3** remained intact for up to 1 week, while only *rac*-**FeNCCN2** began to decompose after 2 days. Nevertheless, these **FeNCCN** complexes are constitutionally more robust than related chiral-at-iron complexes with bis-bidentate ligands.

For structural elucidation, single crystals suitable for X-ray diffraction were obtained. The crystal structure of *rac*-**FeNCCN1** confirms the expected configurational motif, featuring a *cis*-α coordination geometry of the tetradentate bis-NHC ligand (Figure 3). As anticipated, the acetonitrile ligands occupy the equatorial plane together with the NHC moieties, while the pyridyl donors are positioned axially, resulting in a *C*_2_-symmetric *cis*-α topology. Compared to our previously reported complex bearing a tetradentate MIC-based ligand, the NHC-based *rac*-**FeNCCN1** exhibits a similar degree of distortion, most notably reflected in the twisting of the tetraphenylene linker.^14^

**Figure 3:**
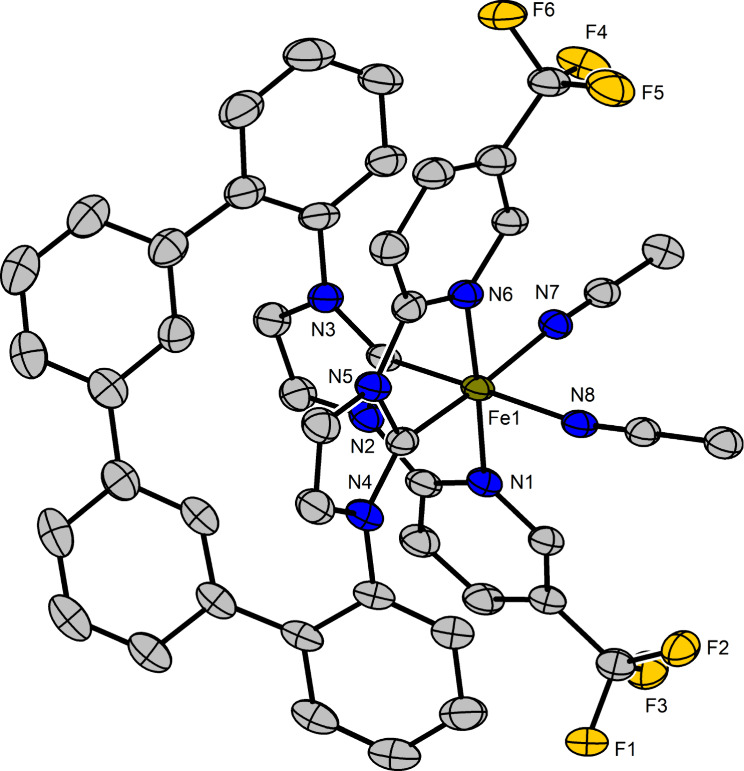
Crystal structure of the racemic iron complex *rac* **-FeNCCN1**. Counter ions and the hydrogen atoms are not shown. Displacement ellipsoids are shown at a 50% probability level at 100 K.

### Resolution of Enantiomers

The racemic complexes *rac*-**FeNCCN1**-**3** were resolved using our established chiral auxiliary method (Scheme 2).^21^ Accordingly, treatment of the racemic mixtures with the chiral salicyloxazoline derivative (*R*)-**Salox** under basic conditions afforded a mixture of diastereomers Λ- and Δ-(*R*)-**FeAux1**-**3**. Subsequent purification via silica gel chromatography provided the individual diastereomers Λ-(*R*)-**FeAux1**-**3** and Δ-(*R*)-**FeAux1**-**3** in isolated yields of 32–35% (64–70% of theory). In contrast to previously reported MIC-based NCCN analogues, both diastereomers were each formed in almost equal ratios under identical conditions. This is somewhat surprising, as the Λ-(*R*)-diastereomers exhibit intramolecular π–π stacking between the oxazoline phenyl substituent and a pyridyl ligand (see the X-ray structures of Λ- and Δ-(*R*)-**FeAux3** in Scheme 2), a feature that has been shown to enhance the stability of related auxiliary complexes.^14–17^ Thus, it can be concluded that the imidazolylidene framework possesses improved stability relative to its recently reported triazolylidene analogue, thereby minimizing the decomposition of the thermodynamically less stable diastereomers during chromatographic purification.^14^

**Scheme 2:**
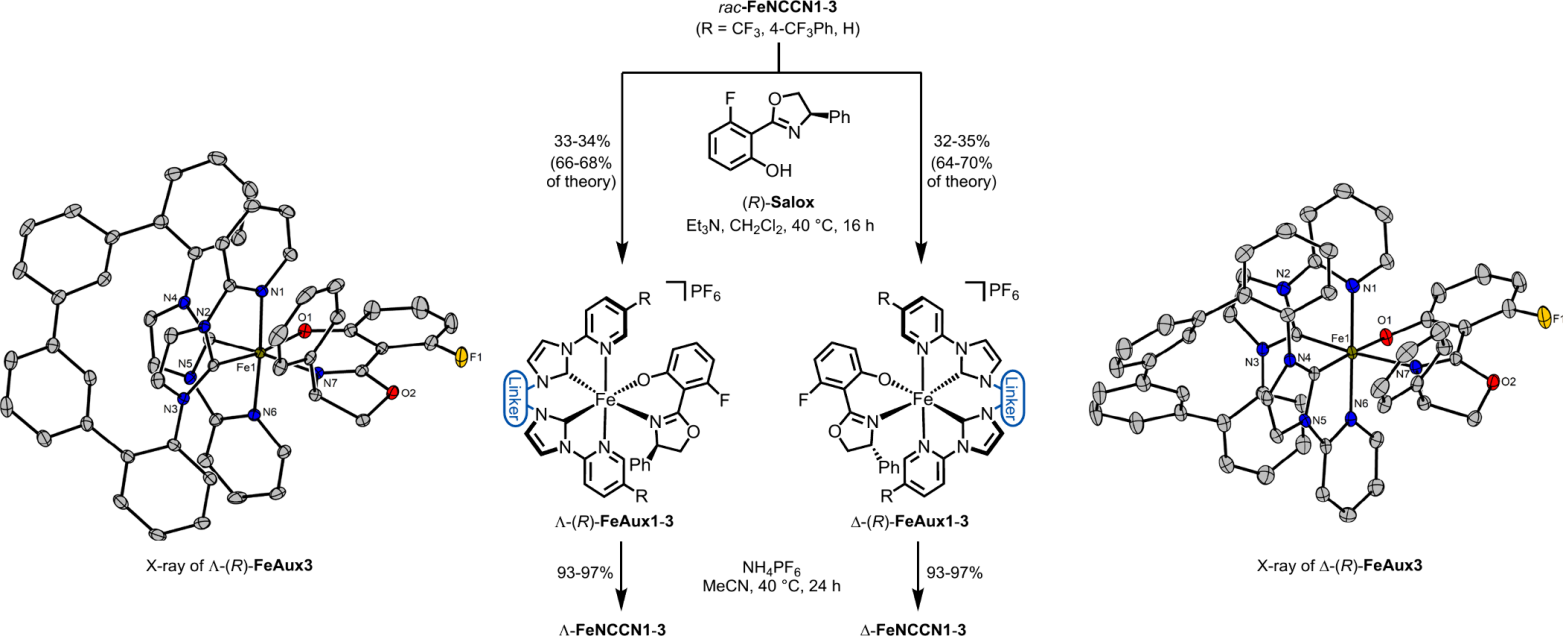
Chiral-Auxiliary-Mediated Synthesis of the Nonracemic Iron Complexes Λ- and Δ-**FeNCCN1**–**3** ^a^

a Crystal structures of Λ- and Δ-(*R*)-**FeAux3**: Counter ions and the hydrogen atoms are not shown. Displacement ellipsoids are shown at 50% probability level at 100 K.

It is worth noting that the synthetic protocol for the auxiliary complexes can be streamlined by reacting the crude racemic iron complexes *rac*-**FeNCCN1**-**3** directly with (*R*)-**Salox** after a simple filtration step (Scheme 3). This approach results in higher overall yields for the synthesis of Λ- and Δ-(*R*)-**FeAux1**-**2**, although not Λ- and Δ-(*R*)-**FeAux3**, and it circumvents a more time-consuming rigorous purification of the racemic iron precursor complexes and thus bypasses one chromatography step.

**Scheme 3:**
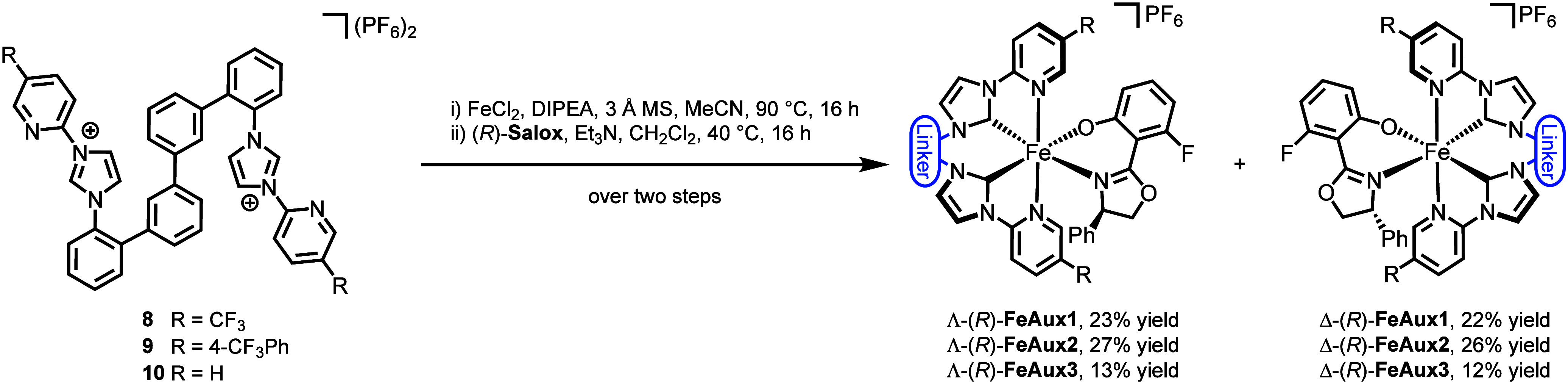
One-Pot Method for the Preparation of the Auxiliary Complexes without Isolation of the Racemic Complexes and Direct Addition of the Chiral Auxiliary^a^

a Shown are isolated yields based on the ligand. Note that theoretical yields are a factor of two higher.

The preparation of the enantiopure complexes was completed by the acidic cleavage of the auxiliary ligands, affording the individual enantiomers in excellent yields Λ- and Δ-**FeNCCN1** (93% each), Λ- and Δ-**FeNCCN2** (97% each), and Λ- and Δ-**FeNCCN3** (94 and 93%, respectively).^20^ The mirror-image relationship of these complexes was confirmed by circular dichroism (CD) spectroscopy (see Supporting Information).

### Configurational Stability of the Chiral-at-Iron Complexes

Next, the configurational stability of the newly developed chiral-at-iron complexes was evaluated by exposing enantiomerically pure samples to air and water (1% v/v) in a noncoordinating solvent (CD_2_Cl_2_). Racemization was assessed by reacting the mixture with a fluorine-containing chiral bidentate ligand, thereby converting the enantiomers into a set of diastereomers, which were subsequently analyzed by ^19^F NMR spectroscopy. This method has been previously established and discussed (see Supporting Information for details).^22^ As a result, starting from an initial ee of 97.2% for Δ-**FeNCCN1**, no evidence of racemization was observed after 24 or 48 h at room temperature (Table 1, entry 1). Similarly, Δ-**FeNCCN2** exhibited no detectable racemization under identical conditions (entry 2). In contrast, the complex Δ-**FeNCCN3**, which lacks a substituent at the 5-position of the pyridine ring, showed slight racemization of 0.5% after both 24 and 48 h (entry 3). This latter observation is consistent with the stability of related bis­(pyridyl)-NHC complexes, where the presence of electron-withdrawing CF_3_ groups on the pyridine moiety enhances the constitutional and configurational robustness of the system.^15^ However, overall the new tetradentate complexes Δ-**FeNCCN1**-**3** display markedly enhanced configurational stability compared to the bis-bidentate system. Under identical conditions, Δ-**FeNHC** underwent substantial racemization (entry 4), further highlighting the beneficial effect of the newly introduced tetraphenylene linker.^17^

**Table 1:** Configurational Stability of **FeNCCN** Complexes^a^

**entry**	**Fe-catalyst**	**initial ee (%)**	**ee after 24 h (%)**	**ee after 48 h (%)**
1	Δ-**FeNCCN1**	97.2	97.1	97.1
2	Δ-**FeNCCN2**	97.1	97.0	97.0
3	Δ-**FeNCCN3**	99.1	98.6	98.6
4	Δ-**FeNHC** ^17^	98.5	86.0	58.0

a Racemization study: The single enantiomers of the iron complexes were exposed to air for various lengths of time in CD_2_Cl_2_ with the addition of H_2_O (1% v/v). After this incubation time, a chiral F-containing ligand was coordinated and analyzed by ^19^F-NMR.

### Asymmetric Catalysis

Following the successful synthesis of the nonracemic complexes, their catalytic performance was evaluated. Initially, the new chiral-at-iron complexes were tested in the intramolecular Cannizzaro reaction of phenylglyoxal monohydrate (**11**) to mandelate ester (**12**).^23^ As summarized in Table 2, Δ-**FeNCCN2** and Δ-**FeNCCN3** afforded only moderate yields (73%) and enantioselectivities (45% ee) at room temperature (entries 3 and 5). Cooling the reaction to 4 °C for these catalysts resulted in a decreased yield (64%) with only marginal improvements in enantioselectivity (entries 4 and 6). The best results were obtained with Δ-**FeNCCN1** (R = CF_3_), which provided an 84% NMR yield and 78% ee at room temperature (entry 1). A similar trend was observed at 4 °C for this complex, where the ee increased to 82% at the expense of the yield (72%, entry 2).

**Table 2:**
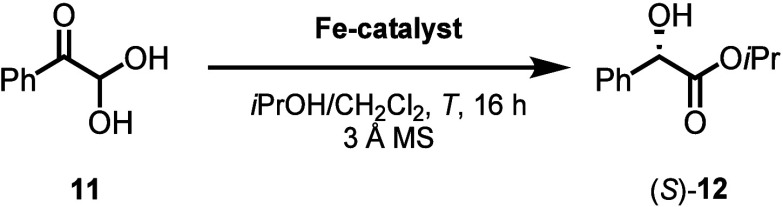
Intramolecular Cannizzaro Reaction^a^

entry	**Fe-catalyst**	***T***	**NMR-yield** ^b^ (%)	**ee** ^c^ (%)
1	Δ-**FeNCCN1**	rt	84	78
2	Δ-**FeNCCN1**	4 °C	72	82
3	Δ-**FeNCCN2**	rt	73	45
4	Δ-**FeNCCN2**	4 °C	64	45
5	Δ-**FeNCCN3**	rt	71	41
6	Δ-**FeNCCN3**	4 °C	64	44

a Reaction conditions: Substrate **11** (0.05 mmol), Δ-**FeNCCN1–3** (5 mol %), and 3 Å MS were placed in CH_2_Cl_2_ (0.05 M) under N_2_. Then *i*PrOH was added, and stirring was carried out for the indicated temperature.

b Determined by ^1^H NMR analysis of the crude product with 1,3,5-trimethoxybenzene as internal standard.

c Enantiomeric excess of the crude product determined by HPLC analysis on a chiral stationary phase.

The complexes Δ-**FeNCCN1**-**3** were subsequently evaluated in the nitrene-mediated ring-closing C­(sp^3^)–H amidation of benzoyloxyurea **13** into 2-imidazolidinone **14** (Table 3).^24^ Accordingly, at 4 °C, Δ-**FeNCCN1** provided 80% conversion (73% yield) with 71% ee (entry 1), while Δ-**FeNCCN2** and Δ-**FeNCCN3** achieved nearly quantitative conversion of the standard substrate (98 and 90% yield, respectively; entries 2 and 3). Notably, Δ-**FeNCCN3**, bearing an unsubstituted pyridyl moiety, exhibited no asymmetric induction (1% ee), highlighting the critical role of the pyridine substituent in providing enantioselectivity. Thus, **FeNCCN1** emerged as the catalyst of choice for this ring-closing amination. However, further lowering the temperature of the Δ-**FeNCCN1** system to −15 °C resulted in only a modest improvement in ee to 81%, while substantially decreasing both conversion and yield to 36% (entry 4). For Δ-**FeNCCN2**, the yield decreased to 88% at this temperature while the ee remained unchanged at 61% (entry 5). The lack of improvement in enantioselectivity prompted an investigation into substrate scope using the most efficient catalyst, Δ-**FeNCCN2**. Incorporating a bulky *t*Bu group into the leaving group yielded similar results (91% yield, 60% ee; entry 6), suggesting the leaving group plays a minor role in the enantiodetermining step. Consequently, we examined steric effects on the phenyl ring via methyl substitution. The *para*-substituted substrate showed a slight improvement in enantioselectivity (74% ee, 85% yield; entry 7), whereas the *meta*- and *ortho*-derivatives provided 59 and 66% ee, respectively (entries 8 and 9). Interestingly, the lower yield observed for the *para*-substituted substrate contrasts with our previously reported findings.^24c^

**Table 3:**
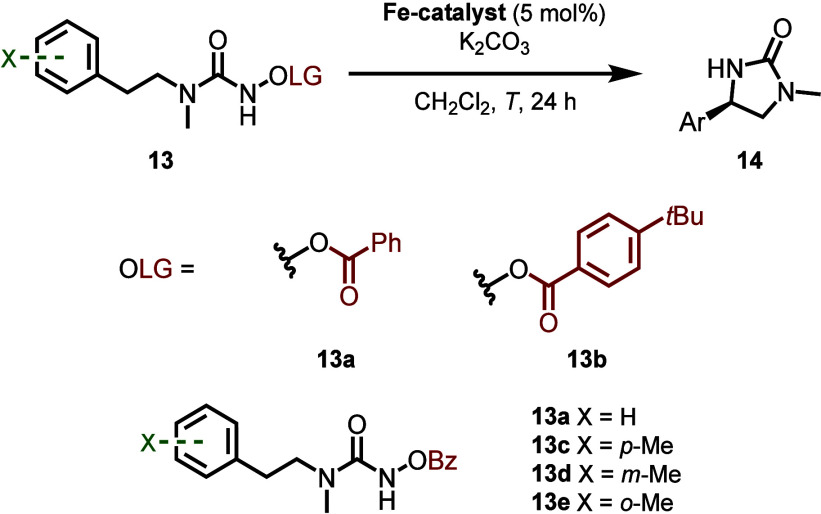
C­(sp^3^)–H-Amidation of Benzoyloxyurea Derivatives to 2-Imidazolidinones^a^

entry	**Fe-catalyst**	**substr.**	***T***	**conversion (%)**	**NMR-yield** ^b^ **(%)**	**ee (%)** ^c^
1	Δ-**FeNCCN1**	**13a**	4 °C	80	73	71 (*R*)
2	Δ-**FeNCCN2**	**13a**	4 °C	100	98	61 (*R*)
3	Δ-**FeNCCN3**	**13a**	4 °C	100	90	1 (*R*)
4	Δ-**FeNCCN1**	**13a**	–15 °C	36	36	81 (*R*)
5	Δ-**FeNCCN2**	**13a**	–15 °C	100	88	61 (*R*)
6	Δ-**FeNCCN2**	**13b**	4 °C	100	91	60 (*R*)
7	Δ-**FeNCCN2**	**13c**	4 °C	100	85	74 (*R*)
8	Δ-**FeNCCN2**	**13d**	4 °C	100	91	59 (*R*)
9	Δ-**FeNCCN2**	**13e**	4 °C	100	87	66 (*R*)
10	Λ-**FeNCCN1**	**13a**	4 °C	80	74	71 (*S*)

a Reaction conditions: Substrate **13a**–**e** (0.03 mmol), Δ-**FeNCCN1**-**3** (5 mol %), and K_2_CO_3_ (0.10 mmol) were placed in CH_2_Cl_2_ (0.1 M) under N_2_. Then, stirring was carried out at the indicated temperature.

b Determined by ^1^H NMR analysis of the crude product with 1,3,5-trimethoxybenzene as internal standard.

c Enantiomeric excess of the crude product determined by HPLC analysis on a chiral stationary phase.

While all of these catalytic reactions were carried out using iron catalysts with a metal-centered Δ-configuration, employing a corresponding Λ-configured iron complex instead afforded the expected enantiomeric product (compare entry 1 with entry 10).

To further evaluate their Lewis-acid catalytic activity, Δ-**FeNCCN1**-**3** were applied to the hetero-Diels–Alder (HDA) reaction of β,γ-unsaturated α-ketoester **15** with 2,3-dihydrofuran **16** to afford bicyclic dihydropyran **17** (Table 4).^16,17,25^ Under standard inert conditions, Δ-**FeNCCN1–3** provided high yields and enantioselectivities while maintaining excellent diastereocontrol. Modest differences were observed in enantioselectivity, with Δ-**FeNCCN1** yielding 86% ee (entry 1), compared to 94% ee for Δ-**FeNCCN2** (entry 2) and 92% ee for Δ-**FeNCCN3** (entry 3). To assess the robustness of the tetradentate NCCN-ligand architecture, the reactions were repeated under “open-flask” conditions in the presence of air and water (1% v/v). This resulted in only marginal decreases in yield (94–97%, entries 4–6), while both enantio- and diastereoselectivity remained virtually unchanged. These findings highlight the superior configurational stability of these NCCN-based complexes relative to their bis-bidentate analogs, demonstrating their utility for asymmetric catalysis in noninert environments.

**Table 4:**
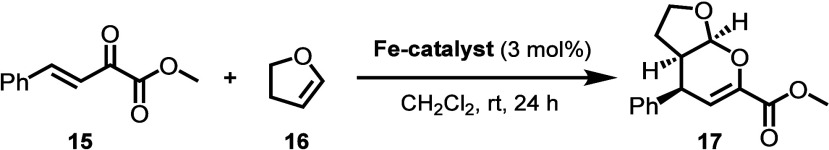
Inverse Electron Demand Hetero-Diels-Alder Reaction^a^

entry	**Fe-catalyst**	**conditions** ^a^	**NMR-yield** ^b^ (%)	**dr** ^c^	**ee** ^d^ (%)
1	Δ-**FeNCCN1**	N_2_ + dry	99	99:1	86
2	Δ-**FeNCCN2**	N_2_ + dry	98	99:1	94
3	Δ-**FeNCCN3**	N_2_ + dry	98	99:1	92
4	Δ-**FeNCCN1**	air + 0.1% H_2_O	97	99:1	85
5	Δ-**FeNCCN2**	air + 0.1% H_2_O	94	99:1	94
6	Δ-**FeNCCN3**	air + 0.1% H_2_O	94	99:1	93

a Reaction conditions: Ketoester **15** (0.05 mmol), Δ-**FeNCCN1–3** (3 mol %), and dihydrofuran **16** (0.80 mmol) were placed in distilled CH_2_Cl_2_ (0.05 M) under N_2_. Then, stirring was carried out at room temperature for 24 h.

b Determined by ^1^H NMR analysis of the crude product with 1,3,5-trimethoxybenzene as internal standard.

c Diastereomeric ratios were determined by ^1^H NMR.

d Enantiomeric excess of the crude product determined by HPLC analysis on a chiral stationary phase.

## Conclusions

In summary, we synthesized a series of achiral tetradentate NCCN ligands containing two imidazol-2-ylidene moieties that enforce *C*_2_-symmetric *cis*-α coordination to provide chiral-at-iron complexes. By utilizing a tetraphenylene linker, these systems achieve superior configurational stability over bis-bidentate analogues, resisting racemization even when exposed to air and moisture. The catalysts demonstrate promising performance across diverse asymmetric transformations, including an intramolecular Cannizzaro reaction (up to 82% ee), C­(sp^3^)–H amidation (up to 71% ee), and hetero-Diels–Alder (up to 94% ee). Notably, the high efficiency of the hetero-Diels–Alder reaction maintained under open-flask conditions underscores the practical utility of this catalyst scaffold. This work complements our recent work on NCCN ligands containing two 1,2,3-triazol-5-ylidenes and establishes the tetradentate NCCN scaffold as a powerful ligand toolbox for designing configurationally inert chiral-at-iron catalysts.^14^ Ongoing efforts in our laboratory are directed toward extending this ligand design to other earth-abundant metals.

## Experimental Section

### General Methods and Materials

All reactions were carried out under a nitrogen atmosphere in oven-dried glassware unless noted otherwise. Solvents for sensitive reactions were dried according to standard purification methods using calcium hydride (MeCN, CH_2_Cl_2_) and distilled under an atmosphere of nitrogen. The chemicals used are all from commercial sources and were used without further purification unless stated otherwise. For purification by column chromatography, Macherey-Nagel silica gel 60 M (irregularly shaped, 230–400 mesh, pH 6.8, pore volume: 0.81 mL/g, mean pore size: 66 Å, specific surface: 492 m^2^/g, particle size distribution: 0.5% < 25 μm and 1.7% > 71 μm, water content: 1.6%) was used as the stationary phase. ^1^H NMR, ^13^C­{^1^H} NMR and ^19^F­{^1^H} NMR spectra were recorded on a Bruker AV III HD 300 MHz, AV II 300 MHz, AV III HD 500 MHz, AV III 500 MHz, NEO 300 MHz, or NEO 600 MHz spectrometer at ambient temperature. All ^13^C and ^19^F NMR spectra were recorded as proton-decoupled spectra. The chemical shift δ is reported in parts per million (ppm) with the residual proton signal of the deuterated solvent as reference. ^19^F­{^1^H} NMR spectra were calibrated to trichlorofluoromethane (CFCl_3_, δ = 0 ppm) as external standard. All infrared spectroscopy was performed on a Bruker Alpha FT–IR spectrometer. Chiral HPLC was performed on an AGILENT 1200 or AGILENT 1260 HPLC system with DAICEL columns as chiral stationary phase with a size of 4.6 × 250 mm and a particle size of 5 μm. CD-spectra were recorded on a Jasco J-810 CD spectropolarimeter from 600–200 nm, 1 nm bandwidth, 2 s response time, 50 nm/min scanning speed, and an accumulation of 3 scans. High-resolution mass spectrometry was performed on a Finnigan LTQ-FT Ultra mass spectrometer (Thermo Fischer Scientific) using Electrospray ionization (ESI) as ionization source. Melting points (MPs) were determined on a Mettler Toledo MP70 using one end closed capillary tubes.

The following experimental section presents representative synthetic procedures for the ligand and the complex bearing a CF_3_-group at the 5-position of the pyridine ring. Syntheses and characterization of the related ligands and complexes are described within the Supporting Information.

### General Procedure for the Synthesis of the Tetradentate Ligands^20^

The bisimidazole **4** (1.00 equiv) (see Supporting Information for the synthesis of **4**) was mixed with the 5-substituted 2-bromopyridine **5**–**7** ^26^ (2.10 equiv) and placed in a Schlenk tube under an inert atmosphere. The tube was sealed and heated at 170 °C for 48 h in an oil bath. After cooling to room temperature, the residue was transferred with CH_2_Cl_2_ into a round-bottom flask, and the solvent was removed under reduced pressure to afford the bromide salt. The bromide salt was then dissolved together with NH_4_PF_6_ (5.00 equiv) in MeCN (0.16 M based on the imidazole) and stirred for 1.5 h under air. Afterward, the solvent was removed under reduced pressure, and the residue was taken up in CH_2_Cl_2_ to precipitate the ammonium salts and filtered again over Celite. The resulting filtrate of the crude hexafluorophosphate salts was dried under reduced pressure and purified by flash column chromatography (silica gel, CH_2_Cl_2_/MeOH, 98:2 → 90:10 → 80:20) to afford the corresponding bisimidazolium salts **8–10** as orange-brown solids.

#### Ligand **8**

Following the general procedure, the bisimidazolium salt **8** (1.80 g, 1.77 mmol, 94%) was obtained as a light brown solid starting from the precursor **4** (0.81 g, 1.86 mmol). ^1^H NMR: (500 MHz, CD_3_CN) δ (ppm) = 9.57 (t, *J* = 1.7 Hz, 1H), 8.91 (dt, *J* = 2.6, 0.9 Hz, 1H), 8.48–8.43 (m, 1H), 8.16–8.12 (m, 1H), 7.95–7.90 (m, 1H), 7.83–7.71 (m, 4H), 7.65–7.55 (m, 2H), 7.52 (dd, *J* = 2.2, 1.7 Hz, 1H), 7.44 (t, *J* = 7.7 Hz, 1H), 7.25 (ddd, *J* = 7.7, 1.8, 1.1 Hz, 1H). ^13^C NMR: (126 MHz, CD_3_CN) δ (ppm) = 149.5, 147.8, 147.7, 141.8, 139.3, 139.2, 138.3, 137.7, 136.2, 133.2, 132.8, 132.7, 132.7, 130.5, 130.5, 128.9, 128.6, 128.5, 128.3, 128.2, 127.5, 126.6, 120.4, 115.7. ^19^F-NMR: (282 MHz, CD_3_CN) δ (ppm) = −62.94 (s, 3F), −72.82 (d, ^1^ *J*_PF_ = 706.5 Hz, 6F). HRMS ESI­(+); *m*/*z* calculated for C_42_H_28_F_12_N_6_P [M–PF_6_]^+^: 875.19, found: 875.1910 [M–PF_6_]^+^. IR: ν̃ (cm^–1^) = 3159 (w), 1604 (w), 1542 (w), 1489 (w), 1412 (w), 1328 (m), 1261 (w), 1231 (w), 1173 (w), 1134 (m), 1078 (w), 1064 (w), 1018 (w), 970 (w), 956 (w), 821 (s), 760 (w), 740 (w), 708 (w), 677 (w), 657 (w), 614 (w), 555 (s), 514 (w), 490 (w), 468 (w), 427 (w).

### General Procedure for the Synthesis of Racemic Iron Complexes

Following a modified literature procedure,^17^ the bisimidazolium salts **8**, **9**, or **10** (1.00 equiv) (see Supporting Information for the synthesis of **8**–**10**), FeCl_2_ (1.00 equiv) and molecular sieve (3 Å, 1 g per 1.0 mmol ligand) were suspended in dry and degassed MeCN (0.025 M based on the imidazolium salt) under an atmosphere of nitrogen. After stirring for 5 min, DIPEA (2.50 equiv) was added and the mixture was heated to 90 °C for 16 h. The red-colored solution was allowed to cool to room temperature, diluted with MeCN, filtered over Celite, and the solvent was removed under reduced pressure. The residue was purified by flash column chromatography (silica gel, CH_2_Cl_2_/MeCN) with a NH_4_PF_6_ pad on top of the column to ensure complete elution of the desired complex. After removing the solvent under reduced pressure, the crude product was dissolved in CH_2_Cl_2_/MeCN (10:1) and washed three times with H_2_O to remove excess of PF_6_^–^-salts. The combined organic phases were then dried over MgSO_4_, filtered, and the solvent was removed under reduced pressure to afford the desired complexes *rac*-**FeNCCN1–3**.

#### *rac*-**FeNCCN1**

Following the general procedure, *rac*-**FeNCCN1** (188 mg, 0.16 mmol, 40%) was obtained as a red solid starting from ligand **8** (400 mg, 0.39 mmol). Purification condition: CH_2_Cl_2_/MeCN = 10:1 → 4:1. ^1^H NMR: (500 MHz, CD_2_Cl_2_) δ (ppm) = 8.88 (d, *J* = 2.0 Hz, 1H), 7.88 (dd, *J* = 8.7, 2.1 Hz, 1H), 7.70 7.65 (m, 2H), 7.59 (t, *J* = 7.7 Hz, 1H), 7.52 (dd, *J* = 7.7, 1.4 Hz, 1H), 7.40 (d, *J* = 1.4 Hz, 1H), 7.34–7.27 (m, 3H), 7.23 (dd, *J* = 7.8, 1.4 Hz, 1H), 6.29 (d, *J* = 2.3 Hz, 1H), 6.20 (d, *J* = 1.9 Hz, 1H), 2.58 (s, 3H). ^13^C NMR: (126 MHz, CD_2_Cl_2_) δ (ppm) = 196.9, 157.0, 151.8, 139.3, 137.7, 136.2, 134.7, 132.9, 130.6, 130.4, 129.8, 129.2, 128.8, 128.0, 126.8, 125.8, 118.2, 110.8, 4.5. ^19^F-NMR**:** (282 MHz, CD_2_Cl_2_) δ (ppm) = −62.18 (s, 3 F), −72.81 (d, ^1^ *J*_PF_ = 711.0 Hz, 6 F). HRMS: ESI­(+); *m*/*z* calculated for C_42_H_26_F_6_N_8_FeF [M–(PF_6_)_2_ – (MeCN)_2_ + F]^+^: 803.14, found: 803.1442 [M–(PF_6_)_2_ – (MeCN)_2_ + F]^+^. IR: ν̃ (cm^–1^) = 3149 (w), 2920 (w), 2851 (w), 1621 (w), 1508 (w), 1474 (w), 1425 (w), 1326 (m), 1306 (w), 1270 (w), 1253 (w), 1175 (w), 1135 (w), 1074 (m), 1042 (w), 947 (w), 824 (s), 772 (w), 763 (w), 748 (w), 705 (w), 680 (w), 665 (w), 620 (w), 600 (w), 555 (s), 508 (w), 469 (w), 435 (w).

### General Procedure for the Synthesis of Auxiliary Complexes^15^

The racemic iron complexes (1.00 equiv) and the chiral auxiliary (*R*)-**Salox** ^27^ (1.05 equiv) were dissolved in dry CH_2_Cl_2_ (0.04 M based on the iron complex) under an atmosphere of nitrogen. Et_3_N (1.50 equiv) was added, and the reaction mixture was stirred at 40 °C for 16 h. The solvent was then removed under reduced pressure, and the residue was purified by flash column chromatography (silica gel, CH_2_Cl_2_/MeCN, 100:1 → 75:1 → 50:1 → 25:1) to obtain both diastereomers of the auxiliary complexes.

Λ-(*R*)-**FeAux1** and Δ-(*R*)-**FeAux1**. Following the general procedure, Λ-(*R*)-**FeAux1** (25.4 mg, 21.4 μmol, 33%) was obtained as a purple solid from the corresponding complex *rac*-**FeNCCN1** (75.0 mg, 65 μmol). The other diastereomer Δ-(*R*)-**FeAux1** (26.4 mg, 22.3 μmol, 34%) was obtained as a purple solid.

#### Analytical Data for Λ-(*R*)-**FeAux1**

^1^H NMR: (600 MHz, CD_2_Cl_2_) δ (ppm)= 8.63 (s, 1H), 8.42 (s, 1H), 7.98 (d, *J* = 7.8 Hz, 1H), 7.71 (d, *J* = 8.6 Hz, 1H), 7.66 (d, *J* = 7.9 Hz, 1H), 7.62–7.50 (m, 5H), 7.41–7.20 (m, 10H), 7.18–7.08 (m, 3H), 6.93 (t, *J* = 7.5 Hz, 1H), 6.81 (s, 2H), 6.69 (d, *J* = 8.6 Hz, 1H), 6.37 (d, *J* = 8.5 Hz, 1H), 6.31 (d, *J* = 8.8 Hz, 3H), 6.27 (s, 1H), 6.21 (s, 1H), 6.15 (t, *J* = 10.1 Hz, 1H), 5.99 (s, 1H), 5.08–5.02 (m, 1H), 4.45 (dd, *J* = 9.4, 3.5 Hz, 1H). ^13^C NMR: (151 MHz, CD_2_Cl_2_) δ (ppm)= 159.1, 157.2, 151.7, 151.7, 151.4, 140.2, 139.9, 139.7, 137.3, 136.9, 136.7, 135.9, 135.8, 135.6, 135.0, 133.7, 131.5, 130.9, 130.8, 130.6, 130.5, 130.1, 129.9, 129.8, 129.5, 129.2, 129.0, 128.9, 128.9, 128.8, 127.6, 127.4, 126.6, 126.0, 126.0, 125.8, 124.1, 123.9, 123.9, 123.4, 123.1, 122.1, 122.0, 117.7, 117.4, 110.1, 109.1, 76.1, 73.8. ^19^F-NMR: (282 MHz, CD_2_Cl_2_) δ (ppm) = −62.31 (s, 3F), – 62.47 (s, 3F), −73.07 (d, ^1^ *J*_PF_ = 710.9 Hz, 6F), −106.07 (s, 1F). HRMS: ESI­(+); *m*/*z* calculated for C_57_H_37_F_7_FeN_7_O_2_ [M–(PF_6_)]^+^: 1040.22, found: 1040.2252 [M–(PF_6_)]^+^. IR: ν̃ (cm^–1^) = 3144 (w), 3085 (w), 1615 (m), 1577 (m), 1531 (w), 1505 (m), 1474 (w), 1447 (m), 1420 (w), 1387 (w), 1326 (s), 1312 (w), 1301 (w), 1256 (w), 1244 (w), 1233 (w), 1172 (w), 1133 (m), 1099 (w), 1072 (w), 1042 (w), 1004 (w), 991 (w), 954 (w), 944 (m), 924 (w), 832 (s), 795 (w), 771 (w), 762 (w), 747 (m), 702 (m), 677 (w), 661 (w), 620 (w), 600 (w), 582 (w), 557 (s), 534 (w), 510 (w), 462 (w).

#### Analytical Data for Δ-(*R*)-**FeAux1**

^1^H NMR: (500 MHz, CD_2_Cl_2_) δ (ppm) = 8.84 (s, 1H), 8.70 (s, 1H), 8.08 (d, *J* = 7.7 Hz, 1H), 7.90 (dd, *J* = 8.6, 2.1 Hz, 1H), 7.85 (d, *J* = 2.4 Hz, 1H), 7.68–7.59 (m, 3H), 7.59–7.51 (m, 5H), 7.52–7.44 (m, 2H), 7.37 (dtd, *J* = 7.6, 3.7, 1.4 Hz, 2H), 7.32–7.23 (m, 2H), 7.29–7.24 (m, 4H), 7.19–7.16 (m, 1H), 7.03 (td, *J* = 7.6, 1.2 Hz, 1H), 6.92 (dt, *J* = 18.0, 8.5 Hz, 2H), 6.41 (d, *J* = 1.9 Hz, 1H), 6.33 (td, *J* = 7.8, 1.6 Hz, 2H), 6.24 (d, *J* = 2.2 Hz, 1H), 6.18 (t, *J* = 9.6 Hz, 1H), 6.13 (d, *J* = 2.3 Hz, 1H), 5.97 (d, *J* = 1.9 Hz, 1H), 4.64 (dd, *J* = 9.3, 3.4 Hz, 1H), 4.46 (d, *J* = 7.7 Hz, 1H), 4.13 (t, *J* = 9.4 Hz, 1H), 3.46 (d, *J* = 9.4 Hz, 1H).

^13^C NMR: (126 MHz, CD_2_Cl_2_) δ (ppm) = 165.8, 163.2, 161.2, 158.7, 157.8, 153.4, 153.3, 151.5, 141.7, 139.9, 139.8, 137.5, 136.8, 136.1, 135.8, 135.7, 135.6, 135.6, 135.5, 135.1, 135.0, 133.4, 133.3, 131.4, 130.7, 130.6, 130.5, 130.3, 130.2, 130.0, 129.9, 129.5, 128.9, 128.8, 128.6, 128.5, 128.1, 128.0, 127.8, 126.2, 125.9, 125.5, 124.6, 124.3, 124.1, 123.2, 123.0, 121.9, 117.5, 117.5, 109.6, 109.0, 100.9, 100.7, 77.1, 69.1. ^19^F-NMR: (282 MHz, CD_2_Cl_2_) δ (ppm) = −61.97 (s, 3F), −62.48 (s, 3F), – 72.90 (d, ^1^ *J*_PF_ = 711.0 Hz, 6F), −110.35 (s, 1F). HRMS: ESI­(+); *m*/*z* calculated for C_57_H_37_F_7_FeN_7_O_2_ [M – (PF_6_)]^+^: 1040.22, found: 1040.2217 [M–(PF_6_)]^+^. IR: ν̃ (cm^–1^) = 3144 (w), 3077 (w), 1619 (m), 1588 (w), 1536 (w), 1504 (m), 1475 (w), 1447 (m), 1419 (m), 1386 (w), 1356 (w), 1326 (s), 1311 (w), 1283 (w), 1262 (w), 1233 (m), 1171 (w), 1136 (m), 1099 (w), 1085 (w), 1072 (w), 1038 (m), 1006 (w), 992 (w), 944 (m), 913 (w), 838 (s), 796 (w), 772 (w), 765 (m), 704 (m), 676 (w), 663 (w), 621 (w), 599 (w), 586 (w), 557 (s), 533 (w), 512 (w), 471 (w).

### General Procedure for the Auxiliary Cleavage

Following a modified procedure from the literature.^15^ The single diastereomers of the auxiliary complexes (1.00 equiv) were dissolved in dry MeCN (0.02 M based on the auxiliary complex), and an excess of NH_4_PF_6_ (10.00 equiv) was added, and the mixture was stirred at 40 °C for 16 h. Afterward, the solvent was removed under reduced pressure and Et_2_O was added. The precipitated solids were transferred to a Celite pad and washed several times with Et_2_O to remove any residual free auxiliary. The complex was then eluted with CH_2_Cl_2_/MeCN, 30:1 to afford the corresponding Λ- or Δ-iron complexes after the solvent had been removed under reduced pressure.

#### Λ-**FeNCCN1**

Following the general procedure, Λ-**FeNCCN1** (55 mg, 47.6 μmol, 94%) was obtained as a red solid from the corresponding complex Λ-(*R*)-**FeAux1** (60 mg, 51.0 μmol). The spectroscopic data of the enantiopure complex were in accordance with *rac*-**FeNCCN1**. **CD** (MeCN, 0.25 mM): Δ, nm (Δε, M^–1^ cm^–1^) 234 (+98), 259 (−99), 282 (+60), 302 (−13), 330 (+4), 355 (+2), 390 (+16), 442 (−23), 517 (+4).

#### Δ-**FeNCCN1**

Following the general procedure, Δ-**FeNCCN1** (66 mg, 57.1 μmol, 94%) was obtained as a red solid from the corresponding complex Δ-(*R*)-**FeAux1** (72 mg, 61.0 μmol). The spectroscopic data of the enantiopure complex were in accordance with *rac*-**FeNCCN1**. **CD** (MeCN, 0.25 mM): Λ, nm (Δε, M^–1^ cm^–1^) 234 (−106), 259 (+119), 282 (−71), 302 (+16), 330 (−5), 355 (−3), 390 (−16), 442 (+25), 517 (−4).

### General Procedure for the Intramolecular Cannizzaro Reaction

A modified literature procedure was followed.^15^ The iron catalyst (5 mol %), molecular sieve (3 Å, 5 mg per 0.01 mmol substrate) and phenylglyoxal (**11**) (7.6 mg, 0.05 mmol, 1.00 equiv) were suspended in dry and degassed CH_2_Cl_2_ (0.05 M) under an atmosphere of nitrogen in a Schlenk tube. Then, *i*PrOH (37.5 μL, 0.50 mmol, 10.0 equiv) was added and the mixture was stirred for 16 h at the indicated temperature. Subsequently, *n*-pentane (5 mL) was added, and the reaction mixture was filtered over a short Celite plug to remove the catalyst and rinsed with *n*-pentane/CH_2_Cl_2_ (4:1). The solvent was removed under reduced pressure and the yield was determined by ^1^H NMR analysis of the crude product with TMB as internal standard. The enantiomeric excess of the crude product was determined by HPLC analysis on a chiral stationary phase. The spectroscopic data are in accordance with the literature.^15^ The absolute configuration of the product was determined by comparison of the HPLC traces with those reported in the literature.^15^ TLC: *R*_f_ = 0.41 (*n*-pentane/EtOAc 3:1). ^1^H NMR: (300 MHz, CDCl_3_) δ (ppm) = 7.44–7.30 (m, 5H), 5.14–5.01 (m, 2H), 3.47 (d, *J* = 5.8 Hz, 1H), 1.28 (d, *J* = 6.3 Hz, 3H), 1.11 (d, *J* = 6.2 Hz, 3H) HPLC: Daicel Chiralcel OD-H column, 250 × 4.6 mm, absorbance at 210 nm, *n*-hexane/*i*PrOH 95:5, isocratic flow, flow rate 1.0 mL/min, 25 °C, *t*_r_ (minor) = 7.67 min, *t*_r_ (major) = 14.72 min.

### General Procedure for the C­(sp^3^)–H-Amidation of Benzoyloxyurea Derivatives

Following a slightly modified procedure from the literature.^24d^ The iron catalyst (5 mol %), K_2_CO_3_ (0.10 mmol, 3.00 equiv) and the urea substrate **13a**–**e** ^24c^ (0.03 mmol, 1.00 equiv) were placed in a Schlenk tube under an atmosphere of nitrogen. Dry and degassed CH_2_Cl_2_ (0.1 M) was added, and the reaction mixture was stirred for 24 h at the indicated temperature. According to that, *n*-pentane (5 mL) was added, and the reaction mixture was filtered over a short Celite plug to remove the catalyst and rinsed with *n*-pentane/CH_2_Cl_2_ (4:1). The solvent was removed under reduced pressure and the yield was determined by ^1^H NMR analysis of the crude product with TMB as internal standard. The enantiomeric excess of the crude product was determined by HPLC analysis on a chiral stationary phase. The spectroscopic data are in accordance with the literature.^24c^ The absolute configuration of the product was determined by comparison of the HPLC traces with those reported in the literature.^24a^

**14a**. TLC: *R*_f_ = 0.17 (*n*-pentane/EtOAc 2:1). ^1^H NMR: (300 MHz, CDCl_3_) δ (ppm) = 7.38–7.28 (m, 5H), 5.41 (s, 1H), 4.76 (t, *J* = 8.1 Hz, 1H), 3.79 (t, 1H), 3.24 (t, *J* = 8.0 Hz, 1H), 2.83 (s, 3H). HPLC: Daicel Chiralcel IA column, 250 × 4.6 mm, absorbance at 220 nm, *n*-hexane/*i*PrOH 95:5, isocratic flow, flow rate 1.0 mL/min, 30 °C, *t*_r_ (minor) = 28.60 min, *t*_r_ (major) = 22.86 min.

### General Procedure for the Hetero-Diels–Alder Reaction

Following a slightly modified procedure from the literature.^16^ The iron catalyst (3 mol %) and ketoester **15** ^28^ (0.05 mmol, 1.00 equiv) were dissolved in dry and degassed CH_2_Cl_2_ (0.05 M) under an atmosphere of nitrogen in a Schlenk tube. Then, dihydrofuran **16** (0.80 mmol, 15.0 equiv) was added, and the mixture was stirred at room temperature for 24 h. According to that, *n*-pentane (5 mL) was added, and the reaction mixture was filtered over a short Celite plug to remove the catalyst and rinsed with *n*-pentane/CH_2_Cl_2_ (4:1). The solvent was removed under reduced pressure and the yield was determined by ^1^H NMR analysis of the crude product with TMB as internal standard. The enantiomeric excess of the crude product was determined by HPLC analysis on a chiral stationary phase. The spectroscopic data are in accordance with the literature.^16^ The absolute configuration of the product was determined by comparison of the HPLC traces with those reported in the literature.^16^ TLC: *R*_f_ = 0.32 (*n*-pentane/EtOAc 5:1). ^1^H NMR: (300 MHz, CDCl_3_) δ (ppm) = 7.37–7.20 (m, 5H), 6.20 (dd, *J* = 1.3 Hz, 1H), 5.64 (d, *J* = 3.5 Hz, 1H), 4.20–4.13 (m, 2H), 3.90–3.86 (m, 1H), 3.83 (s, 3H), 2.71–2.59 (m, 1H), 1.71 (tt, *J* = 12.3 Hz, 1H), 1.37–1.31 (m, 1H). HPLC: Daicel Chiralcel IG column, 250 × 4.6 mm, absorbance at 254 nm, *n*-hexane/iPrOH 95:5, isocratic flow, flow rate 1.0 mL/min, 25 °C, *t*_r_ (minor) = 32.25 min, *t*_r_ (major) = 38.36 min.

## Supplementary Material



## Notes

Detailed experimental procedures, analytical data, NMR spectra, CD spectra, HPLC traces, and crystallographic data (PDF)
