# Supplementary material for: Imidazol-2-ylidene-Based NCCN Ligands for Chiral-at-Iron Catalysis
Source: Organometallics. 2026 Jun 5;45(12):1428–36. doi: 10.1021/acs.organomet.6c00132 (PMC13292349; doi:10.1021/acs.organomet.6c00132)
Supplement: Supplementary file 1 [file om6c00132_si_001.pdf]

Supporting Information

Imidazol-2-ylidene-Based NCCN Ligands for Chiral-at-Iron Catalysis

Lukas Hinterlang, Sergei I. Ivlev, and Eric Meggers*

Fachbereich Chemie, Philipps-Universität Marburg, Hans-Meerwein-Strasse 4, 35043 Marburg, Germany

*Email: meggers@chemie.uni-marburg.de

Table of Contents

1. General Information S1

2. Ligand Synthesis S1

3. Synthesis of Racemic Iron Complexes S7

4. Synthesis of Auxiliary Complexes S11

5. Cleavage of the Chiral Auxiliary S19

6. Determination of Enantiomeric Excess S23

7. Stability Experiments S25

8. Catalysis S31

9. Synthesis of the Chiral Auxiliaries and Pyridine 6 ..... S34

10. NMR Spectra S37

10. Chiral HPLC Traces S58

11. CD-Spectra S62

12. Single Crystal X-Ray Diffraction S63

13. References S73

1. General Information

The description of all working techniques can be obtained from the general information paragraph of the main manuscript. The chemicals used are all from commercial sources and were used without further purification unless stated otherwise. Substrates **13a-e**[1] and **15**[2] were synthesized after a modified literature procedure.

2. Ligand Synthesis

1-(2-bromophenyl)-1*H*-imidazole (2)

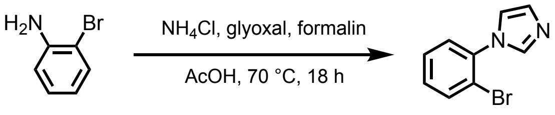

1 **2**

Following a modified procedure from the literature.[3] Formalin (0.98 mL, 13.1 mmol, 1.50 eq, 37% in H_2O) and glyoxal (1.50 mL, 13.1 mmol, 1.50 eq, 40% in H_2O) were dissolved in 2 mL of glacial acetic acid (0.22 mL/mmol aniline) and heated to 70 °C. A solution of bromoaniline **1** (1.5 g, 8.72 mmol, 1.00 eq) and NH_4OAc (1.00 g, 13.1 mmol, 1.50 eq) in 2 mL AcOH and 0.40 mL H_2O was added dropwise. The reaction mixture was stirred at 70 °C for 18 hours and, after cooling to room temperature, was carefully added to a stirred solution of 5.88 g $NaHCO_3$ in 60 mL H_2O. $NaHCO_3$ was then further added in small portions until a pH of 7–8 was reached. The precipitated solids were filtered off, and the filtrate was extracted three times with EtOAc. The combined organic phases were washed with brine, dried over $MgSO_4$ and the solvent was then removed under reduced pressure. The crude product was purified by flash column chromatography (silica gel, *n*-hexane/EtOAc, 90:10 → 10:90) to obtain 1-(2-bromophenyl)-imidazole (**2**, 1.75 g, 7.88 mmol, 90%) as a yellow solid. The spectroscopic data are in accordance with those reported in the literature.[4]

TLC: R_f = 0.30 (*n*-pentane/EtOAc 10:1).

^{1}H-NMR (300 MHz, $CDCl_3$) δ (ppm) = 7.72 (dd, *J* = 8.2, 1.4 Hz, 1H), 7.66 (t, *J* = 1.1 Hz, 1H), 7.42 (ddd, *J* = 8.0, 6.9, 1.5 Hz, 1H), 7.34–7.23 (m, 2H), 7.19 (t, *J* = 1.2 Hz, 1H), 7.12 (t, *J* = 1.3 Hz, 1H).

Biphenyl-linked imidazole 4

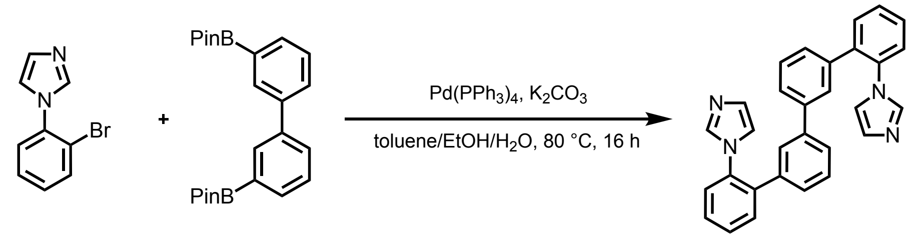

2 **3** **4**

Following a modified procedure from the literature.[5] The arylboronic ester **3** (0.10 g, 0.24 mmol, 1.00 eq), K_2CO_3 (0.13 g, 0.98 mmol, 4.00 eq), $Pd(PPh_3)_4$ (14.2 mg, 0.01 mmol, 0.05 eq), and the imidazole **2** (0.11 g, 0.51 mmol, 2.10 eq) were placed under nitrogen atmosphere in a SCHLENK tube. Toluene/EtOH/H_2O (3:1:1, 0.05 M based on the boronic ester) was added, and the reaction mixture was stirred at 80 °C for 16 h. After cooling to room temperature, the mixture was diluted with H_2O and extracted three times with EtOAc. The combined organic phases were then washed with brine and dried over $MgSO_4$. The solvent was removed under reduced pressure, and the crude product was purified by flash column chromatography (silica gel, CH_2Cl_2/MeOH, 99:1 → 90:10) to obtain the biphenyl-linked imidazole **4** (99.4 mg, 0.22 mmol, 92%) as a colorless solid.

TLC: R_f = 0.10 (CH_2Cl_2/MeOH 25:1).

MP: 167 °C.

^{1}H-NMR: (600 MHz, CD_2Cl_2) δ (ppm) = 7.61–7.48 (m, 3H), 7.45–7.35 (m, 4H), 7.20 (dt, J = 2.0, 1.0 Hz, 1H), 7.12 (dt, J = 7.2, 1.6 Hz, 1H), 6.98 (t, J = 1.1 Hz, 1H), 6.93 (t, J = 1.3 Hz, 1H).

^{13}C-NMR: (151 MHz, CD_2Cl_2) δ (ppm) = 141.4, 138.9, 137.9, 137.7, 135.7, 131.7, 129.5, 129.4, 129.1, 129.1, 127.8, 127.6, 126.9, 126.8, 121.1.

HRMS: ESI(+); m/z calculated for $C_{30}H_{22}N_4H$ $[M+H]^+$: 439.18, found: 439.1816 $[M+H]^+$.

IR: $\tilde{\nu}$ (cm^{-1}) = 3108 (w), 3055 (w), 1597 (w), 1580 (w), 1502 (s), 1469 (w), 1422 (w), 1388 (w), 1360 (w), 1297 (m), 1237 (w), 1126 (w), 1107 (w), 1058 (m), 1012 (w), 963 (w), 902 (m), 814 (w), 795 (w), 761 (s), 744 (w), 705 (s), 676 (w), 660 (s), 613 (m), 523 (m), 489 (w), 472 (w), 430 (w).

Note: On a 700 mg scale, only a reduced yield of 79% could be achieved, which can be partly attributed to the monocoupled side product that is difficult to separate from the main product.

General Procedure A: Bis-imidazolium salts 8-10

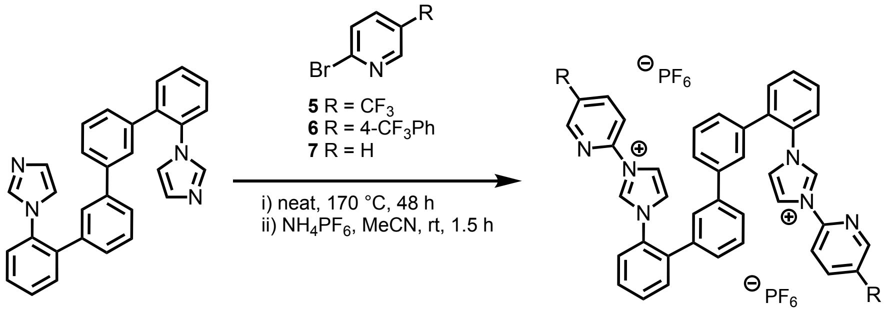

4 8-10

Following a modified procedure from the literature.[6] The biphenyl-linked imidazole **4** (1.00 eq) was mixed with the 5-substituted 2-bromopyridine **5-7** (2.10 eq) and placed in a Schlenk tube under an inert atmosphere. The tube was sealed and heated at 170 °C for 48 hours in an oil bath. After cooling to room temperature, the residue was transferred into a round-bottom flask with CH_2Cl_2, and the solvent was removed under reduced pressure to afford the bromide salt. The bromide salt was then dissolved together with NH_4PF_6 (5.00 eq) in MeCN (0.16 M based on the imidazole) and stirred for 1.5 h under air. Afterward, the solvent was removed under reduced pressure, and the residue was taken up in CH_2Cl_2 to precipitate the ammonium salts and filtered again over Celite. The resulting filtrate of the crude hexafluorophosphate salts was dried under reduced pressure and purified by flash column chromatography (silica gel, CH_2Cl_2/MeOH, 98:2 → 90:10 → 80:20) to afford the corresponding ligands **8-10** as orange-brown solids.

Note: The Schlenk tube should be immersed in the oil bath as much as possible to minimize the sublimation of the volatile pyridines **5** and **7** at the top (See Figure S1 for the reaction setup). To ensure a smooth reaction of the solids, it is best to place the Schlenk tube in the oil bath at room temperature, then slowly heat it to the intended reaction temperature of 170 °C (Typically, the reaction mixture solidifies after 18 h at this temperature).

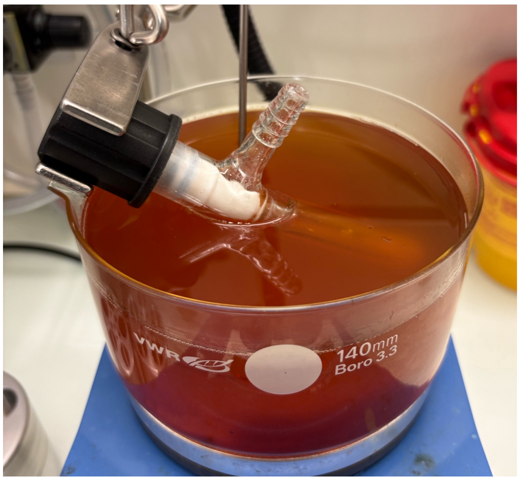

Figure S1: Reaction setup with the sealed Schlenk tube in the oil bath for the synthesis of the Bis-imidazolium salts **8-10**.

Bis-imidazolium salt 8

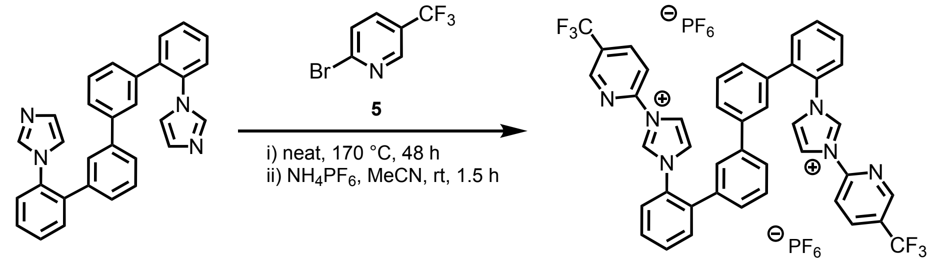

4 **8**

Following the general procedure [**A**], ligand **8** (1.80 g, 1.77 mmol, 94%) was obtained as a light brown solid starting from the precursor **4** (0.81 g, 1.86 mmol).

MP: 169 °C.

^{1}H-NMR: (500 MHz, CD_3CN) δ (ppm) = 9.57 (t, J = 1.7 Hz, 1H), 8.91 (dt, J = 2.6, 0.9 Hz, 1H), 8.48–8.43 (m, 1H), 8.16–8.12 (m, 1H), 7.95 – 7.90 (m, 1H), 7.83–7.71 (m, 4H), 7.65–7.55 (m, 2H), 7.52 (dd, J = 2.2, 1.7 Hz, 1H), 7.44 (t, J = 7.7 Hz, 1H), 7.25 (ddd, J = 7.7, 1.8, 1.1 Hz, 1H).

^{13}C-NMR: (126 MHz, CD_3CN) δ (ppm) = 149.5, 147.8, 147.7, 141.8, 139.3, 139.2, 138.3, 137.7, 136.2, 133.2, 132.8, 132.7, 132.7, 130.5, 130.5, 128.9, 128.6, 128.5, 128.3, 128.2, 127.5, 126.6, 120.4, 115.7.

^{19}F-NMR (282 MHz, CD_3CN) δ (ppm) = –62.94 (s, 3F), –72.82 (d, $^{1}J_{PF}$ = 706.5 Hz, 6F).

HRMS: ESI(+); *m/z* calculated for $C_{42}H_{28}F_{12}N_6P$ $[M–PF_6]^+$: 875.19, found: 875.1910 $[M–PF_6]^+$.

IR: ṽ (cm^{-1}) = 3159 (w), 1604 (w), 1542 (w), 1489 (w), 1412 (w), 1328 (m), 1261 (w), 1231 (w), 1173 (w), 1134 (m), 1078 (w), 1064 (w), 1018 (w), 970 (w), 956 (w), 821 (s), 760 (w), 740 (w), 708 (w), 677 (w), 657 (w), 614 (w), 555 (s), 514 (w), 490 (w), 468 (w), 427 (w).

Bis-imidazolium salt 9

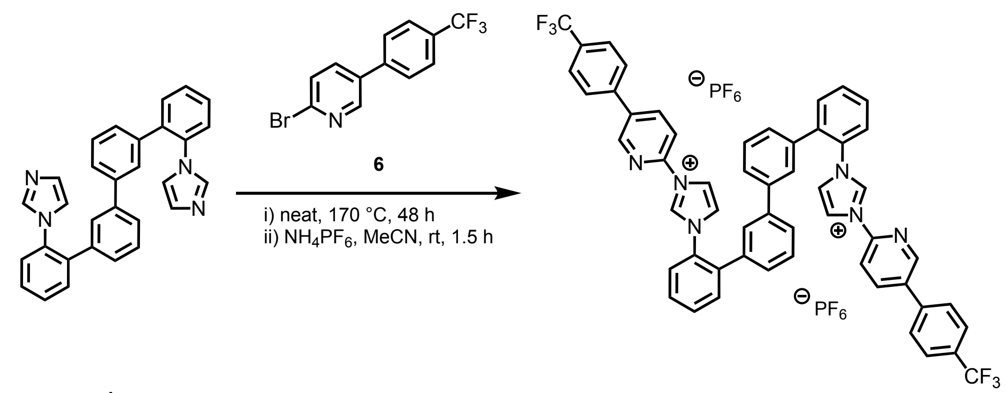

Following the general procedure [**A**], ligand **9** (255 mg, 0.22 mmol, 74%) was obtained as a light brown solid starting from the precursor **4** (130 mg, 0.29 mmol).

MP: 191 °C.

^{1}H-NMR: (500 MHz, CD_3CN) δ (ppm) = 9.51 (t, *J* = 1.7 Hz, 1H), 8.82 (dd, *J* = 2.4, 0.8 Hz, 1H), 8.37 (dd, *J* = 8.5, 2.4 Hz, 1H), 8.12 (dd, *J* = 2.2, 1.7 Hz, 1H), 7.92–7.86 (m, 4H), 7.82 (m, 1H), 7.67–7.66 (m, 4H), 7.61 (ddd, *J* = 7.8, 1.9, 1.1 Hz, 1H), 7.55–7.48 (m, 2H), 7.48–7.40 (m, 1H), 7.26 (ddd, *J* = 7.7, 1.8, 1.1 Hz, 1H).

^{13}C-NMR: (126 MHz, CD_3CN) δ (ppm) = 148.8, 146.7, 141.8, 140.6, 140.0, 138.4, 137.9, 137.8, 135.7, 133.4, 132.8, 132.7, 130.7, 130.5, 129.1, 129.1, 128.5, 128.2, 127.6, 127.1, 126.5, 120.4, 115.4.

^{19}F-NMR (282 MHz, CD_3CN) δ (ppm) = –63.12 (s, 3F), –72.68 (d, $^1J_{PF}$ = 706.5 Hz, 6F).

HRMS: ESI(+); *m/z* calculated for $C_{54}H_{36}F_6N_6$ $[M-(PF_6)_2]^+$: 441.14, found: 441.1447 $[M-(PF_6)_2]^+$.

IR: ṽ (cm^{-1}) = 3156 (w), 1618 (w), 1596 (w), 1541 (w), 1477 (w), 1424 (w), 1382 (w), 1324 (s), 1267 (w), 1167 (w), 1113 (m), 1068 (m), 1017 (w), 1004 (w), 971 (w), 957 (w), 818 (s), 762 (w), 739 (w), 707 (w), 683 (w), 655 (w), 614 (w), 604 (w), 555 (s), 512 (w), 483 (w), 431 (w).

Bis-imidazolium salt 10

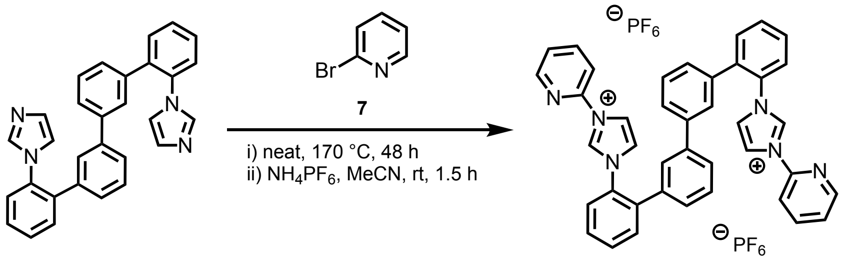

4 10

Following the general procedure [**A**], ligand **10** (160 mg, 0.18 mmol, 88%) was obtained as a light brown solid starting from the precursor **4** (90.0 mg, 0.21 mmol).

MP: 172 °C.

^{1}H-NMR: (500 MHz, CD_3CN) δ (ppm) = δ 9.42 (t, *J* = 1.7 Hz, 1H), 8.57–8.48 (m, 1H), 8.11– 8.05 (m, 2H), 7.78–7.68 (m, 6H), 7.59–7.54 (m, 2H), 7.50 (dt, *J* = 10.0, 1.9 Hz, 2H), 7.48 (t, *J* = 7.8 Hz, 1H), 7.24 (ddd, *J* = 7.7, 1.8, 1.1 Hz, 1H).

^{13}C-NMR: (126 MHz, CD_3CN) δ (ppm) = 150.5, 147.0, 141.8, 141.7, 141.5, 138.4, 137.9, 135.6, 133.4, 132.7, 132.6, 132.6, 132.5, 132.2, 131.0, 130.7, 130.6, 130.5, 129.0, 128.4, 128.1, 127.6, 126.8, 126.3, 120.3, 115.3.

^{19}F-NMR (282 MHz, CD_3CN) δ (ppm) = –72.77 (d, $^1J_{PF}$ = 706.9 Hz, 6F).

HRMS: ESI(+); *m/z* calculated for $C_{40}H_{30}F_6N_6P$ [M–(PF_6)]$^+$: 739.21, found: 739.2166 [M–(PF_6)]$^+$.

IR: ṽ (cm^{-1}) = 3158 (w), 1600 (w), 1578 (w), 1540 (w), 1474 (w), 1444 (m), 1335 (w), 1259 (w), 1227 (w), 1160 (w), 1095 (w), 1065 (w), 996 (w), 823 (s), 764 (w), 739 (w), 708 (w), 656 (w), 615 (w), 554 (s), 512 (w), 469 (w), 407 (w).

3. Synthesis of Racemic Iron Complexes

General Procedure B: Racemic Iron Complexes *rac*-FeNCCN1-3

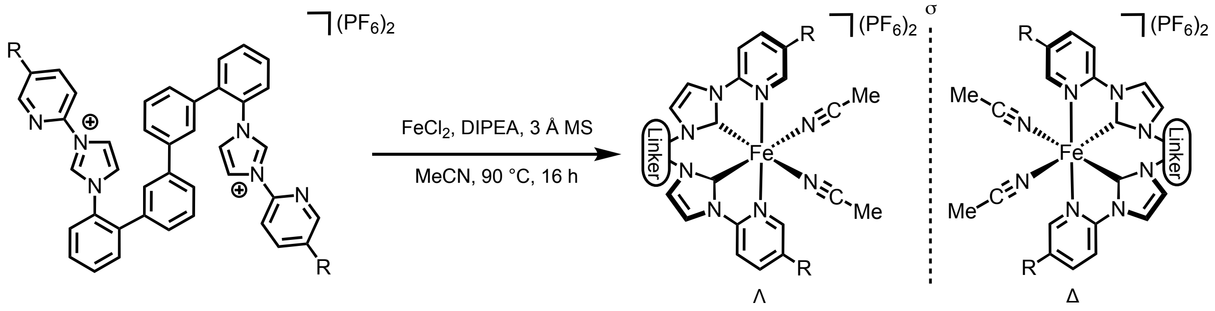

8 R = CF_3,
9 R = 4-CF_3Ph,
10 R = H,

rac-**FeNCCN1**: R = CF_3
rac-**FeNCCN2**: R = 4-CF_3Ph,
rac-**FeNCCN3**: R = H,

Following a modified procedure from the literature.[7] The ligand **8-10** (1.00 eq), $FeCl_2$ (1.00 eq) and molecular sieve (3 Å, 1 g per 1.0 mmol ligand) were suspended in dry and degassed MeCN (0.025 M based on the imidazolium salt) under an atmosphere of nitrogen. After stirring for 5 min, DIPEA (2.50 eq) was added, and the mixture was heated to 90 °C for 16 h. The red solution was allowed to cool to room temperature, diluted with MeCN, filtered over celite, and the solvent was removed under reduced pressure. The residue was purified by flash column chromatography (silica gel, CH_2Cl_2/MeCN) with an NH_4PF_6 pad on top of the column to ensure complete elution of the desired complex. After removing the solvent under reduced pressure, the crude product was dissolved in CH_2Cl_2/MeCN (10:1) and washed three times with H_2O to remove excess of PF_6^--salts. The combined organic phases were then dried over $MgSO_4$, filtered, and the solvent was removed under reduced pressure to afford the desired complexes *rac*-**FeNCCN1-3**.

Note: For the clean purification of the desired complexes during column chromatography, the silica gel has to be dispersed in the solvent mixture (CH_2Cl_2/MeCN) of the eluent.

rac-**FeNCCN1**

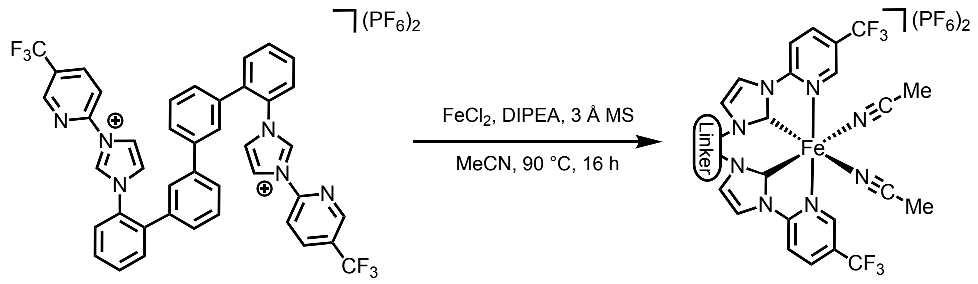

8 *rac*-**FeNCCN1**

Following the general procedure [**B**], *rac*-**FeNCCN1** (188 mg, 0.16 mmol, 40%) was obtained as a red solid starting from the ligand **8** (0.40 g, 0.39 mmol). Purification condition: CH_2Cl_2/MeCN = 10:1 → 4:1.

^{1}H-NMR: (500 MHz, CD_2Cl_2) δ (ppm) = 8.88 (d, *J* = 2.0 Hz, 1H), 7.88 (dd, *J* = 8.7, 2.1 Hz, 1H), 7.70 7.65 (m, 2H), 7.59 (t, *J* = 7.7 Hz, 1H), 7.52 (dd, *J* = 7.7, 1.4 Hz, 1H), 7.40 (d, *J* = 1.4 Hz, 1H), 7.34–7.27 (m, 3H), 7.23 (dd, *J* = 7.8, 1.4 Hz, 1H), 6.29 (d, *J* = 2.3 Hz, 1H), 6.20 (d, *J* = 1.9 Hz, 1H), 2.58 (s, 3H).

^{13}C-NMR: (126 MHz, CD_2Cl_2) δ (ppm) = 196.9, 157.0, 151.8, 139.3, 137.7, 136.2, 134.7, 132.9, 130.6, 130.4, 129.8, 129.2, 128.8, 128.0, 126.8, 125.8, 118.2, 110.8, 4.5.

^{19}F-NMR (282 MHz, CD_2Cl_2) δ (ppm) = –62.18 (s, 3 F), –72.81 (d, $^{1}J_{PF}$ = 711.0 Hz, 6 F).

HRMS: ESI(+); *m*/*z* calculated for $C_{42}H_{26}F_6N_8FeF$ $[M–(PF_6)_2–(MeCN)_2+F]^+$: 803.14, found: 803.1442 $[M–(PF_6)_2–(MeCN)_2+F]^+$.

IR: ṽ (cm^{-1}) = 3149 (w), 2920 (w), 2851 (w), 1621 (w), 1508 (w), 1474 (w), 1425 (w), 1326 (m), 1306 (w), 1270 (w), 1253 (w), 1175 (w), 1135 (w), 1074 (m), 1042 (w), 947 (w), 824 (s), 772 (w), 763 (w), 748 (w), 705 (w), 680 (w), 665 (w), 620 (w), 600 (w), 555 (s), 508 (w), 469 (w), 435 (w).

rac-**FeNCCN2**

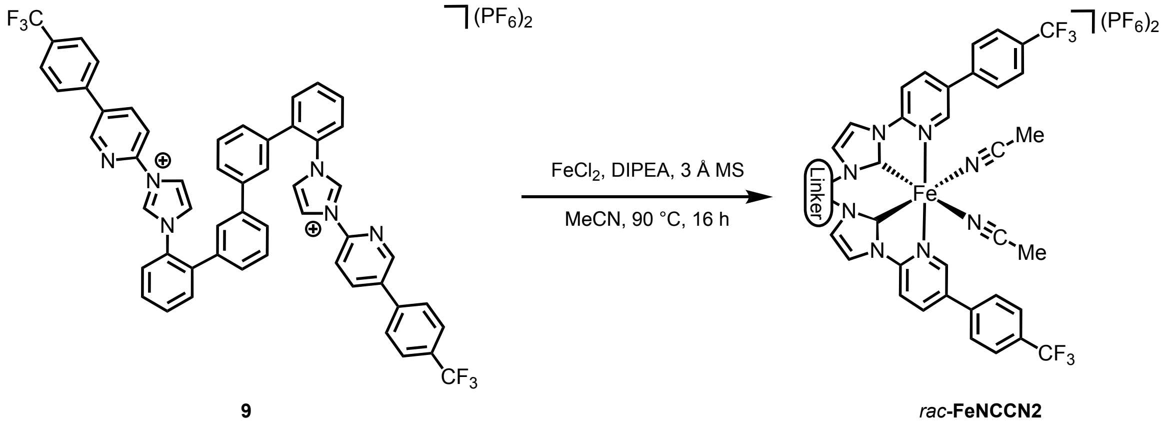

Following the general procedure [**B**], *rac*-**FeNCCN2** (53.5 mg, 0.04 mmol, 43%) was obtained as a red solid starting from the ligand **9** (0.11 g, 0.09 mmol). Purification condition: CH_2Cl_2/MeCN = 20:1 → 10:1.

^{1}H-NMR: (500 MHz, CD_3CN) δ (ppm) = 8.95 (d, *J* = 2.0 Hz, 1H), 8.01 (q, *J* = 8.4 Hz, 4H), 7.94 (dd, *J* = 8.5, 2.1 Hz, 1H), 7.75 (dt, *J* = 4.7, 1.9 Hz, 2H), 7.61 (t, *J* = 7.7 Hz, 1H), 7.54 (dd, *J* = 7.7, 1.4 Hz, 1H), 7.43–7.36 (m, 2H), 7.36–7.29 (m, 2H), 6.92 (td, *J* = 7.6, 1.4 Hz, 1H), 6.38 (d, *J* = 2.1 Hz, 1H), 6.34 (t, *J* = 1.8 Hz, 1H), 1.96 (s, 3H).

^{13}C-NMR: (126 MHz, CD_3CN) δ (ppm) = 198.5, 155.2, 154.3, 140.3, 140.1, 139.2, 137.9, 136.8, 136.0, 134.6, 132.9, 131.1, 131.1, 130.8, 130.8, 130.7, 130.1, 130.0, 129.7, 129.6, 129.2, 127.9, 127.2, 127.2, 127.1, 127.1, 126.4, 126.3, 124.3, 122.1, 118.9, 111.2.

^{19}F-NMR (282 MHz, CD_2Cl_2) δ (ppm) = –62.98 (s, 3 F), –72.84 (d, $^1J_{PF}$ = 706.6 Hz, 6 F).

HRMS: ESI(+); *m/z* calculated for $C_{58}H_{40}F_6N_8Fe$ $[M–(PF_6)_2]^+$: 509.13, found: 509.1303 $[M–(PF_6)_2]^+$.

IR: ṽ (cm^{-1}) = 1609 (w), 1525 (w), 1495 (w), 1474 (w), 1427 (w), 1325 (m), 1254 (w), 1167 (w), 1114 (w), 1071 (m), 1012 (w), 947 (w), 837 (s), 772 (w), 763 (w), 748 (w), 720 (w), 706 (w), 692 (w), 616 (w), 557 (m), 532 (w).

rac-**FeNCCN3**

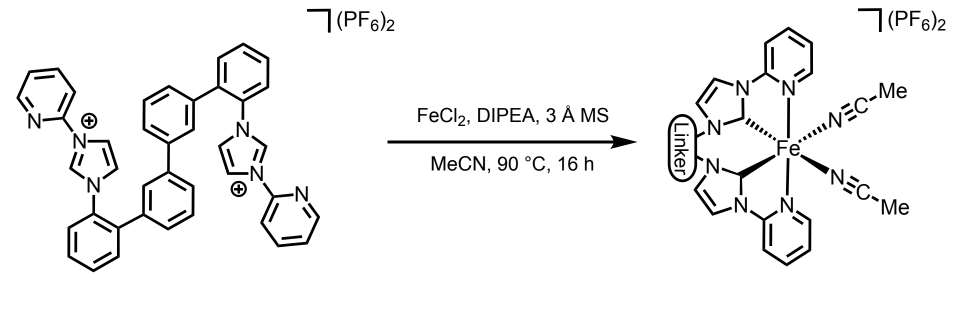

10 *rac*-**FeNCCN3**

Following the general procedure [**B**], *rac*-**FeNCCN3** (86.3 mg, 0.08 mmol, 56%) was obtained as a red solid starting from the ligand **10** (0.13 g, 0.03 mmol). Purification condition: CH_2Cl_2/MeCN = 10:1 → 4:1.

^{1}H-NMR: (500 MHz, CD_3CN) δ (ppm) = 8.82 (dd, *J* = 5.8, 1.5 Hz, 1H), 7.72–7.66 (m, 2H), 7.64 (t, *J* = 1.5 Hz, 1H), 7.59 (t, *J* = 7.7 Hz, 1H), 7.48 (dd, *J* = 7.8, 1.5 Hz, 1H), 7.39 (d, 1H), 7.37–7.32 (m, 2H), 7.32–7.26 (m, 1H), 7.23 (t, 1H), 7.19 (d, *J* = 8.2 Hz, 1H), 6.36 (d, *J* = 2.2 Hz, 1H), 6.30 (d, *J* = 1.9 Hz, 1H), 1.96 (s, 3H).

^{13}C-NMR: (126 MHz, CD_3CN) δ (ppm) = 198.8, 156.6, 155.5, 140.4, 140.1, 138.0, 136.5, 136.1, 132.4, 131.0, 130.7, 130.5, 130.5, 129.8, 129.8, 129.6, 128.0, 126.1, 123.4, 118.6, 110.9.

^{19}F-NMR (282 MHz, CD_3CN) δ (ppm) = –72.82 (d, $^1J_{PF}$ = 706.8 Hz, 6 F).

HRMS: ESI(+); *m/z* calculated for $C_{40}H_{28}FN_6Fe$ $[M–(PF_6)_2–(MeCN)_2+F]^+$: 667.16, found: 667.1693 $[M–(PF_6)_2–(MeCN)_2+F]^+$.

IR: ṽ (cm^{-1}) = 1614 (w), 1578 (w), 1489 (w), 1474 (w), 1455 (w), 1415 (w), 1333 (w), 1305 (w), 1269 (w), 1233 (w), 1162 (w), 1137 (w), 1088 (w), 947 (w), 835 (s), 762 (w), 748 (w), 723 (w), 705 (w), 643 (w), 616 (w), 556 (m), 512 (w), 429 (w).

4. Synthesis of Auxiliary Complexes

General Procedure C: Synthesis of Auxiliary Complexes Λ-(*R*)-**FeAux1-3** and Δ-(*R*)-**FeAux1-3**

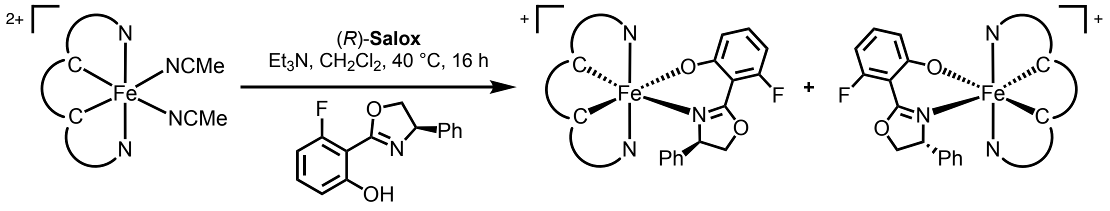

rac-**FeNCCN1-3** (*R*)-**Salox** Λ-(*R*)-**FeAux1-3** Δ-(*R*)-**FeAux1-3**

Following a procedure from the literature.[8] The racemic iron complexes (1.00 eq) and the corresponding Salox auxiliary (1.05 eq) were dissolved in dry CH_2Cl_2 (0.04 M based on the iron complex) under an atmosphere of nitrogen. Et_3N (1.50 eq) was added, and the reaction mixture was stirred at 40 °C for 16 h. The solvent was then removed under reduced pressure, and the residue was purified by flash column chromatography (silica gel, CH_2Cl_2/MeCN, 100:1 → 75:1 → 50:1 → 25:1) to obtain both diastereomers of the auxiliary complexes.

Note: For the separation of the auxiliary complexes via column chromatography, the silica gel has to be dispersed in pure CH_2Cl_2 instead of the solvent mixture (CH_2Cl_2/MeCN) of the eluent. During column chromatography, the complexes Λ-(*R*)-**FeAux1-3** elute first and partially decompose (Indicated by the slightly lighter purple color and the fading of the band on the column). To prevent contamination of the subsequent complexes Δ-(*R*)-**FeAux1-3**, the eluent gradient should only be increased gradually and carefully until a clear separation is visible (See figure S2).

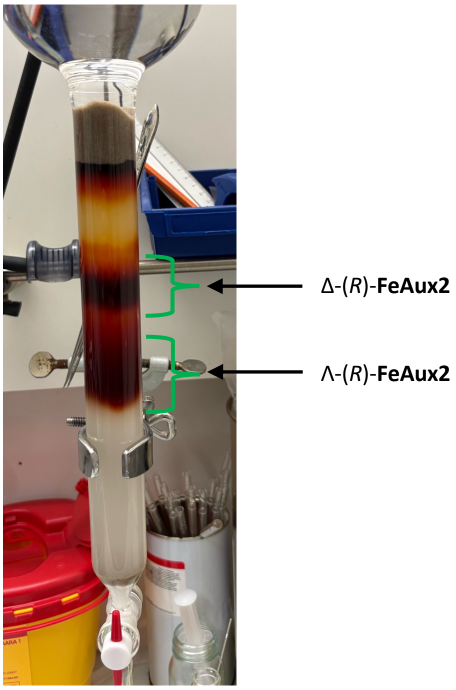

Figure S2: Separation of the auxiliary complexes via column chromatography, shown by the example of **FeAux2**, to visualize the separation and the decomposition of the less stable Λ-diastereomer.

Λ-(*R*)-**FeAux1** and Δ-(*R*)-**FeAux1**

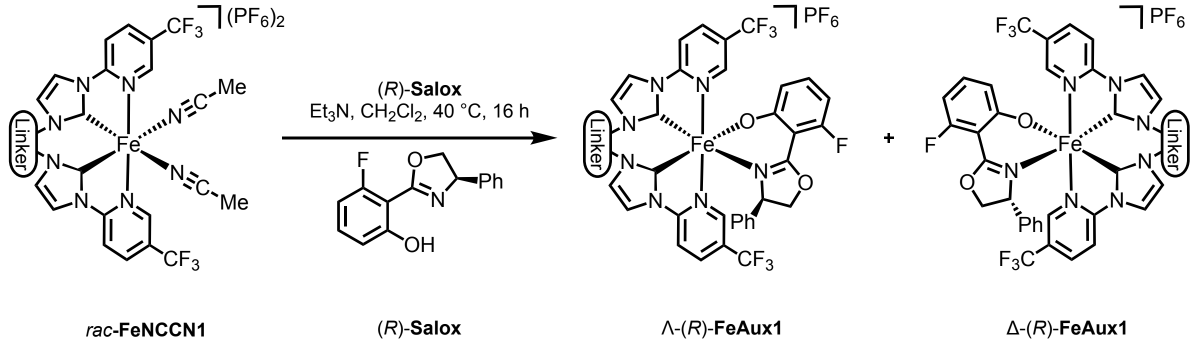

Following the general procedure [**C**], Λ-(*R*)-**FeAux1** (25.4 mg, 21.4 µmol, 33% (66% of theory)) was obtained as a purple solid from the corresponding complex *rac*-**FeNCCN1** (75.0 mg, 65.0 µmol). The other diastereomer Δ-(*R*)-**FeAux1** (26.4 mg, 22.3 µmol, 34% (68% of theory)) was obtained as a purple solid. Purification condition: CH_2Cl_2/MeCN = 100:1 → 75:1 → 50:1 → 25:1.

Analytical data for Λ-(*R*)-**FeAux1**:

^{1}H-NMR: (500 MHz, CD_2Cl_2) δ (ppm) = 8.63 (s, 1H), 8.42 (s, 1H), 7.98 (d, *J* = 7.8 Hz, 1H), 7.71 (d, *J* = 8.6 Hz, 1H), 7.66 (d, *J* = 7.9 Hz, 1H), 7.62 – 7.50 (m, 5H), 7.41 – 7.20 (m, 10H), 7.18 – 7.08 (m, 3H), 6.93 (t, *J* = 7.5 Hz, 1H), 6.81 (s, 2H), 6.69 (d, *J* = 8.6 Hz, 1H), 6.37 (d, *J* = 8.5 Hz, 1H), 6.31 (d, *J* = 8.8 Hz, 3H), 6.27 (s, 1H), 6.21 (s, 1H), 6.15 (t, *J* = 10.1 Hz, 1H), 5.99 (s, 1H), 5.08 – 5.02 (m, 1H), 4.45 (dd, *J* = 9.4, 3.5 Hz, 1H).

^{13}C-NMR: (126 MHz, CD_2Cl_2) δ (ppm) = 159.1, 157.2, 151.7, 151.7, 151.4, 140.2, 139.9, 139.7, 137.3, 136.9, 136.7, 135.9, 135.8, 135.6, 135.0, 133.7, 131.5, 130.9, 130.8, 130.6, 130.5, 130.1, 129.9, 129.8, 129.5, 129.2, 129.0, 128.9, 128.9, 128.8, 127.6, 127.4, 126.6, 126.0, 126.0, 125.8, 124.1, 123.9, 123.9, 123.4, 123.1, 122.1, 122.0, 117.7, 117.4, 110.1, 109.1, 76.1, 73.8.

^{19}F-NMR: (282 MHz, CD_2Cl_2) δ (ppm) = –62.31 (s, 3F), –62.47 (s, 3F), –73.07 (d, $^{1}J_{PF}$ = 710.9 Hz, 6F), –106.07 (s, 1F).

HRMS: ESI(+); *m/z* calculated for $C_{57}H_{37}F_7FeN_7O_2$ $[M–(PF_6)]^+$: 1040.22, found: 1040.2252 $[M–(PF_6)]^+$.

IR: ṽ (cm^{-1}) = 3144 (w), 3085 (w), 1615 (m), 1577 (m), 1531 (w), 1505 (m), 1474 (w), 1447 (m), 1420 (w), 1387 (w), 1326 (s), 1312 (w), 1301 (w), 1256 (w), 1244 (w), 1233 (w), 1172 (w), 1133 (m), 1099 (w), 1072 (w), 1042 (w), 1004 (w), 991 (w), 954 (w), 944 (m), 924 (w), 832 (s), 795 (w), 771 (w), 762 (w), 747 (m), 702 (m), 677 (w), 661 (w), 620 (w), 600 (w), 582 (w), 557 (s), 534 (w), 510 (w), 462 (w).

Analytical data for Δ-(*R*)-**FeAux1**:

^{1}H-NMR: (500 MHz, CD_2Cl_2) δ (ppm) = 8.84 (s, 1H), 8.70 (s, 1H), 8.08 (d, *J* = 7.7 Hz, 1H), 7.90 (dd, *J* = 8.6, 2.1 Hz, 1H), 7.85 (d, *J* = 2.4 Hz, 1H), 7.68–7.59 (m, 3H), 7.59 – 7.51 (m, 5H), 7.52–7.44 (m, 2H), 7.37 (dtd, *J* = 7.6, 3.7, 1.4 Hz, 2H), 7.32 – 7.23 (m, 2H), 7.29–7.24 (m, 4H), 7.19–7.16 (m, 1H), 7.03 (td, *J* = 7.6, 1.2 Hz, 1H), 6.92 (dt, *J* = 18.0, 8.5 Hz, 2H), 6.41 (d, *J* = 1.9 Hz, 1H), 6.33 (td, *J* = 7.8, 1.6 Hz, 2H), 6.24 (d, *J* = 2.2 Hz, 1H), 6.18 (t, *J* = 9.6 Hz, 1H), 6.13 (d, *J* = 2.3 Hz, 1H), 5.97 (d, *J* = 1.9 Hz, 1H), 4.64 (dd, *J* = 9.3, 3.4 Hz, 1H), 4.46 (d, *J* = 7.7 Hz, 1H), 4.13 (t, *J* = 9.4 Hz, 1H), 3.46 (d, *J* = 9.4 Hz, 1H).

^{13}C-NMR: (126 MHz, CD_2Cl_2) δ (ppm) = 165.8, 163.2, 161.2, 158.7, 157.8, 153.4, 153.3, 151.5, 141.7, 139.9, 139.8, 137.5, 136.8, 136.1, 135.8, 135.7, 135.6, 135.6, 135.5, 135.1, 135.0, 133.4, 133.3, 131.4, 130.7, 130.6, 130.5, 130.3, 130.2, 130.0, 129.9, 129.5, 128.9, 128.8, 128.6, 128.5, 128.1, 128.0, 127.8, 126.2, 125.9, 125.5, 124.6, 124.3, 124.1, 123.2, 123.0, 121.9, 117.5, 117.5, 109.6, 109.0, 100.9, 100.7, 77.1, 69.1.

^{19}F-NMR: (282 MHz, CD_2Cl_2) δ (ppm) = –61.97 (s, 3F), –62.48 (s, 3F), –72.90 (d, $^1J_{PF}$ = 711.0 Hz, 6F), –110.35 (s, 1F).

HRMS: ESI(+); *m/z* calculated for $C_{57}H_{37}F_7FeN_7O_2$ $[M–(PF_6)]^+$: 1040.22, found: 1040.2217 $[M–(PF_6)]^+$.

IR: ṽ (cm^{-1}) = 3144 (w), 3077 (w), 1619 (m), 1588 (w), 1536 (w), 1504 (m), 1475 (w), 1447 (m), 1419 (m), 1386 (w), 1356 (w), 1326 (s), 1311 (w), 1283 (w), 1262 (w), 1233 (m), 1171 (w), 1136 (m), 1099 (w), 1085 (w), 1072 (w), 1038 (m), 1006 (w), 992 (w), 944 (m), 913 (w), 838 (s), 796 (w), 772 (w), 765 (m), 704 (m), 676 (w), 663 (w), 621 (w), 599 (w), 586 (w), 557 (s), 533 (w), 512 (w), 471 (w).

Λ-(*R*)-**FeAux2** and Δ-(*R*)-**FeAux2**

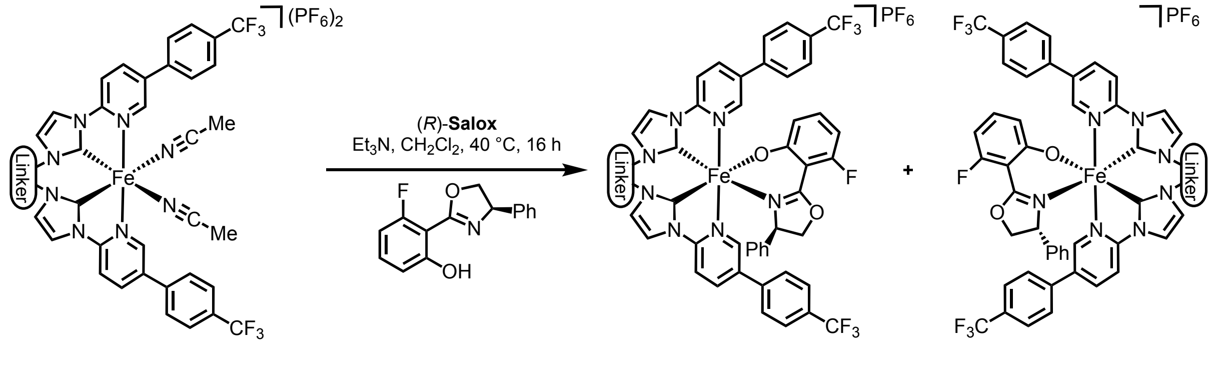

rac-**FeNCCN2** (*R*)-**Salox** Λ-(*R*)-**FeAux2** Δ-(*R*)-**FeAux2**

Following the general procedure [**C**], Λ-(*R*)-**FeAux2** (17.0 mg, 12.7 µmol, 33% (66% of theory)) was obtained as a purple solid from the corresponding complex *rac*-**FeNCCN2** (51.3 mg, 39.0 µmol).

The other diastereomer Δ-(*R*)-**FeAux2** (16.5 mg, 12.3 µmol, 32% (64% of theory)) was obtained as a purple solid. Purification condition: CH_2Cl_2/MeCN = 100:1 → 75:1 → 50:1 → 25:1.

Analytical data for Λ-(*R*)-**FeAux2**:

^{1}H-NMR: (500 MHz, CD_2Cl_2) δ (ppm) = 8.82 (s, 1H), 8.70 (s, 1H), 8.02 (d, *J* = 7.6 Hz, 1H), 7.94 (d, *J* = 8.0 Hz, 2H), 7.91 (dd, *J* = 8.4, 2.2 Hz, 1H), 7.85 (d, *J* = 7.9 Hz, 2H), 7.80 (s, 1H), 7.71–7.64 (m, 3H), 7.64–7.48 (m, 7H), 7.40 (dd, *J* = 24.6, 7.7 Hz, 3H), 7.28–7.20 (m, 3H), 7.06–6.90 (m, 5H), 6.89–6.81 (m, 1H), 6.77 (t, *J* = 7.6 Hz, 1H), 6.48 (s, 1H), 6.37 (d, *J* = 8.5 Hz, 1H), 6.22 (s, 2H), 6.10 (s, 1H), 6.07–6.00 (m, 2H), 4.68 – 4.61 (m, 1H), 4.51 (d, *J* = 7.8 Hz, 1H), 4.19 (t, *J* = 9.4 Hz, 1H), 3.63 (s, 1H).

^{13}C-NMR: (126 MHz, CD_2Cl_2) δ (ppm) = 155.8, 155.1, 154.7, 139.9, 139.8, 139.7, 137.7, 137.1, 136.5, 136.3, 136.2, 135.8, 135.7, 135.3, 133.3, 132.2, 131.5, 131.1, 131.0, 130.9, 130.8, 130.6, 130.5, 130.1, 130.1, 129.8, 129.8, 129.4, 128.6, 128.6, 128.5, 128.4, 128.2, 128.1, 127.7, 127.3, 127.2, 127.0, 127.0, 126.9, 126.9, 125.8, 125.6, 125.6, 125.5, 123.5, 123.4, 117.0, 116.9, 109.3, 109.1, 77.0, 69.4, 30.1.

^{19}F-NMR: (282 MHz, CD_2Cl_2) δ (ppm) = –62.86 (s, 3F), –62.92 (s, 3F), –73.13 (d, $^{1}J_{PF}$ = 711.0 Hz, 6F), –110.33 (s, 1F).

HRMS: ESI(+); *m/z* calculated for $C_{69}H_{45}F_7FeN_7O_2$ $[M–(PF_6)]^+$: 1192.29, found: 1192.2869 $[M–(PF_6)]^+$.

IR: ṽ (cm^{-1}) = 3143 (w), 3062 (w), 2924 (w), 2854 (w), 1620 (m), 1588 (w), 1526 (w), 1493 (m), 1475 (w), 1450 (w), 1423 (w), 1385 (w), 1365 (w), 1325 (s), 1282 (w), 1260 (w), 1233 (m), 1169 (w), 1127 (m), 1071 (m), 1038 (w), 1013 (w), 993 (w), 945 (w), 913 (w), 841 (s), 795 (w), 773 (w), 764 (w), 753 (w), 705 (m), 676 (w), 662 (w), 608 (w), 557 (m), 532 (w), 608 (w), 588 (w), 551 (w), 527 (w), 497 (w), 487 (w), 431 (w).

Analytical data for Δ-(*R*)-**FeAux2**

^{1}H-NMR: (500 MHz, CD_2Cl_2) δ (ppm) = 8.57 (s, 1H), 8.37 (s, 1H), 8.09–7.86 (m, 3H), 7.82 (d, *J* = 7.8 Hz, 2H), 7.72–7.64 (m, 4H), 7.63–7.56 (m, 2H), 7.55–7.47 (m, 4H), 7.45 (s, 1H), 7.41–7.27 (m, 7H), 7.21–7.14 (m, 2H), 7.09 (t, *J* = 7.6 Hz, 1H), 7.00 (d, *J* = 8.5 Hz, 1H), 6.87 (q, *J* = 7.7 Hz, 2H), 6.73 (s, 2H), 6.65 (t, *J* = 7.6 Hz, 1H), 6.39–6.34 (m, 3H), 6.31 (d, *J* = 8.4 Hz, 1H), 6.20 (s, 1H), 6.15 (s, 1H), 6.07 (d, *J* = 2.2 Hz, 1H), 5.61 – 5.44 (m, 1H), 5.00 (s, 1H), 4.44 (d, *J* = 9.3 Hz, 1H).

^{13}C-NMR: (126 MHz, CD_2Cl_2) δ (ppm) = 156.1, 154.4, 153.0, 153.0, 139.9, 139.9, 139.7, 139.7, 137.6, 137.1, 136.9, 136.8, 136.1, 136.1, 135.7, 135.4, 132.9, 131.8, 131.3, 131.1, 130.7, 130.5, 130.5, 129.8, 129.7, 129.6, 129.1, 129.0, 128.9, 128.8, 128.6, 128.5, 128.3, 127.7, 127.5, 127.5, 127.2, 127.2, 127.0, 126.8, 126.8, 126.7, 125.8, 125.7, 125.6, 125.6, 123.5, 123.4, 117.0, 116.7, 109.8, 109.1, 76.0, 30.1.

^{19}F-NMR: (282 MHz, CD_2Cl_2) δ (ppm) = –62.87 (s, 3F), –62.91 (s, 3F), –73.16 (d, $^1J_{PF}$ = 711.0 Hz, 6F), –106.10 (s, 1F).

HRMS: ESI(+); *m/z* calculated for $C_{69}H_{45}F_7FeN_7O_2$ $[M–(PF_6)]^+$: 1192.29, found: 1192.2868 $[M–(PF_6)]^+$.

IR: ṽ (cm^{-1}) = 3144 (w), 3063 (w), 2924 (w), 2853 (w), 1617 (m), 1578 (m), 1529 (w), 1494 (m), 1474 (w), 1450 (w), 1425 (w), 1387 (w), 1325 (s), 1282 (w), 1246 (w), 1232 (w), 1169 (w), 1127 (m), 1072 (m), 1042 (w), 1012 (w), 991 (w), 946 (w), 923 (w), 840 (s), 795 (w), 772 (w), 762 (w), 748 (w), 705 (m), 676 (w), 607 (w), 558 (m), 532 (w), 505 (w),

Λ-(*R*)-**FeAux3** and Δ-(*R*)-**FeAux3**

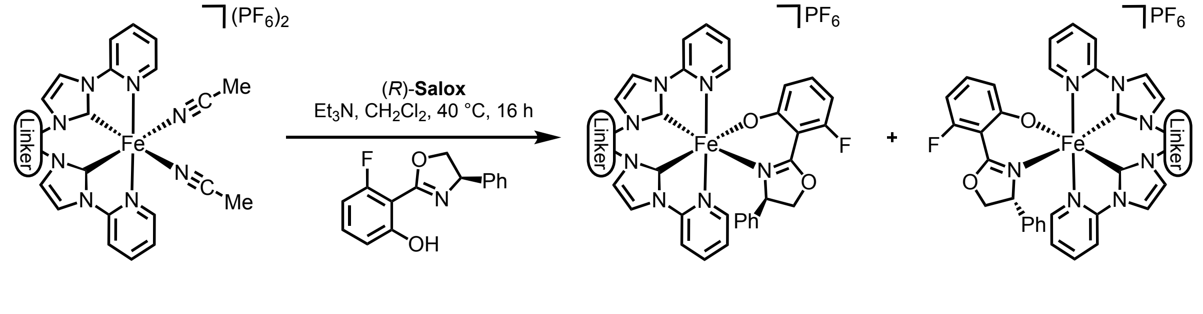

rac-**FeNCCN3** (*R*)-**Salox** Λ-(*R*)-**FeAux3** Δ-(*R*)-**FeAux3**

Following the general procedure [**C**], Λ-(*R*)-**FeAux3** (21.1 mg, 20.1 µmol, 33% (66% of theory)) was obtained as a purple solid from the corresponding complex *rac*-**FeNCCN2** (63.0 mg, 62.0 µmol). The other diastereomer Δ-(*R*)-**FeAux3** (22.4 mg, 21.3 µmol, 35% (70% of theory)) was obtained as a purple solid. Purification condition: CH_2Cl_2/MeCN = 100:1 → 75:1 → 50:1 → 25:1.

Analytical data for Λ-(*R*)-**FeAux3**:

^{1}H-NMR: (500 MHz, CD_2Cl_2) δ (ppm) = 8.24 (s, 1H), 8.01 (d, *J* = 7.8 Hz, 1H), 7.74 (s, 1H), 7.65 (d, *J* = 8.2 Hz, 2H), 7.60 (d, *J* = 7.7 Hz, 1H), 7.55 (t, *J* = 7.6 Hz, 2H), 7.44 (d, *J* = 7.6 Hz, 1H), 7.36 (d, *J* = 7.5 Hz, 2H), 7.33–7.13 (m, 10H), 7.04 (t, *J* = 6.5 Hz, 3H), 6.95 (t, *J* = 7.5 Hz, 2H), 6.89 (t, *J* = 6.5 Hz, 2H), 6.62 (d, *J* = 8.1 Hz, 1H), 6.48 (s, 1H), 6.30 (d, *J* = 8.6 Hz, 1H), 6.25 (t, *J* = 7.6 Hz, 1H), 6.09 (s, 1H), 6.06–6.00 (m, 2H), 5.92 (s, 1H), 4.56–4.48 (m, 2H), 4.04 (t, *J* = 9.3 Hz, 1H), 3.75 (s, 1H).

^{13}C-NMR: (126 MHz, CD_2Cl_2) δ (ppm) = 156.1, 155.5, 155.3, 154.6, 142.6, 139.9, 139.8, 138.4, 137.9, 137.7, 137.2, 136.3, 135.4, 135.2, 131.5, 130.5, 130.4, 130.0, 129.8, 129.7, 129.7, 129.5, 129.1, 128.5, 128.4, 128.3, 128.3, 128.1, 127.5, 126.8, 125.6, 125.3, 121.4, 120.2, 116.6, 116.4, 108.7, 76.4, 68.9.

^{19}F-NMR: (282 MHz, CD_2Cl_2) δ (ppm) = –73.28 (d, $^1J_{PF}$ = 710.7 Hz, 6F), –108.94 (s, 1F).

HRMS: ESI(+); *m/z* calculated for $C_{55}H_{39}FFeN_7O_2$ [M–(PF_6)]$^+$: 904.25, found: 904.2495 [M–(PF_6)]$^+$.

IR: ṽ (cm^{-1}) = 3140 (w), 3054 (w), 2002 (w), 1980 (w), 1618 (m), 1583 (w), 1533 (w), 1485 (m), 1454 (w), 1425 (w), 1410 (w), 1384 (w), 1365 (w), 1319 (w), 1301 (w), 1281 (w), 1261 (w), 1231 (m), 1156 (w), 1132 (w), 1120 (w), 1098 (w), 1084 (w), 1038 (w), 993 (w), 944 (w), 913 (w), 875 (w), 836 (s), 794 (w), 761 (m), 749 (w), 702 (m), 672 (w), 644 (w), 616 (w), 594 (w), 586 (w), 571 (w), 555 (m), 532 (w), 510 (w), 461 (w), 443 (w).

Analytical data for Δ-(*R*)-**FeAux3**

^{1}H-NMR: (500 MHz, CD_2Cl_2) δ (ppm) = 8.17 (s, 1H), 8.01 (d, *J* = 7.7 Hz, 1H), 7.84 (s, 1H), 7.61 (d, *J* = 7.9 Hz, 1H), 7.58–7.52 (m, 2H), 7.49 (t, *J* = 2.6 Hz, 1H), 7.44 (d, *J* = 7.7 Hz, 1H), 7.40 (t, *J* = 8.0 Hz, 1H), 7.35-7.25 (m, *J* = 13.7, 6.1 Hz, 7H), 7.25–7.13 (m, 4H), 7.08 (dt, *J* = 21.7, 7.6 Hz, 2H), 6.99 (s, 1H), 6.85 (t, *J* = 7.6 Hz, 1H), 6.79–6.70 (m, 3H), 6.42 (s, 1H), 6.27 (s, 1H), 6.24 (s, 2H), 6.13–6.09 (m, 1H), 6.07–6.02 (m, 2H), 5.97 (s, 1H), 5.73 (s, 2H), 5.06 (t, *J* = 9.2 Hz, 1H), 4.31 (dd, *J* = 9.2, 3.6 Hz, 1H).

^{13}C-NMR: (126 MHz, CD_2Cl_2) δ (ppm) = 156.6, 154.9, 154.4, 154.3, 141.3, 139.9, 139.7, 139.1, 138.0, 137.0, 137.0, 136.3, 136.2, 136.1, 135.2, 131.3, 130.9, 130.6, 130.4, 129.8, 129.7, 129.5, 129.3, 129.2, 128.7, 128.7, 128.5, 128.0, 127.8, 127.8, 126.6, 125.7, 125.4, 121.3, 120.9, 116.6, 116.2, 109.2, 108.5, 75.4, 73.4.

^{19}F-NMR: (282 MHz, CD_2Cl_2) δ (ppm) = –73.16 (d, $^1J_{PF}$ = 710.7 Hz, 6F), –105.21 (s, 1F).

HRMS: ESI(+); *m/z* calculated for $C_{55}H_{39}FFeN_7O_2$ [M–(PF_6)]$^+$: 904.25, found: 904.2495 [M–(PF_6)]$^+$.

IR: ṽ (cm^{-1}) = 3142 (w), 3062 (w), 1615 (m), 1574 (m), 1529 (w), 1485 (w), 1473 (w), 1451 (m), 1426 (w), 1411 (w), 1387 (w), 1375 (w), 1324 (w), 1303 (w), 1280 (w), 1245 (w), 1233 (m), 1213 (w), 1160 (w), 1133 (w), 1100 (w), 1057 (w), 1040 (m), 1004 (w), 991 (w), 954 (w), 945 (w), 923 (w), 877 (w), 841 (s), 831 (w), 791 (w), 762 (m), 748 (w), 701 (m), 675 (w), 639 (w), 615 (w), 598 (w), 581 (w), 572 (w), 556 (s), 533 (w), 509 (w), 488 (w), 455 (w), 431 (w),

General Procedure D: One-Pot Method for the Synthesis of Λ-(*R*)-**FeAux1-3** and Δ-(*R*)-**FeAux1-3**

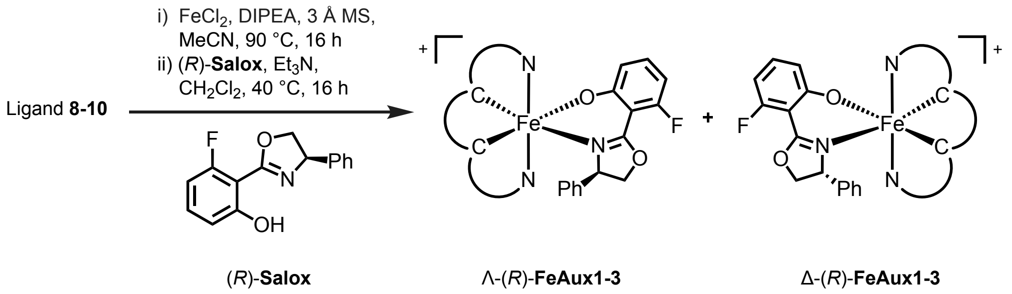

Following a modified procedure from the literature.[7,8] The ligand **8-10** (1.00 eq), $FeCl_2$ (1.00 eq), and molecular sieve (3 Å, 1 g per 1.0 mmol ligand) were suspended in dry and degassed MeCN (0.025 M based on the imidazolium salt) under an atmosphere of nitrogen. After stirring for 5 min, DIPEA (2.50 eq) was added, and the mixture was heated to 90 °C for 16 h.

The red solution was allowed to cool to room temperature, diluted with MeCN, filtered over celite, and the solvent was removed under reduced pressure. The crude iron complexes and the corresponding Salox auxiliary (1.05 eq) were dissolved in dry CH_2Cl_2 (0.04 M based on the imidazolium salt) under an atmosphere of nitrogen. Et_3N (1.50 eq) was added, and the reaction mixture was stirred at 40 °C for 16 h. The solvent was then removed under reduced pressure, and the residue was purified by flash column chromatography (silica gel, CH_2Cl_2/MeCN, 100:1 → 75:1 → 50:1 → 25:1) to obtain both diastereomers of the auxiliary complexes.

Note: The MeCN content of the eluent for column chromatography should not exceed 25:1, as otherwise DIPEA×PF_6^--salts would be dissolved.

Λ-(*R*)-**FeAux1** and Δ-(*R*)-**FeAux1**

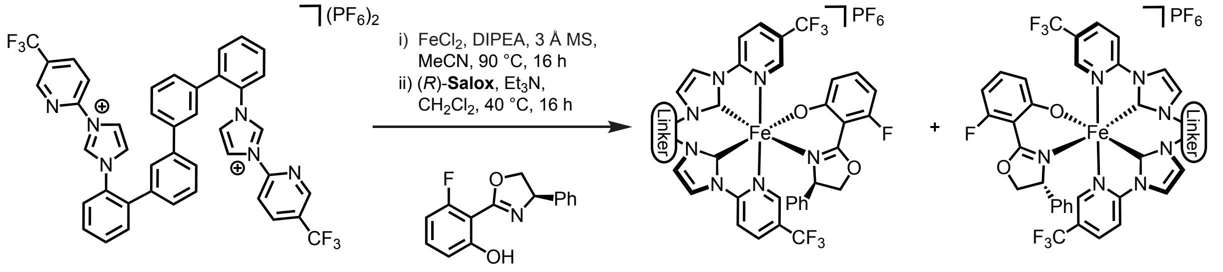

8 (*R*)-**Salox** Λ-(*R*)-**FeAux1** Δ-(*R*)-**FeAux1**

Following the general procedure [**D**], Λ-(*R*)-**FeAux1** (0.22 g, 0.18 mmol, 23% (46% of theory)) was obtained as a purple solid from the corresponding ligand **8** (0.80 g, 0.79 mmol). The other diastereomer Δ-(*R*)-**FeAux1** (0.21 g, 0.17 mmol, 22% (44% of theory)) was obtained as a purple solid. Purification condition: CH_2Cl_2/MeCN = 100:1 → 75:1 → 50:1 → 25:1. The spectroscopic data are consistent with those for the previous synthesis, starting from the racemic iron complexes

Λ-(*R*)-**FeAux2** and Δ-(*R*)-**FeAux2**

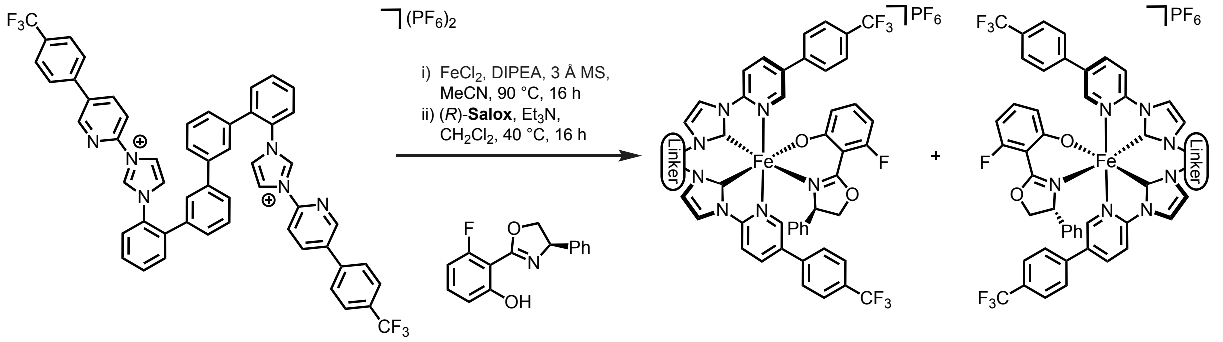

9 (*R*)-**Salox** Λ-(*R*)-**FeAux2** Δ-(*R*)-**FeAux2**

Following the general procedure [**D**], Λ-(*R*)-**FeAux2** (78.0 mg, 58.3 µmol, 27% (54% of theory)) was obtained as a purple solid from the corresponding ligand **9** (250 mg, 213 µmol). The other diastereomer Δ-(*R*)-**FeAux2** (73.0 mg, 54.6 µmol, 26% (52% of theory)) was obtained as a purple solid.

Purification condition: CH_2Cl_2/MeCN = 100:1 → 75:1 → 50:1 → 25:1. The spectroscopic data are consistent with those for the previous synthesis, starting from the racemic iron complexes

Λ-(*R*)-**FeAux3** and Δ-(*R*)-**FeAux3**

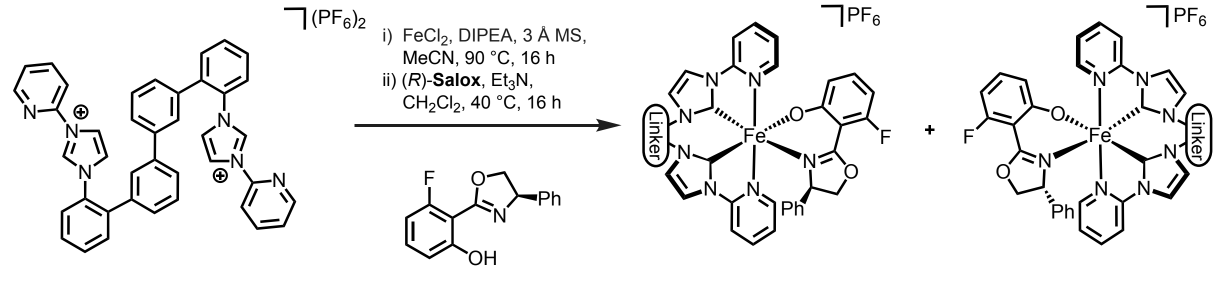

10 (*R*)-**Salox** Λ-(*R*)-**FeAux3** Δ-(*R*)-**FeAux3**

Following the general procedure [**D**], Λ-(*R*)-**FeAux3** (45.5 mg, 43.3 µmol, 12% (24% of theory)) was obtained as a purple solid from the corresponding ligand **10** (314 mg, 355 µmol). The other diastereomer Δ-(*R*)-**FeAux3** (49.9 mg, 47.6 µmol, 13% (26% of theory)) was obtained as a purple solid. Purification condition: CH_2Cl_2/MeCN = 100:1 → 75:1 → 50:1 → 25:1. The spectroscopic data are consistent with those for the previous synthesis, starting from the racemic iron complexes

5. Cleavage of the Chiral Auxiliary

General Procedure E: Cleavage of Salox Auxiliary

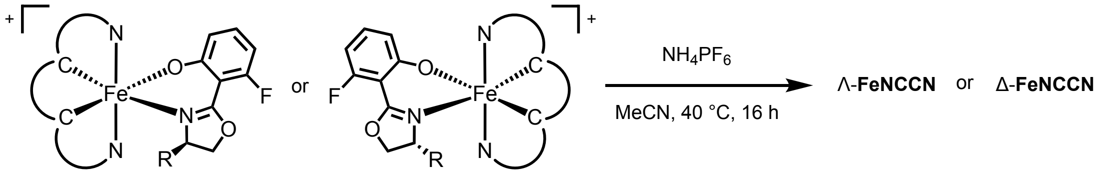

Λ-**FeAux** Δ-**FeAux**

Following a modified procedure from the literature.[8] The single diastereomers of the auxiliary complexes (1.00 eq) were dissolved in dry MeCN (0.02 M based on the auxiliary complex), and an excess of NH_4PF_6 (10.00 eq) was added, and the mixture was stirred at 40 °C for 16 h. Afterward, the solvent was removed under reduced pressure, and Et_2O was added. The precipitated solids were transferred to a celite pad and washed with Et_2O several times to remove any residues of free auxiliary. The complex was then eluted with CH_2Cl_2/MeCN, 30:1 to afford the corresponding Λ- or Δ-iron complexes after the solvent had been removed under reduced pressure.

Λ-FeNCCN1

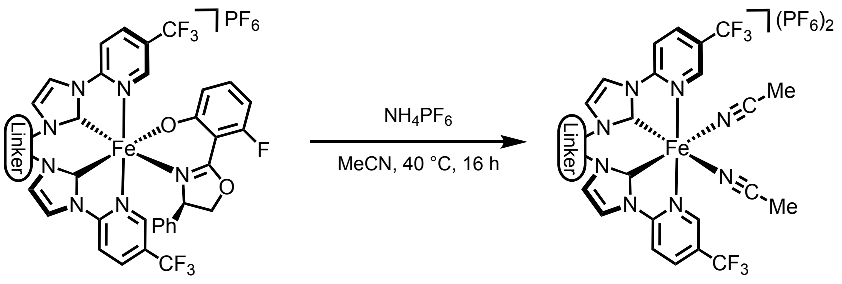

Λ-(*R*)-**FeAux1** Λ-**FeNCCN1**

Following the general procedure [**E**], Λ-**FeNCCN1** (55.0 mg, 47.6 µmol, 94%) was obtained as a red solid from the corresponding complex Λ-(*R*)-**FeAux1** (60.2 mg, 51.0 µmol). The spectroscopic data of the enantiopure complex were in accordance with *rac*-**FeNCCN1**.

CD: (MeCN, 0.25 mM): λ, nm (Δε, $M^{-1}cm^{-1}$) 234 (+98), 259 (−99), 282 (+60), 302 (−13), 330 (+4), 355 (+2), 390 (+16), 442 (−23), 517 (+4).

Δ-FeNCCN1

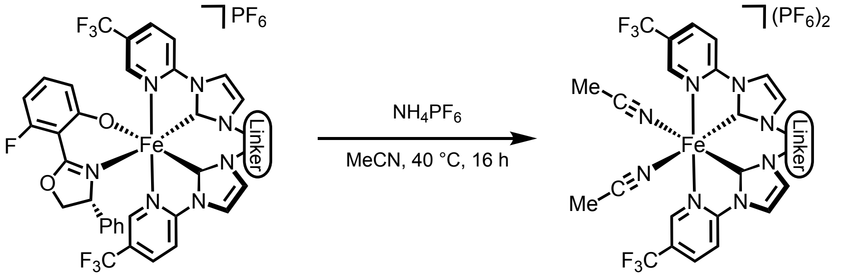

Δ-(*R*)-**FeAux1** Δ-**FeNCCN1**

Following the general procedure [**E**], Δ-**FeNCCN1** (66.0 mg, 57.1 µmol, 94%) was obtained as a red solid from the corresponding complex Δ-(*R*)-**FeAux1** (72.0 mg, 61.0 µmol). The spectroscopic data of the enantiopure complex were in accordance with *rac*-**FeNCCN1**.

CD: (MeCN, 0.25 mM): λ, nm (Δε, $M^{-1}cm^{-1}$) 234 (−106), 259 (+119), 282 (−71), 302 (+16), 330 (−5), 355 (−3), 390 (−16), 442 (+25), 517 (−4).

Λ-**FeNCCN2**

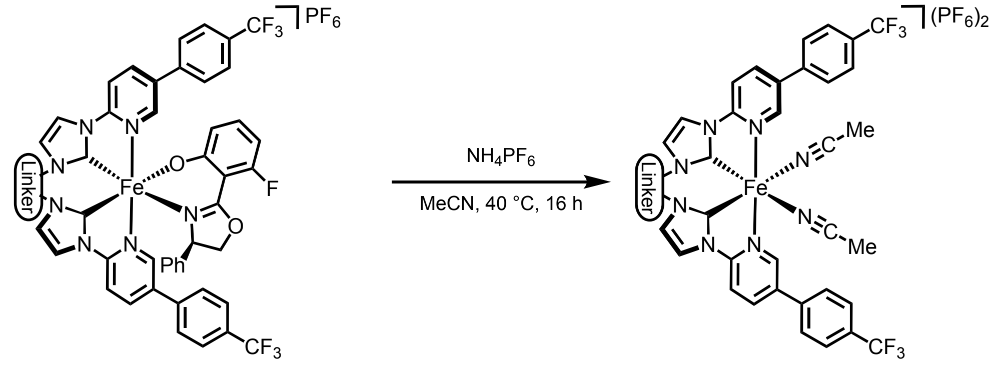

Λ-(*R*)-**FeAux2** Λ-**FeNCCN2**

Following the general procedure [**E**], Λ-**FeNCCN2** (51.5 mg, 39.3 µmol, 97%) was obtained as a red solid from the corresponding complex Λ-(*R*)-**FeAux2** (54.4 mg, 41.0 µmol). The spectroscopic data of the enantiopure complex were in accordance with *rac*-**FeNCCN2**.

CD: (MeCN, 0.25 mᴍ): λ, nm (Δε, $M^{-1}cm^{-1}$) 218 (−22), 233 (−96), 257 (+19), 287 (+79), 317 (−3), 333 (+5), 394 (+19), 445 (−24), 517 (+3).

Δ-**FeNCCN2**

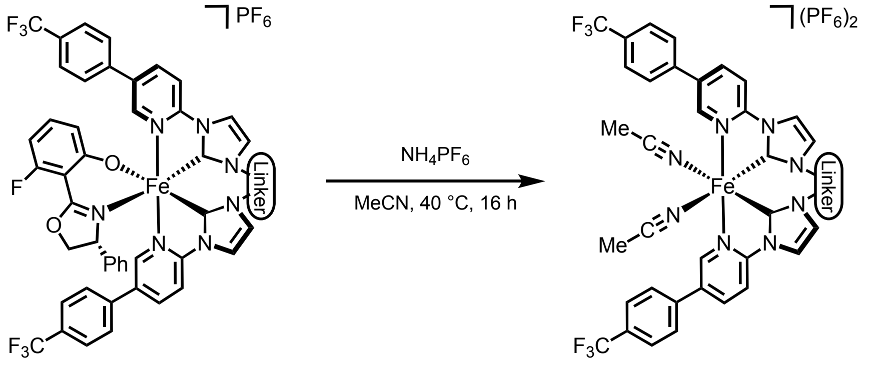

Δ-(*R*)-**FeAux2** Δ-**FeNCCN2**

Following the general procedure [**E**], Δ-**FeNCCN2** (56.4 mg, 43.1 µmol, 97%) was obtained as a red solid from the corresponding complex Δ-(*R*)-**FeAux2** (59.4 mg, 44.0 µmol). The spectroscopic data of the enantiopure complex were in accordance with *rac*-**FeNCCN2**.

CD: (MeCN, 0.25 mᴍ): λ, nm (Δε, $M^{-1}cm^{-1}$) 218 (+26), 233 (+120), 257 (−23), 287 (−100), 317 (+2), 333 (−8), 394 (−26), 445 (+28), 517 (−4).

Λ-**FeNCCN3**

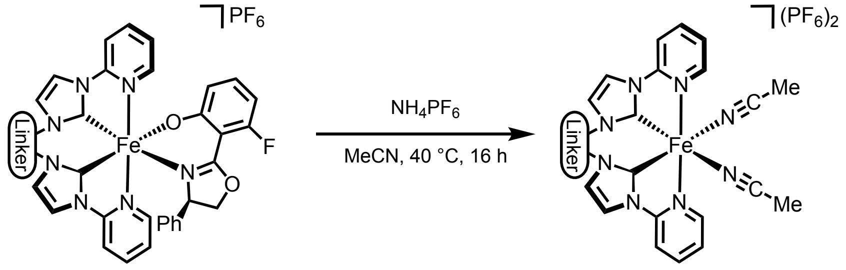

Λ-(*R*)-**FeAux3** Λ-**FeNCCN3**

Following the general procedure [**E**], Λ-**FeNCCN3** (40.4 mg, 37.2 µmol, 93%) was obtained as a red solid from the corresponding complex Λ-(*R*)-**FeAux3** (45.0 mg, 43.0 µmol). The spectroscopic data of the enantiopure complex were in accordance with *rac*-**FeNCCN3**.

CD: (MeCN, 0.25 mM): λ, nm (Δε, $M^{-1}cm^{-1}$) 234 (+111), 256 (−126), 276 (+60), 298 (−8), 322 (+5), 348 (+2), 381 (+15), 427 (−26), 487 (+5).

Δ-**FeNCCN3**

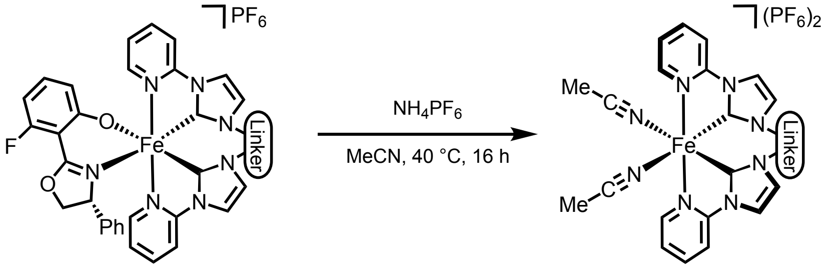

Δ-(*R*)-**FeAux3** Δ-**FeNCCN3**

Following the general procedure [**E**], Δ-**FeNCCN3** (44.4 mg, 43.5 µmol, 93%) was obtained as a red solid from the corresponding complex Δ-(*R*)-**FeAux3** (49.0 mg, 47.0 µmol). The spectroscopic data of the enantiopure complex were in accordance with *rac*-**FeNCCN3**.

CD: (MeCN, 0.25 mM): λ, nm (Δε, $M^{-1}cm^{-1}$) 234 (−99), 256 (+120), 276 (−59), 298 (+6), 322 (−8), 348 (−4), 381 (−15), 427 (+25), 487 (−6).

6. Determination of Enantiomeric Excess

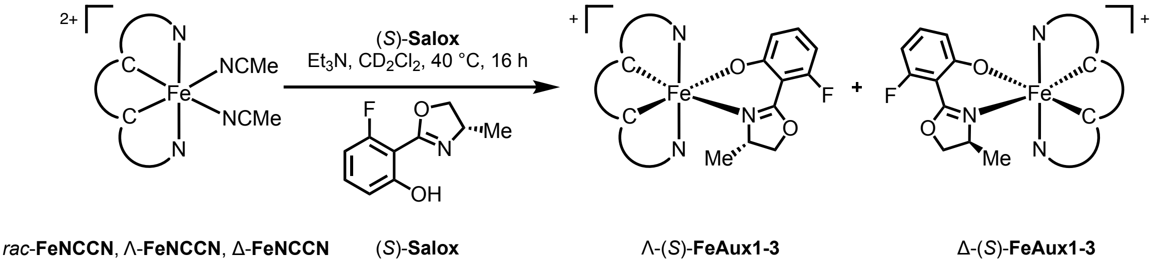

Following a procedure from the literature.[9] The iron complexes (1.00 eq) and the auxiliary (*S*)-**Salox** (4.00 eq) were dissolved in CD_2Cl_2 (0.004 M based on the iron complex), followed by the addition of Et_3N (8.00 eq). The mixture was placed in an NMR tube equipped with a magnetic stirring bar and stirred at room temperature for 16 h. A color change could be observed that correlated with the corresponding auxiliary complexes. The sample was then directly subjected to ^{19}F-NMR with 1000 scans, showing the diastereomeric ratio between Λ-(*S*)-**FeAux1-3** and Δ-(*S*)-**FeAux1-3.**

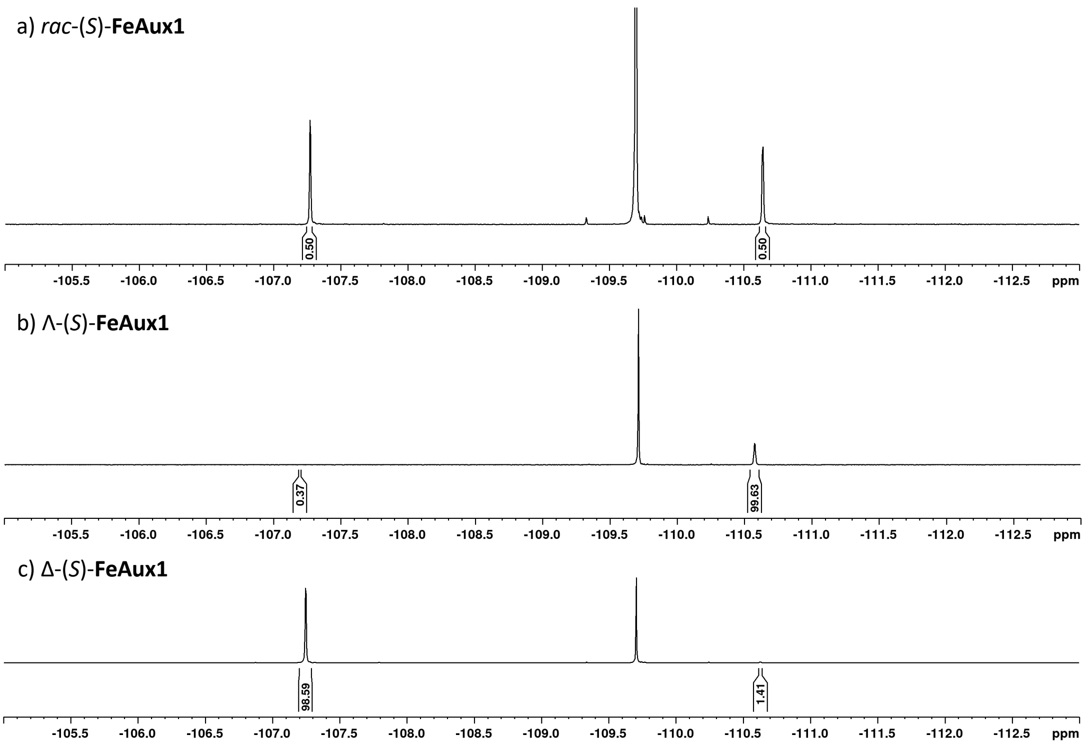

Figure S3: Enlarged excerpt of the ^{19}F-NMR spectra (282 MHz, CD_2Cl_2, 25 °C) with 1000 scans of the auxiliary complexes from **FeNCCN1** after recoordination of the auxiliary (*S*)-**Salox**.

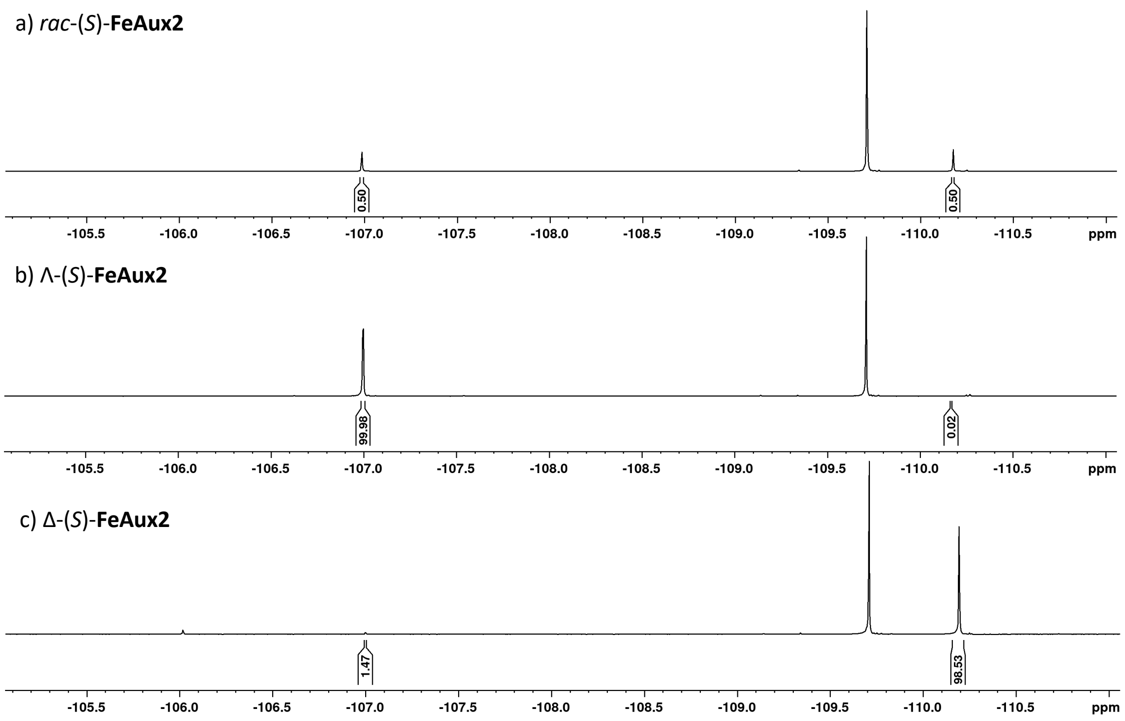

Figure S4: Enlarged excerpt of the ^{19}F-NMR spectra (282 MHz, CD_2Cl_2, 25 °C) with 1000 scans of the auxiliary complexes from **FeNCCN2** after recoordination of the auxiliary (*S*)-**Salox**.

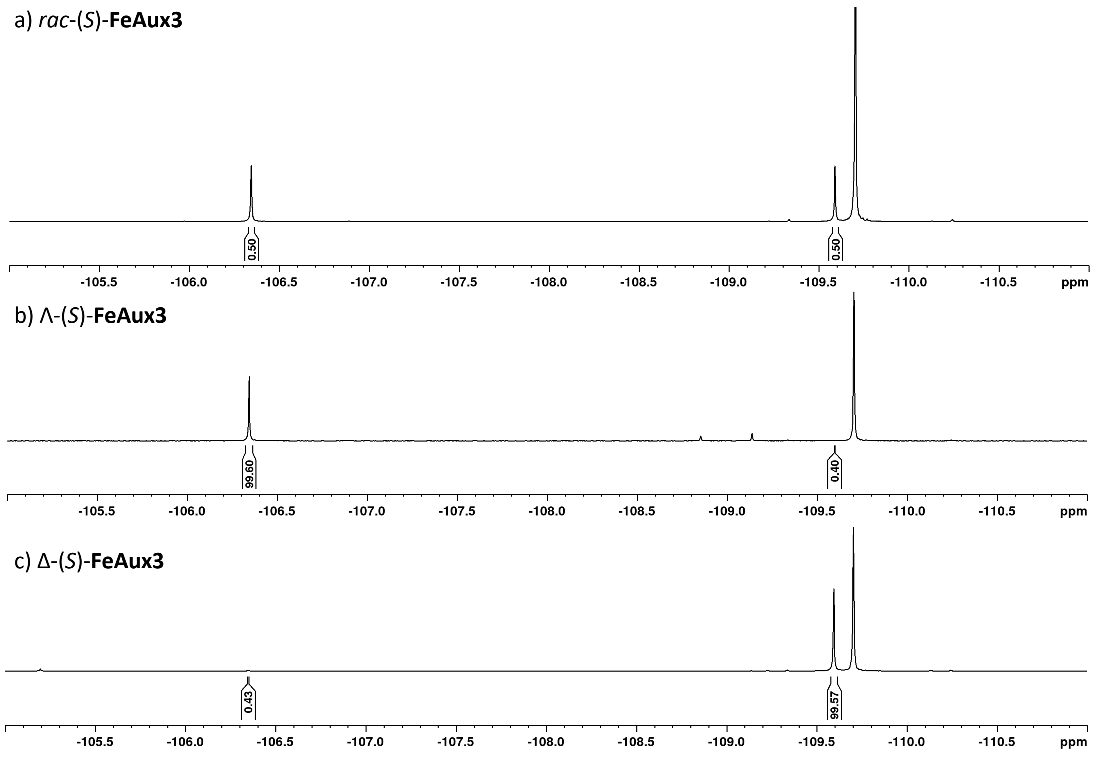

Figure S5: Enlarged excerpt of the ^{19}F-NMR spectra (282 MHz, CD_2Cl_2, 25 °C) with 1000 scans of the auxiliary complexes from **FeNCCN3** after recoordination of the auxiliary (*S*)-**Salox**.

7. Stability Experiments

Procedure:

A sample of the racemic complex in the corresponding solvent (CD_3CN or CD_2Cl_2) was prepared in an NMR tube and examined by ^{1}H-NMR after various incubation times. The samples were stored at room temperature under air. Figure S6-8 shows the overlayed spectra in CD_3CN, while the measured spectra in CD_2Cl_2 are shown in Figure S9-11.

Results:

The racemic complexes *rac*-**FeNCCN1-3** exhibit high stability in CD_3CN with no indications of decomposition after 21 days (Figure S6d) and even after 28 days of storage at room temperature under air (Figure S7e + S8d), as shown by comparisons of the ^{1}H-NMR spectra with the original sample (Figure S6-8a). When comparing the stability of the complexes in CD_2Cl_2, differences began to emerge immediately after 2 days, when slight decomposition was observed for *rac*-**FeNCCN2** (Figure S10b), while the other complexes, *rac*-**FeNCCN1** and *rac*-**FeNCCN3**, remained intact for up to one week (Figure S9+S11).

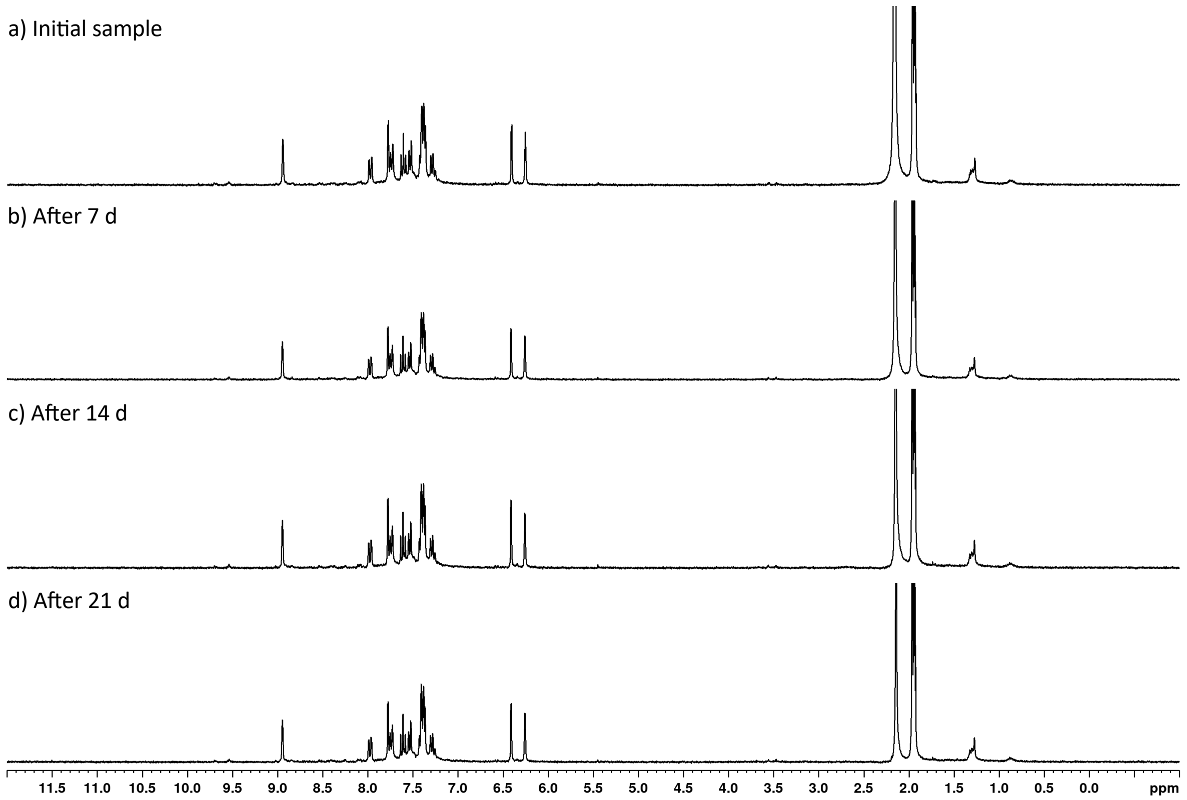

Figure S6: ^{1}H-NMR spectra (300 MHz, CD_3CN, 25 °C) of *rac*-**FeNCCN1** after different periods of storage in CD_3CN.

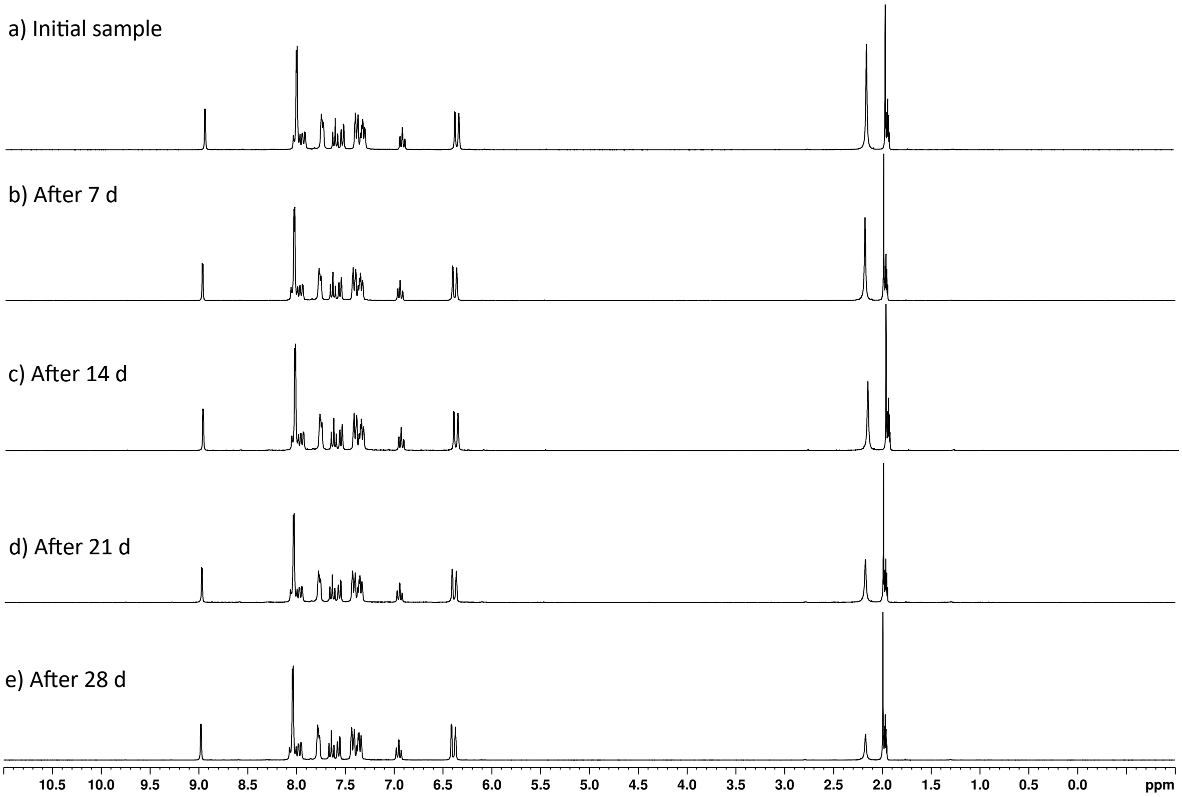

Figure S7: ^{1}H-NMR spectra (300 MHz, CD_3CN, 25 °C) of *rac*-**FeNCCN2** after different periods of storage in CD_3CN.

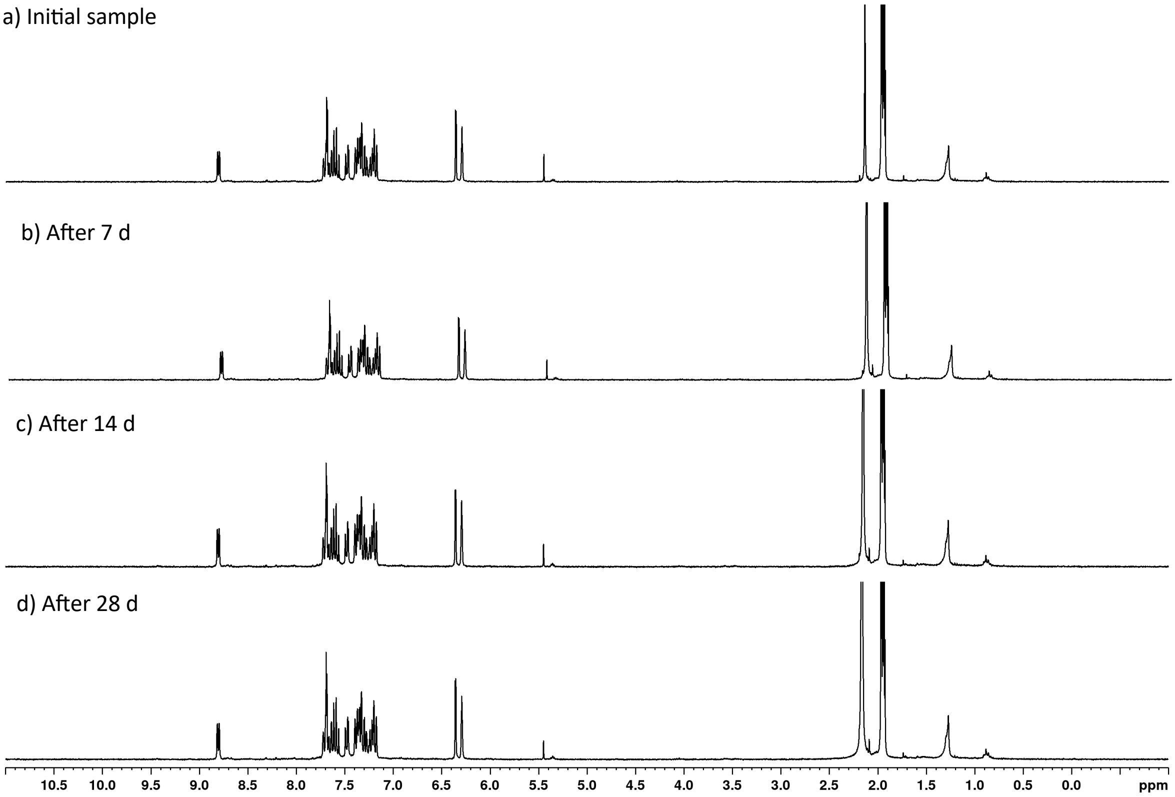

Figure S8: ^{1}H-NMR spectra (300 MHz, CD_3CN, 25 °C) of *rac*-**FeNCCN3** after different periods of storage in CD_3CN.

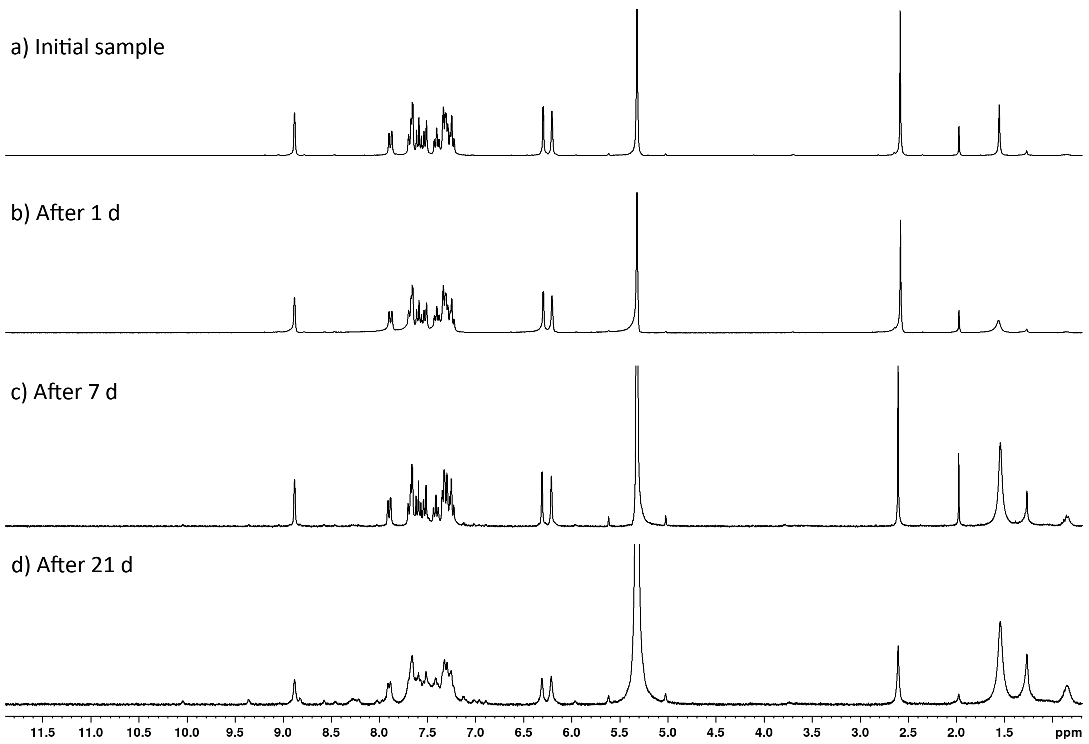

Figure S9: ^{1}H-NMR spectra (300 MHz, CD_2Cl_2, 25 °C) of *rac*-**FeNCCN1** after different periods of storage in CD_2Cl_2.

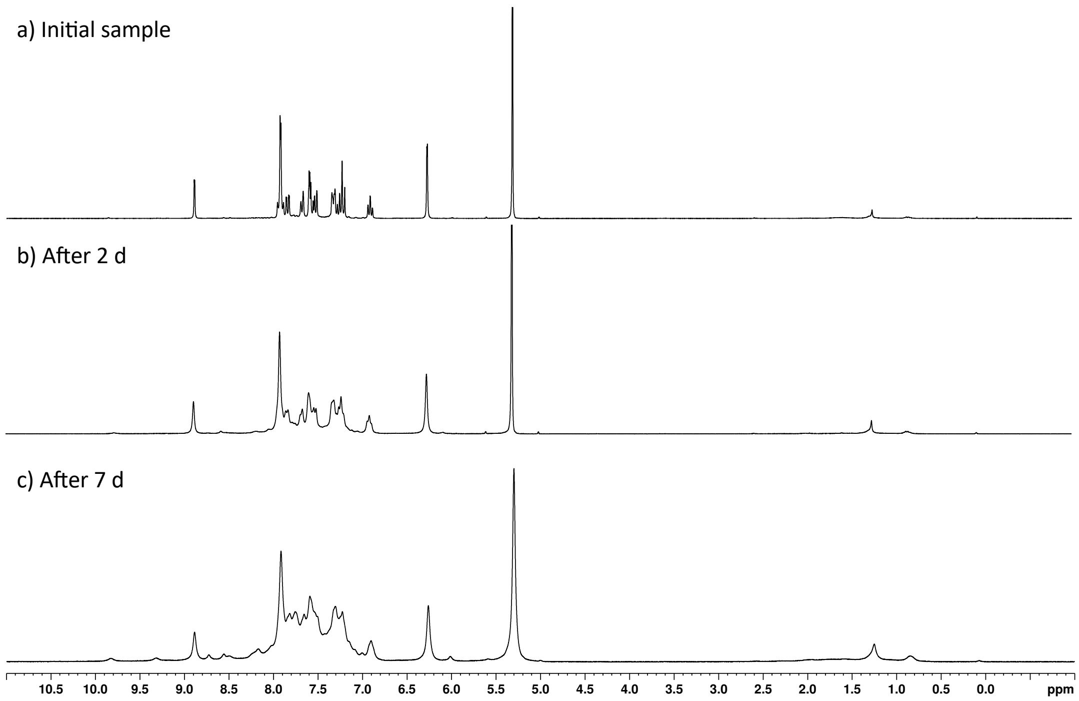

Figure S10: ^{1}H-NMR spectra (300 MHz, CD_2Cl_2, 25 °C) of *rac*-**FeNCCN2** after different periods of storage in CD_2Cl_2.

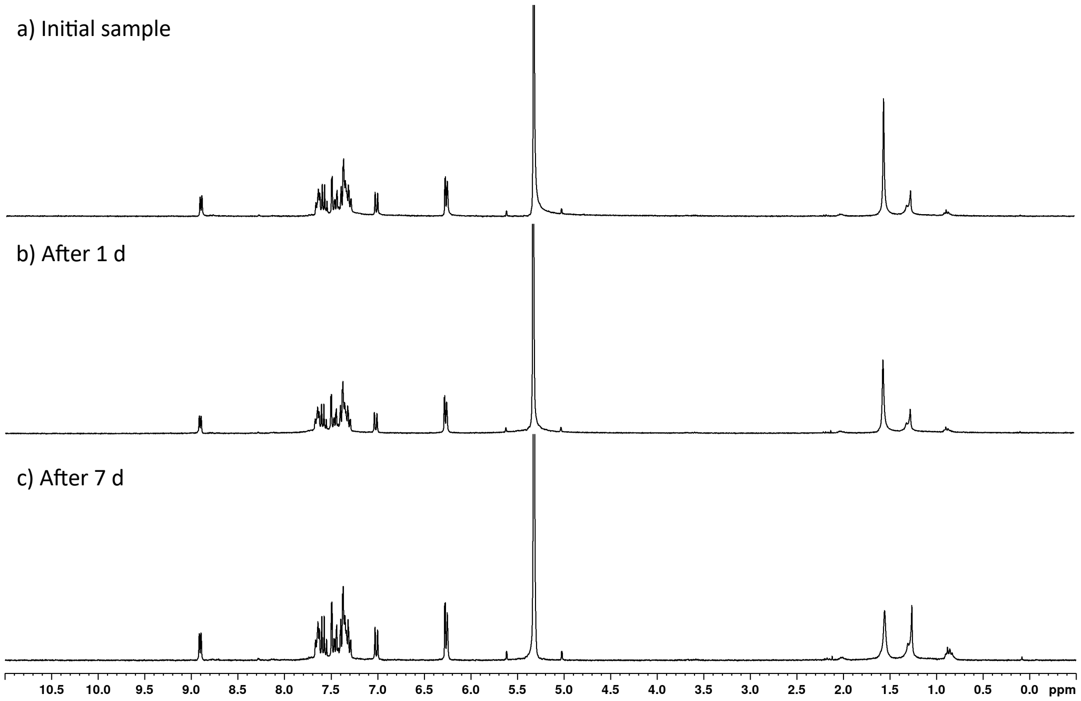

Figure S11: ^{1}H-NMR spectra (300 MHz, CD_2Cl_2, 25 °C) of *rac*-**FeNCCN3** after different periods of storage in CD_2Cl_2.

General Procedure F: Configurational stability of the nonracemic chiral-at-iron complexes

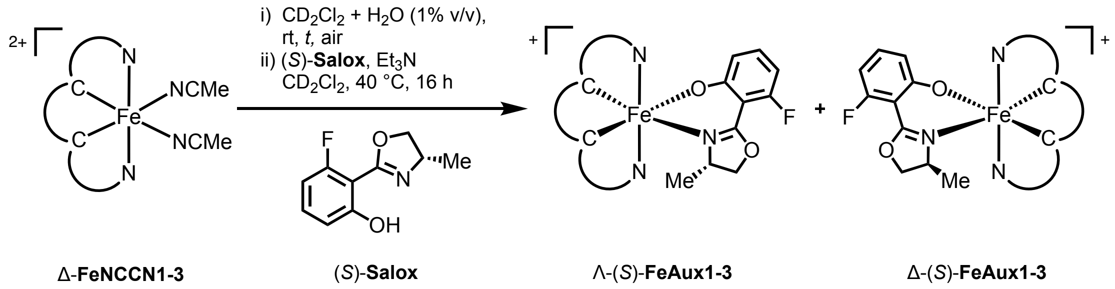

Following a modified procedure from the literature.[7] The single enantiomer of the iron complexes (1.00 eq) was dissolved in CD_2Cl_2 (0.004 M based on the iron complex) with H_2O (1% v/v), placed in an NMR tube equipped with a magnetic stirring bar, and stirred at room temperature for the indicated time (See figures S12-S14). Accordingly, the auxiliary (*S*)-**Salox** (2.00 eq) was added along with Et_3N (3.00 eq), and the mixture was stirred at 40 °C for 16 h. Afterward, the sample was directly subjected to ^{19}F-NMR with 1000 scans, showing the diastereomeric ratio between Λ-(*S*)-**FeAux** and Δ-(*S*)-**FeAux**. Figure S12-14 shows the stacked ^{19}F-NMR spectra with the racemic sample as orientation. The summarized experimental data can be obtained from Table 1 of the main manuscript.

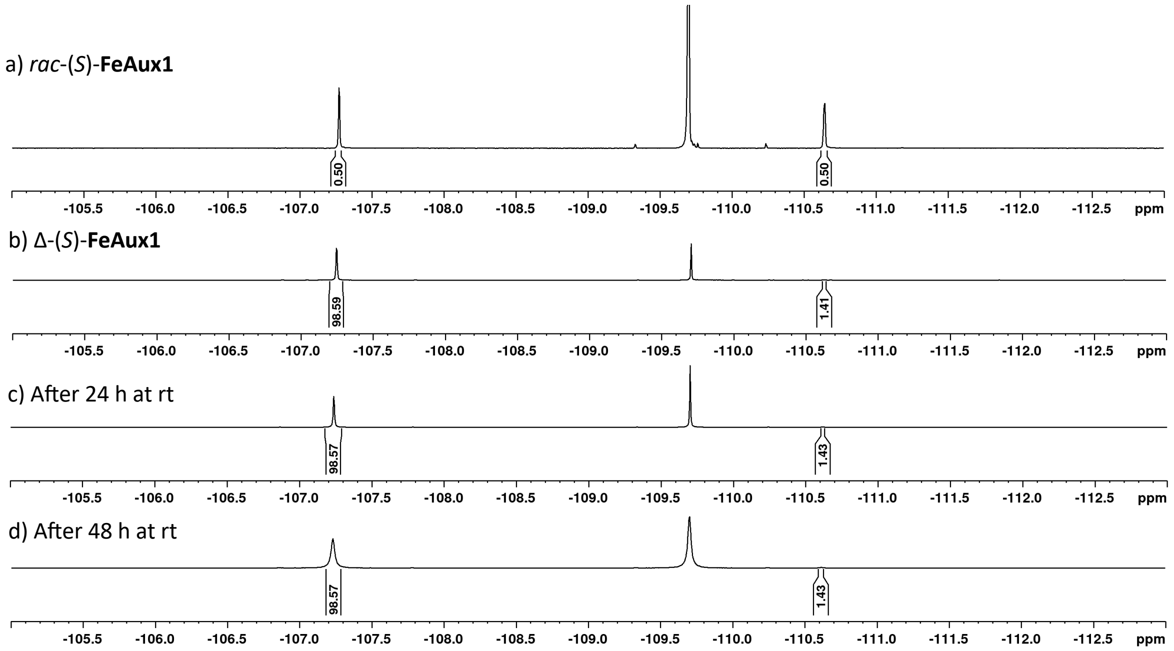

Figure S12: Enlarged excerpt of the ^{19}F-NMR spectra (282 MHz, CD_2Cl_2, 25 °C) with 1000 scans of the auxiliary complexes after treatment of Δ-**FeNCCN1** under the conditions of [**F**] and subsequent recoordination of the auxiliary (*S*)-**Salox**.

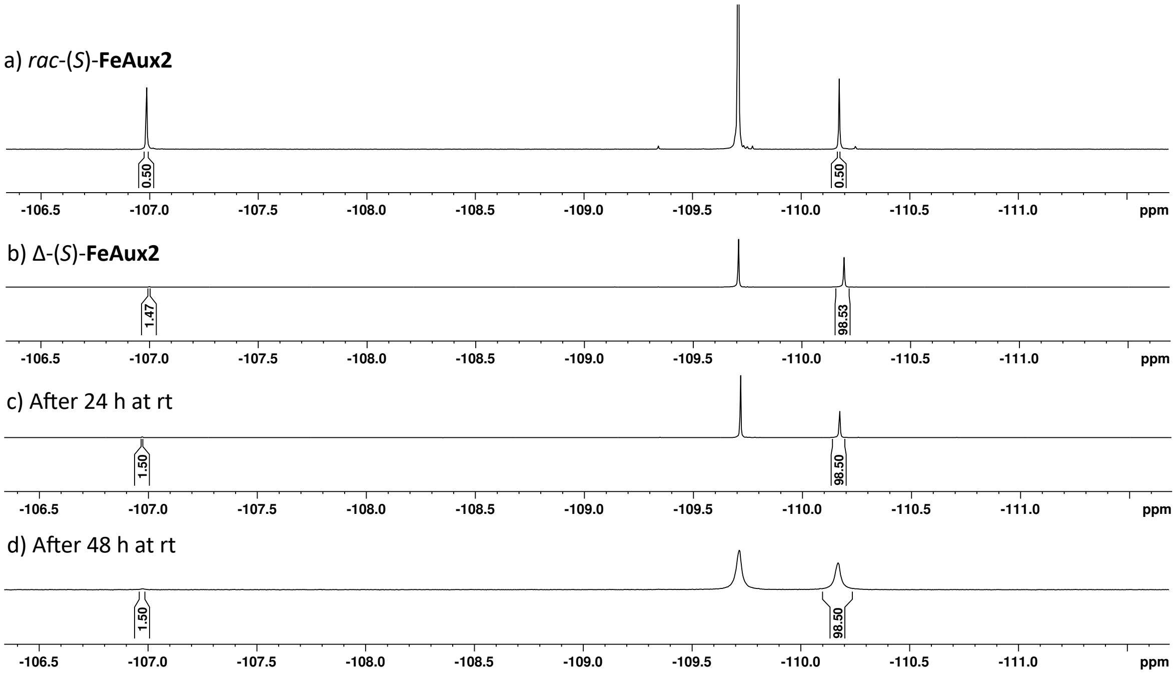

Figure S13: Enlarged excerpt of the ^{19}F-NMR spectra (282 MHz, CD_2Cl_2, 25 °C) with 1000 scans of the auxiliary complexes after treatment of Δ-**FeNCCN2** under the conditions of [**F**] and subsequent recoordination of the auxiliary (*S*)-**Salox**.

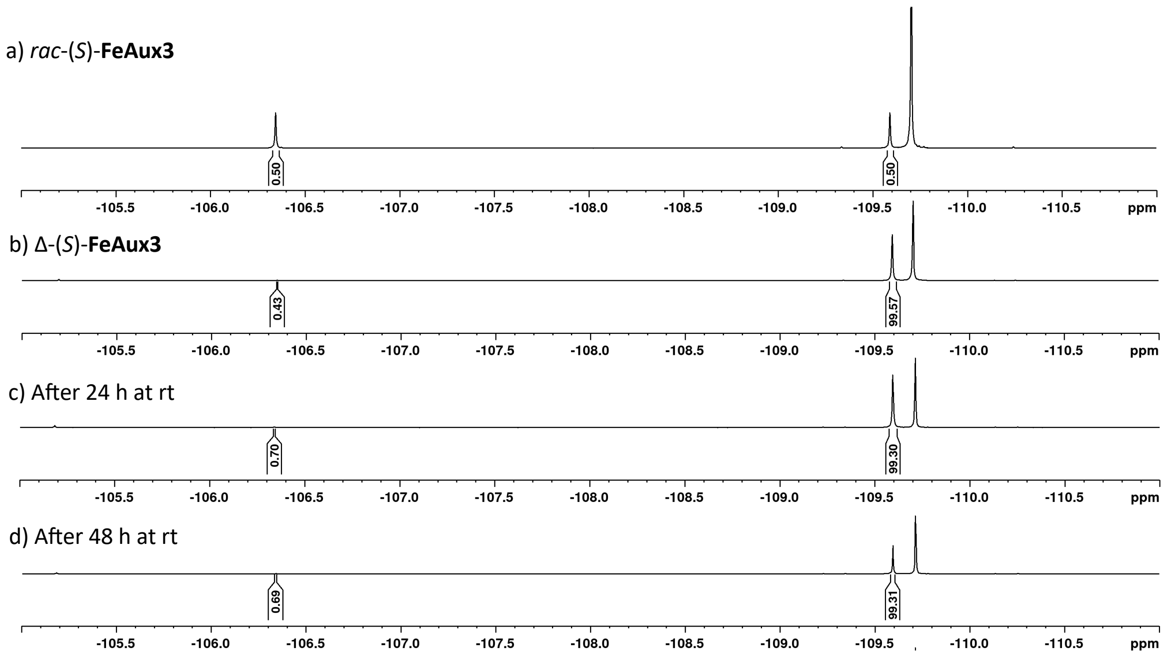

Figure S14: Enlarged excerpt of the ^{19}F-NMR spectra (282 MHz, CD_2Cl_2, 25 °C) with 1000 scans of the auxiliary complexes after treatment of Δ-**FeNCCN3** under the conditions of [**F**] and subsequent recoordination of the auxiliary (*S*)-**Salox**.

8. Catalysis

Intramolecular Cannizzaro Reaction

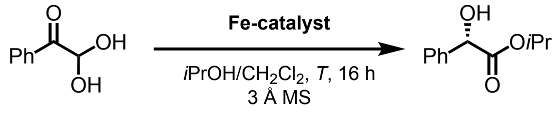

11 (S)-**12**

Following a slightly modified procedure from the literature.[8] The iron catalyst (5 mol%), molecular sieve (3 Å, 5 mg per 0.01 mmol substrate), and phenylglyoxal (**11**) (7.60 mg, 0.05 mmol, 1.00 eq) were suspended in dry and degassed CH_2Cl_2 (0.05 M) under an atmosphere of nitrogen in a Schlenk tube. Then *i*PrOH (37.5 µL, 0.50 mmol, 10.0 eq) was added, and the mixture was stirred for 16 h at the indicated temperature. Then, *n*-pentane (5 mL) was added, and the reaction mixture was filtered over a short celite plug to remove the catalyst and rinsed with *n*-pentane/CH_2Cl_2 (4:1). The solvent was removed under reduced pressure, and the yield was determined by ^{1}H-NMR analysis of the crude product with TMB as internal standard. The enantiomeric excess of the crude product was determined by HPLC analysis on a chiral stationary phase. The spectroscopic data are in accordance with the literature.[8] The absolute configuration of the product was determined by comparison of the HPLC traces with those reported in the literature.[8]

TLC: R_f = 0.41 (*n*-pentane/EtOAc 3:1).

^{1}H-NMR: (300 MHz, $CDCl_3$) δ (ppm) = 7.44–7.30 (m, 5H), 5.14–5.01 (m, 2H), 3.47 (d, *J* = 5.8 Hz, 1H), 1.28 (d, *J* = 6.3 Hz, 3H), 1.11 (d, *J* = 6.2 Hz, 3H)

HPLC: Daicel Chiralcel® OD-H column, 250 x 4.6 mm, absorbance at 210 nm, *n*-hexane/iPrOH 95:5, isocratic flow, flow rate 1.0 mL/min, 25 °C, t_r (minor) = 7.67 min, t_r (major) = 14.72 min.

C(sp^3)-H-amidation of benzoyloxyurea derivatives

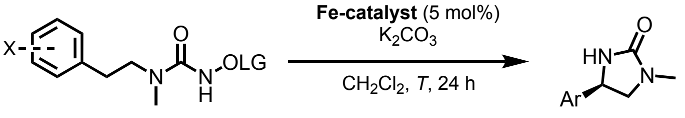

Following a slightly modified procedure from the literature.[10] The iron catalyst (5 mol%), K_2CO_3 (0.10 mmol, 3.00 eq) and the urea substrate **13a-e** (0.03 mmol, 1.00 eq) were placed in a Schlenk tube under an atmosphere of nitrogen. Dry and degassed CH_2Cl_2 (0.1 M) was added, and the reaction mixture was stirred for 24 h at the indicated temperature. Then, *n*-pentane (5 mL) was added, and the reaction mixture was filtered over a short celite plug to remove the catalyst and rinsed with *n*-pentane/CH_2Cl_2 (4:1). The solvent was removed under reduced pressure, and the yield was determined by ^{1}H-NMR analysis of the crude product with TMB as internal standard. The enantiomeric excess of the crude product was determined by HPLC analysis on a chiral stationary phase. The spectroscopic data are in accordance with the literature.[1] The absolute configuration of the product was determined by comparison of the HPLC traces with those reported in the literature.[11]

Analytical data for: **14a**:

TLC: R_f = 0.17 (*n*-pentane/EtOAc 2:1).

^{1}H-NMR: (300 MHz, $CDCl_3$) δ (ppm) = 7.38–7.28 (m, 5H), 5.41 (s, 1H), 4.76 (t, *J* = 8.1 Hz, 1H), 3.79 (t, 1H), 3.24 (t, *J* = 8.0 Hz, 1H), 2.83 (s, 3H).

HPLC: Daicel Chiralcel® IA column, 250 x 4.6 mm, absorbance at 220 nm, *n*-hexane/*i*PrOH 95:5, isocratic flow, flow rate 1.0 mL/min, 30 °C, t_r (minor) = 28.60 min, t_r (major) = 22.86 min.

Analytical data for: **14c**:

^{1}H-NMR: (300 MHz, $CDCl_3$) δ (ppm) = 7.45–7.29 (m, 4H), 4.95–4.80 (m, 2H), 3.91 (t, *J* = 8.8 Hz, 1H), 3.35 (dd, *J* = 8.7, 7.4 Hz, 1H), 2.97 (s, 3H), 2.51 (s, 3H).

HPLC: Daicel Chiralcel® IA column, 250 x 4.6 mm, absorbance at 220 nm, *n*-hexane/*i*PrOH 95:5, isocratic flow, flow rate 1.0 mL/min, 40 °C, t_r (minor) = 33.85 min, t_r (major) = 28.09 min.

Analytical data for: **14d**:

^{1}H-NMR: (300 MHz, $CDCl_3$) δ (ppm) = 7.40–7.17 (m, 4H), 4.89–4.73 (m, 2H), 3.86 (t, *J* = 8.7 Hz, 1H), 3.29 (t, *J* = 7.3 Hz, 1H), 2.90 (s, 3H), 2.44 (s, 3H).

HPLC: Daicel Chiralcel® IA column, 250 x 4.6 mm, absorbance at 220 nm, *n*-hexane/*i*PrOH 95:5, isocratic flow, flow rate 1.0 mL/min, 25 °C, t_r (minor) = 28.17 min, t_r (major) = 21.87 min.

Analytical data for: **14e**:

^{1}H-NMR: (300 MHz, $CDCl_3$) δ (ppm) = 7.55–7.46 (m, 1H), 7.31 – 7.10 (m, 3H), 4.98 (t, *J* = 7.1 Hz, 1H), 4.81 (s, 1H), 3.85 (t, *J* = 8.8 Hz, 1H), 3.12 (t, *J* = 7.2 Hz, 1H), 2.80 (s, 3H), 2.32 (s, 3H).

HPLC: Daicel Chiralcel® IA column, 250 x 4.6 mm, absorbance at 220 nm, *n*-hexane/*i*PrOH 95:5, isocratic flow, flow rate 1.0 mL/min, 25 °C, t_r (minor) = 33.51 min, t_r (major) = 26.38 min.

Hetero-Diels–Alder Reaction

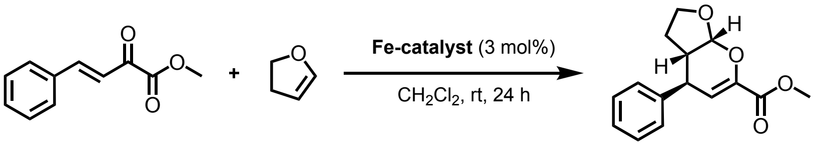

15 **16** **17**

Following a slightly modified procedure from the literature.[12] The iron catalyst (3 mol%) and ketoester **15** (0.05 mmol, 1.00 eq) were dissolved in dry and degassed CH_2Cl_2 (0.05 M) under an atmosphere of nitrogen in a Schlenk tube. Then, dihydrofuran **16** (0.80 mmol, 15.0 eq) was added, and the mixture was stirred at room temperature for 24 h. Then, *n*-pentane (5 mL) was added, and the reaction mixture was filtered over a short celite plug to remove the catalyst and rinsed with *n*-pentane/CH_2Cl_2 (4:1). The solvent was removed under reduced pressure, and the yield was determined by ^{1}H-NMR analysis of the crude product with TMB as internal standard. The enantiomeric excess of the crude product was determined by HPLC analysis on a chiral stationary phase. The spectroscopic data are in accordance with the literature.[12] The absolute configuration of the product was determined by comparison of the HPLC traces with those reported in the literature.[12]

TLC: R_f = 0.32 (*n*-pentane/EtOAc 5:1).

^{1}H-NMR: (300 MHz, $CDCl_3$) δ (ppm) = 7.37–7.20 (m, 5H), 6.20 (dd, *J* = 1.3 Hz, 1H), 5.64 (d, *J* = 3.5 Hz, 1H), 4.20–4.13 (m, 2H), 3.90–3.86 (m, 1H), 3.83 (s, 3H), 2.71–2.59 (m, 1H), 1.71 (tt, *J* = 12.3 Hz, 1H), 1.37–1.31 (m, 1H).

HPLC: Daicel Chiralcel® IG column, 250 x 4.6 mm, absorbance at 254 nm, *n*-hexane/*i*PrOH 95:5, isocratic flow, flow rate 1.0 mL/min, 25 °C, t_r (minor) = 32.25 min, t_r (major) = 38.36 min.

9. Synthesis of the Chiral Auxiliaries and Pyridine 6

General Procedure G: Chiral Auxiliary (*R*)-Salox and (*S*)-Salox

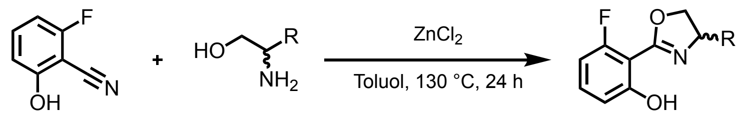

18 **19** R = Ph, **20** R = Me (*R*/*S*)-**Salox**

Following a literature known procedure.[13] $ZnCl_2$ (62.9 mg, 0.46 mmol, 0.05 eq) was heated under vacuo at 350 °C for 30 min. After cooling to room temperature, 2-hydroxybenzonitrile (**18**, 1.00 eq) and (*R*/*S*)-amino alcohol (1.50 eq) were added with toluene (0.32 M based on the nitrile) under an atmosphere of nitrogen and the reaction mixture was stirred at 130 °C for 24 h. After cooling to room temperature, the solvent was removed under reduced pressure, and the crude product was purified by flash column chromatography (silica gel, *n*-hexane/EtOAc, 99:1 → 80:20) to obtain the auxiliaries (*R*)-**Salox** or (*S*)-**Salox**.

(*R*)-3-fluoro-2-(4-phenyl-4,5-dihydrooxazol-2-yl)phenol ((*R*)-Salox)

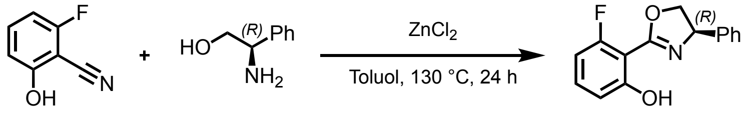

18 **19** (*R*)-**Salox**

Following the general procedure [**G**], (*R*)-**Salox** (1.32 g, 5.77 mmol, 59%) was obtained as a colorless solid starting from the nitrile **18** (1.20 g, 8.75 mmol). The spectroscopic data are in accordance with the literature.[14]

TLC: R_f = 0.43 (*n*-pentane/EtOAc 15:1).

^{1}H-NMR: (300 MHz, $CDCl_3$) δ (ppm) = 13.09 (s, 1H), 7.45 – 7.23 (m, 6H), 6.85 (d, *J* = 8.5 Hz, 1H), 6.63 (ddd, *J* = 11.1, 8.3, 1.0 Hz, 1H), 5.42 (dd, *J* = 10.2, 8.4 Hz, 1H), 4.87 (dd, *J* = 10.3, 8.6 Hz, 1H), 4.32 (t, *J* = 8.5 Hz, 1H).

^{19}F-NMR: (282 MHz, $CDCl_3$) δ (ppm) = –108.75 (s, 1F).

(*S*)-3-fluoro-2-(4-methyl-4,5-dihydrooxazol-2-yl)phenol ((*S*)-Salox)

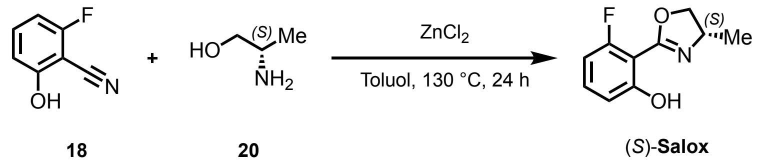

Following the general procedure [**G**], (*S*)-**Salox** (0.58 g, 2.99 mmol, 82%) was obtained as a colorless solid starting from the nitrile **18** (0.50 g, 3.65 mmol).

TLC: R_f = 0.46 (*n*-pentane/EtOAc 10:1).

MP: 62 °C.

^{1}H-NMR: (300 MHz, $CDCl_3$) δ (ppm) = 13.18 (s, 1H), 7.34 – 7.20 (m, 1H), 6.80 (dt, *J* = 8.4, 1.0 Hz, 1H), 6.57 (ddd, *J* = 11.1, 8.3, 1.1 Hz, 1H), 4.56 (dd, *J* = 9.4, 8.2 Hz, 1H), 4.38 (ddt, *J* = 13.4, 9.2, 6.7 Hz, 1H), 4.01 (t, *J* = 7.9 Hz, 1H), 1.36 (d, *J* = 6.5 Hz, 3H).

^{13}C-NMR: (75 MHz, $CDCl_3$) δ (ppm) = 164.27, 164.23, 163.18, 161.83, 161.77, 159.77, 133.32, 133.17, 112.88, 112.84, 106.20, 105.91, 100.59, 100.42, 73.85, 73.84, 59.37, 21.47.

^{19}F-NMR: (282 MHz, $CDCl_3$) δ (ppm) = –109.23 (s, 1F).

HRMS: ESI(+); *m/z* calculated for $C_{10}H_{10}FNO_2H$ $[M+H]^+$: 196.07, found: 196.0768 $[M+H]^+$.

IR: ṽ (cm^{-1}) = 2965 (w), 2928 (w), 2550 (w), 1635 (w), 1607 (s), 1568 (w), 1479 (w), 1457 (m), 1447 (w), 1380 (w), 1348 (m), 1316 (m), 1257 (m), 1233 (s), 1161 (w), 1144 (w), 1116 (w), 1086 (w), 1072 (m), 1056 (w), 1020 (s), 949 (m), 894 (w), 863 (w), 840 (m), 793 (s), 747 (m), 707 (w), 680 (m), 582 (m), 514 (m), 460 (w), 422 (w).

2-bromo-5-(4-(trifluoromethyl)phenyl)pyridine (6)

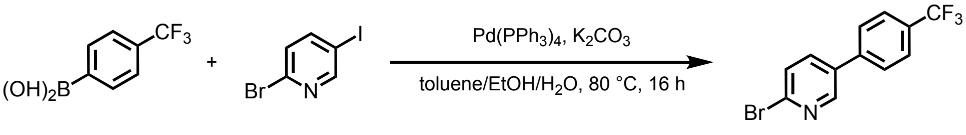

Following a modified procedure from the literature.[5] The arylboronic acid **21** (1.00 g, 5.28 mmol, 1.00 eq), K_2CO_3 (2.19 g, 15.8 mmol, 3.00 eq), $Pd(PPh_3)_4$ (30.5 mg, 0.26 mmol, 0.05 eq), and the pyridine **22** (1.50 g, 5.28 mmol, 1.00 eq) were placed under nitrogen atmosphere in a SCHLENK tube. Toluene/EtOH/H_2O (3:1:1, 0.2 M based on the boronic acid) was added, and the reaction mixture was stirred at 80 °C for 16 h. After cooling to room temperature, the mixture was diluted with H_2O and extracted three times with EtOAc. The combined organic phases were then washed with brine and dried over $MgSO_4$. The solvent was removed under reduced pressure, and the crude product was purified by flash column chromatography (silica gel, *n*-hexane/EtOAc, 99:1 → 80:20) to obtain the coupled pyridine **6** (1.23 g, 4.07 mmol, 77%) as a colorless solid. The spectroscopic data are in accordance with the literature.[15]

TLC: R_f = 0.36 (*n*-pentane/EtOAc 15:1).

^{1}H-NMR: (300 MHz, $CDCl_3$) δ (ppm) = 8.60 (d, *J* = 2.6 Hz, 1H), 7.75 (dd, *J* = 8.4, 2.9 Hz, 3H), 7.66 (d, *J* = 8.2 Hz, 2H), 7.59 (d, *J* = 8.2 Hz, 1H).

^{19}F-NMR: (282 MHz, $CDCl_3$) δ (ppm) = –62.65 (s, 3F).

10. NMR Spectra

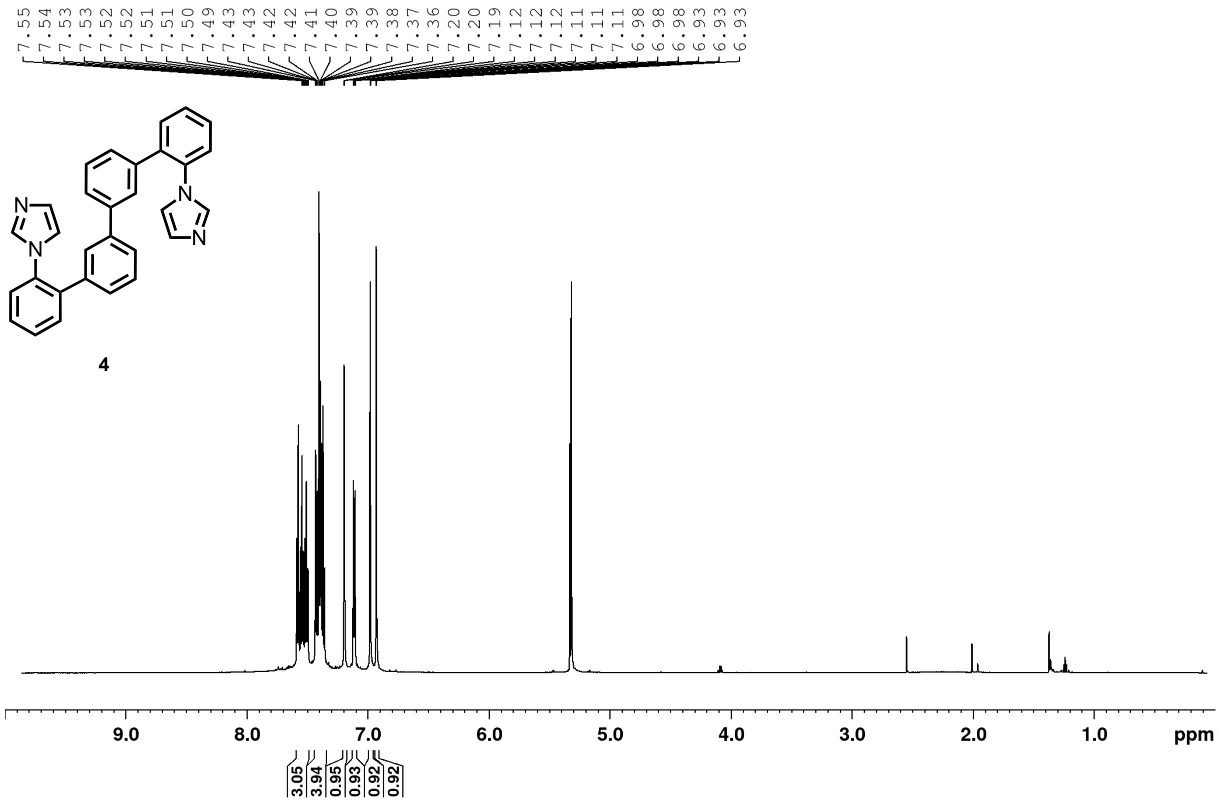

Figure S15: ^{1}H-NMR spectrum of **4** (600 MHz, CD_2Cl_2, 25 °C).

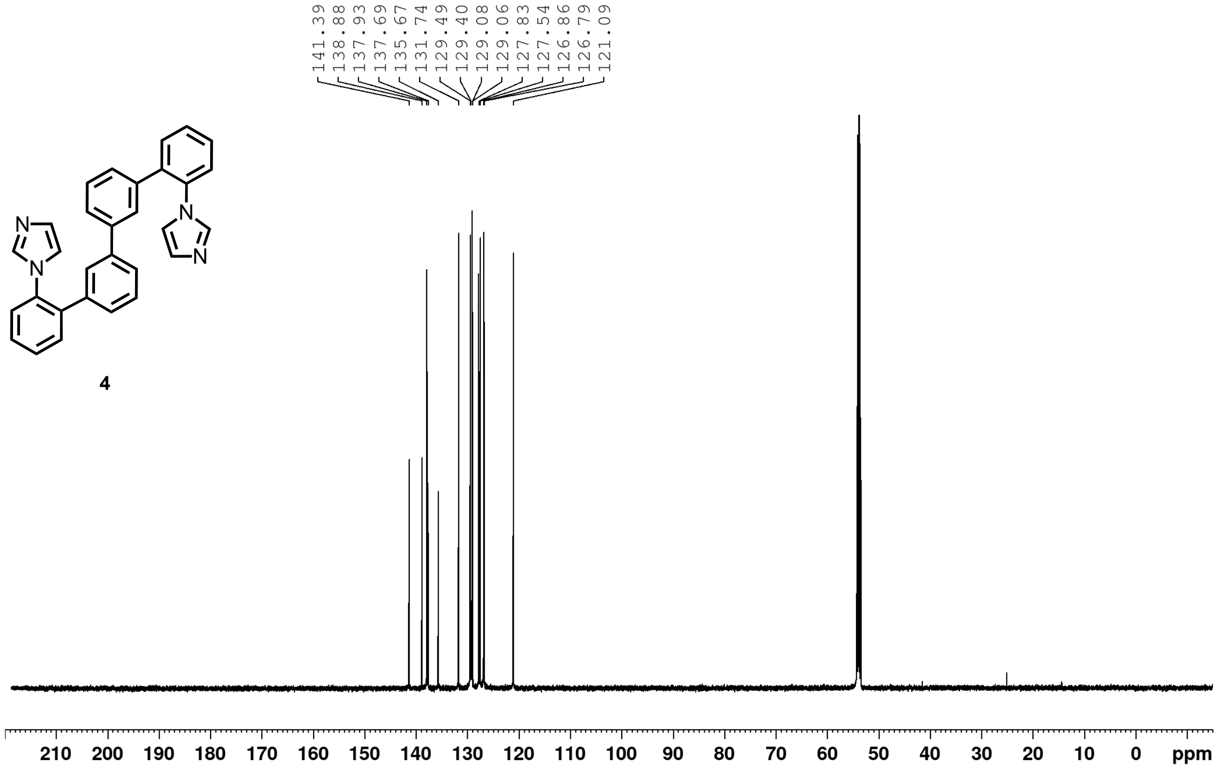

Figure S16: ^{13}C-NMR spectrum of **4** (151 MHz, CD_2Cl_2, 25 °C).

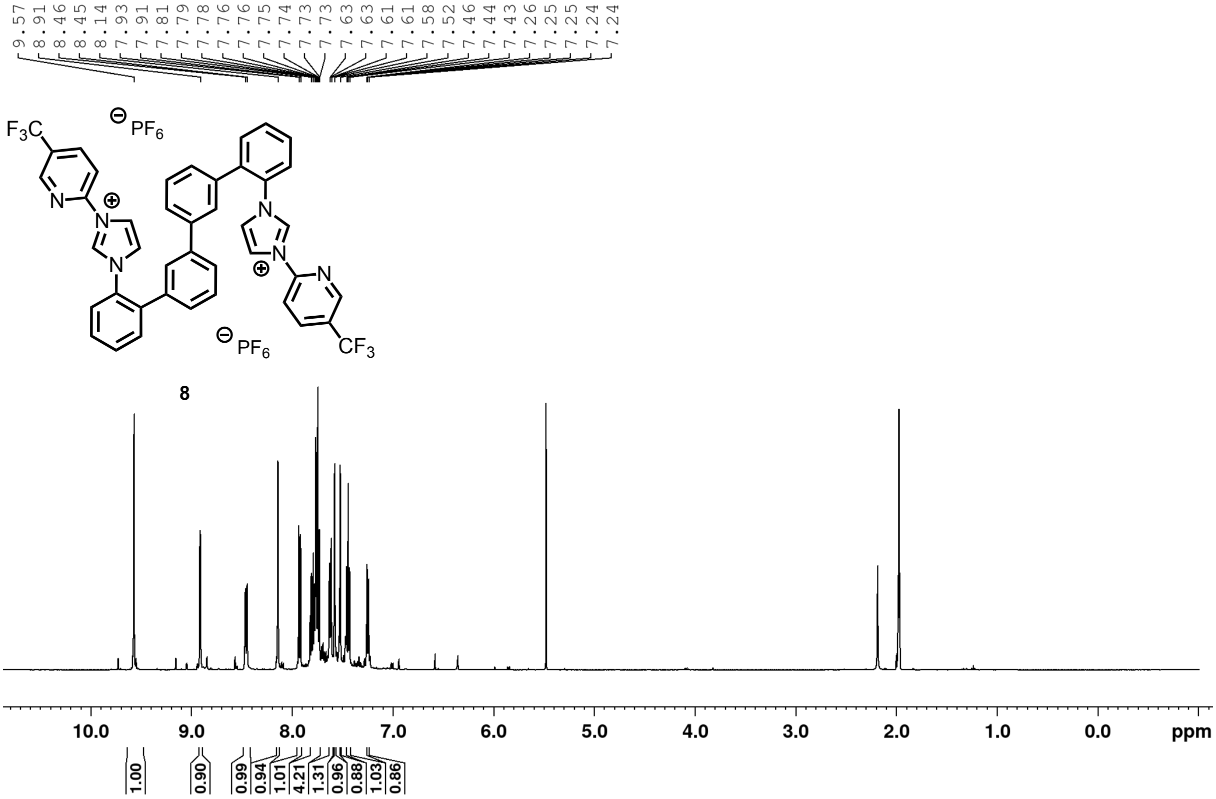

Figure S17: ^{1}H-NMR spectrum of **8** (500 MHz, CD_3CN, 25 °C).

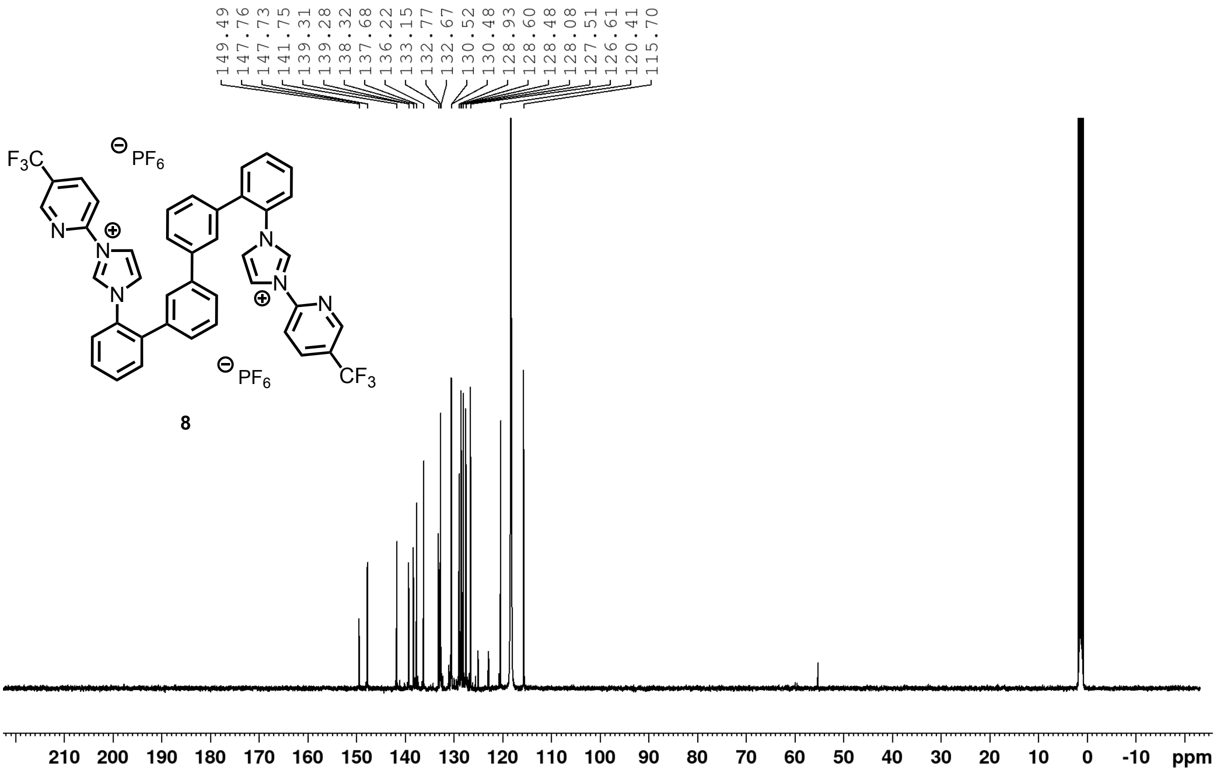

Figure S18: ^{13}C-NMR spectrum of **8** (126 MHz, CD_3CN, 25 °C).

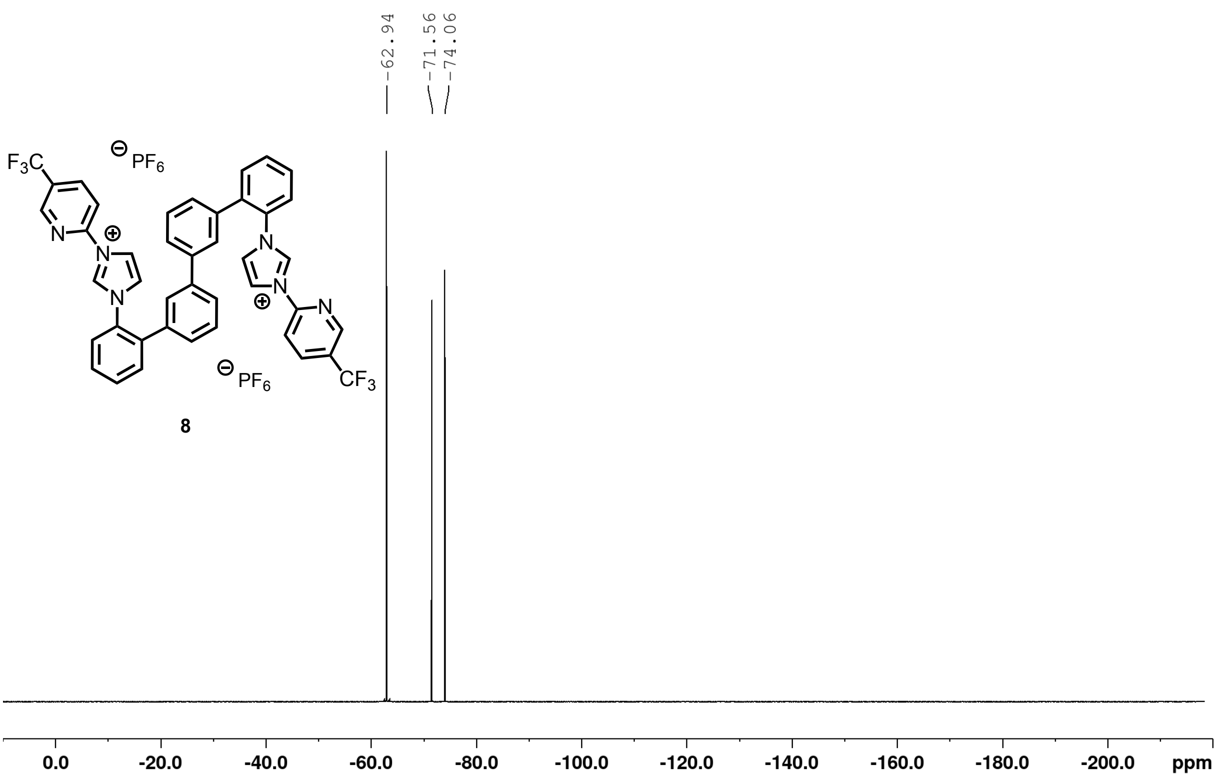

Figure S19: ^{19}F-NMR spectrum of **8** (282 MHz, $CDCl_3$, 25 °C).

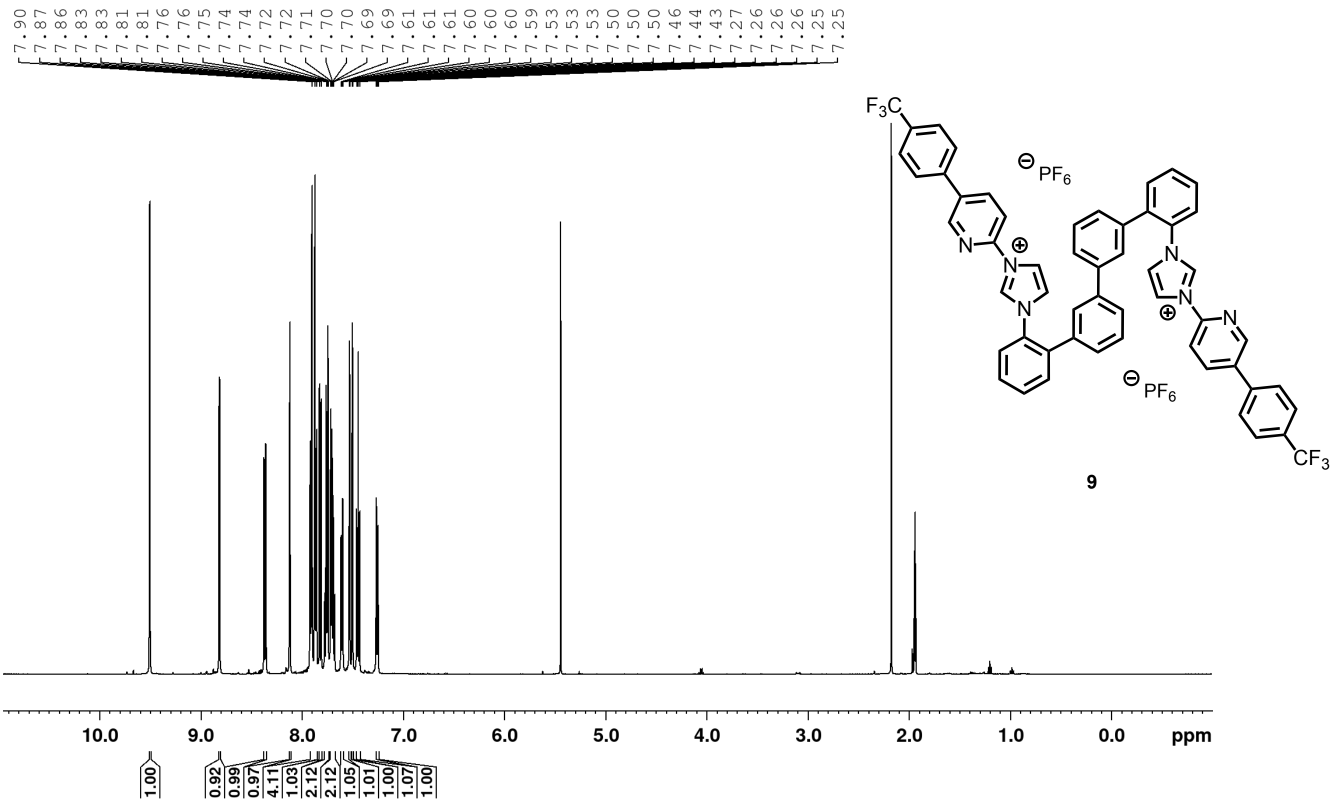

Figure S20: ^{1}H-NMR spectrum of **9** (500 MHz, CD_3CN, 25 °C).

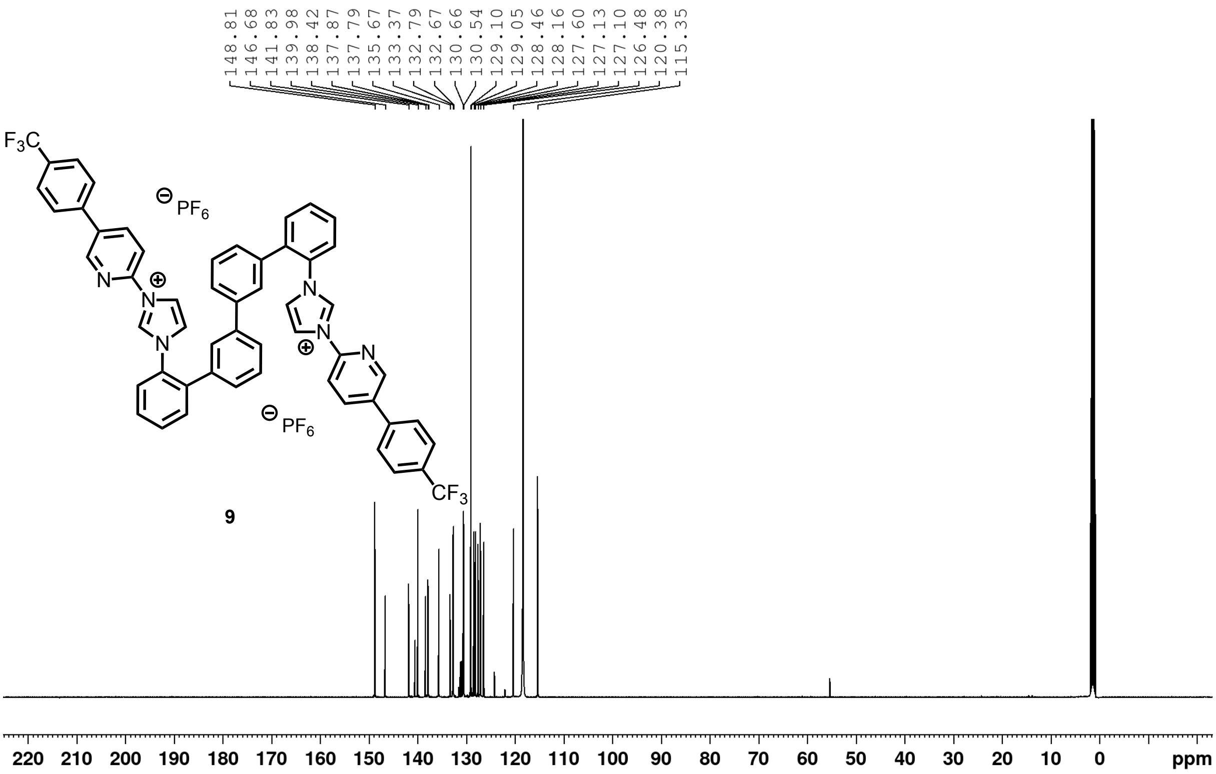

Figure S21: ^{13}C-NMR spectrum of **9** (126 MHz, CD_3CN, 25 °C).

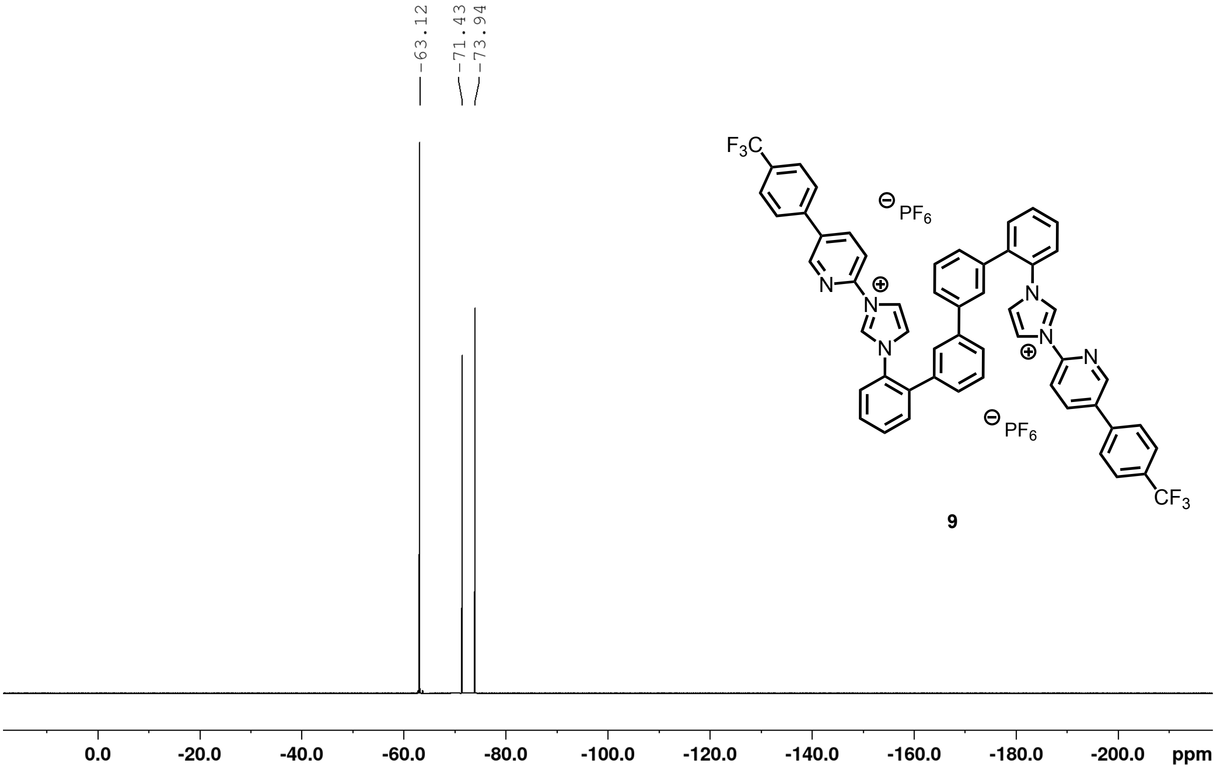

Figure S22: ^{19}F-NMR spectrum of **8** (282 MHz, $CDCl_3$, 25 °C).

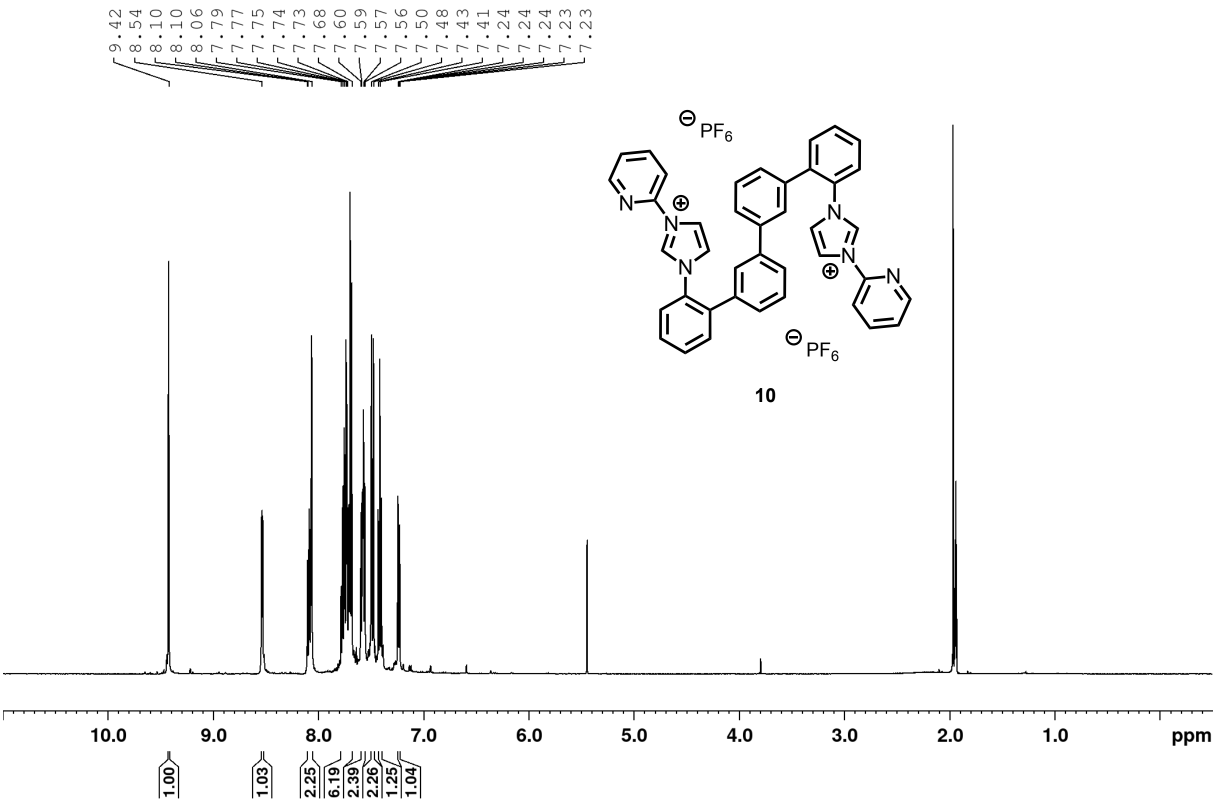

Figure S23: ^{1}H-NMR spectrum of **10** (500 MHz, CD_3CN, 25 °C).

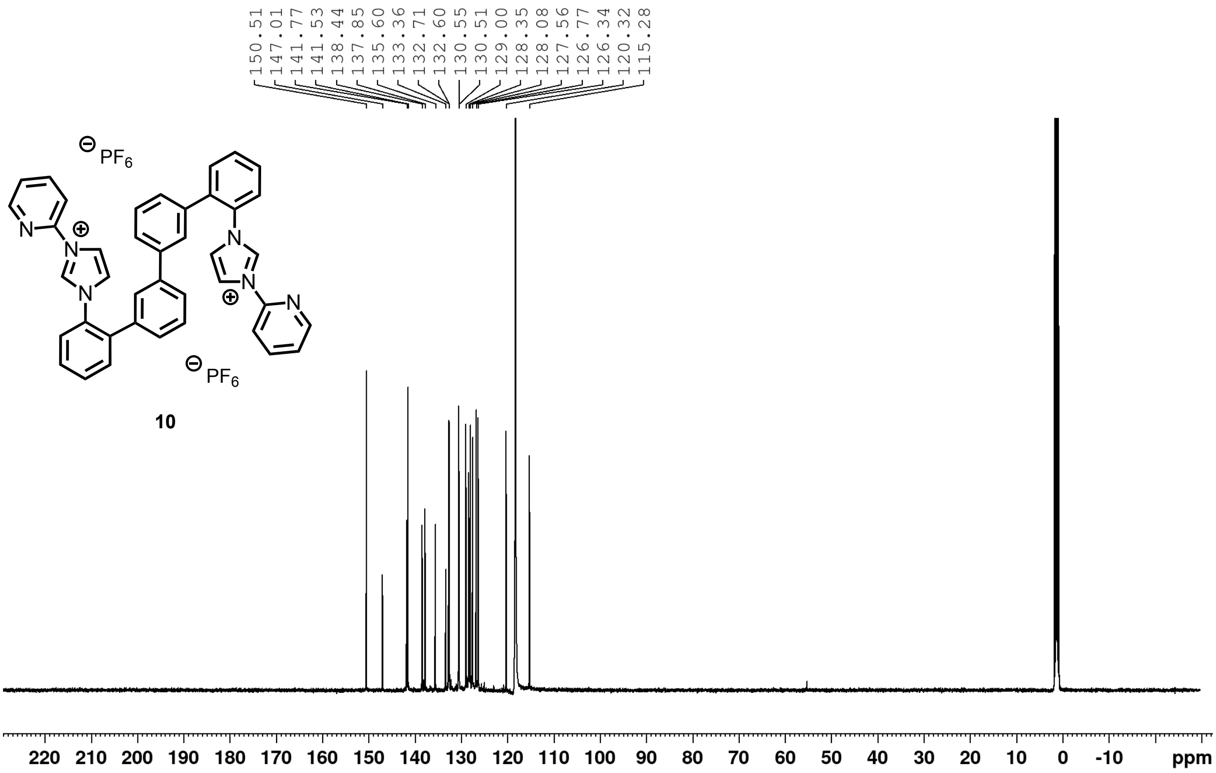

Figure S24: ^{13}C-NMR spectrum of **10** (126 MHz, CD_3CN, 25 °C).

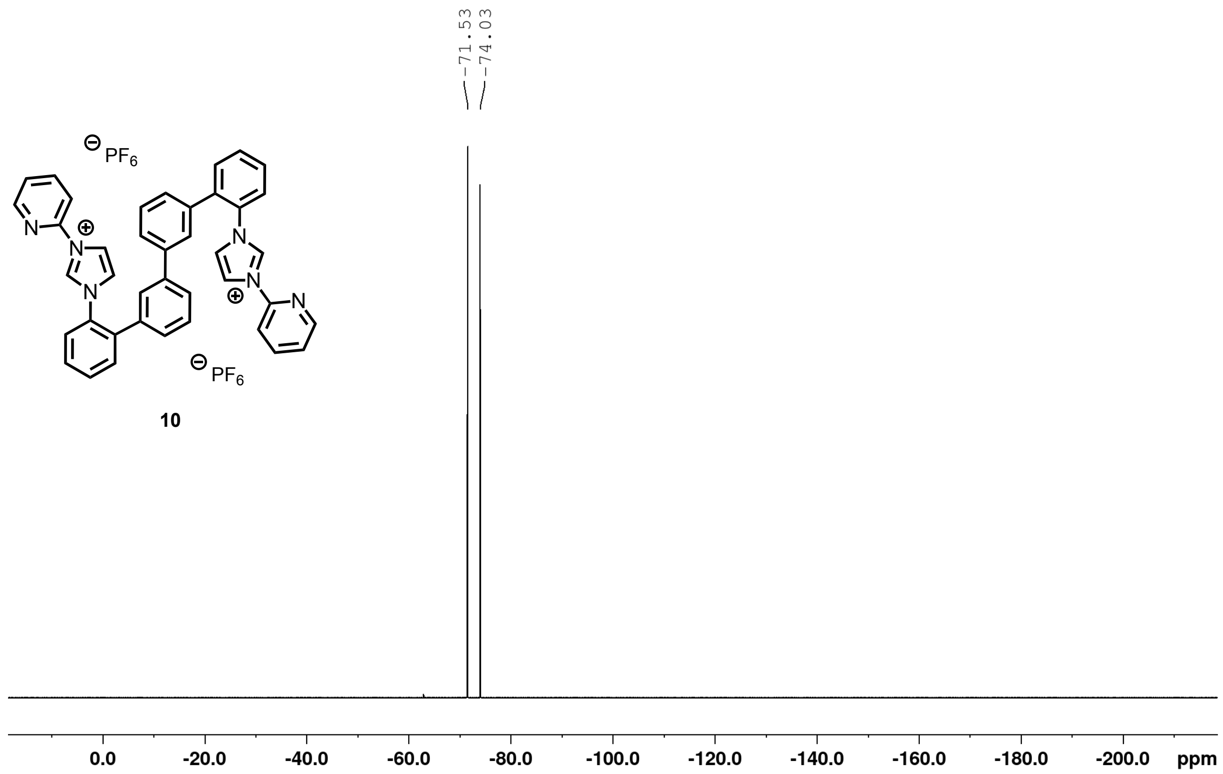

Figure S25: ^{19}F-NMR spectrum of **10** (282 MHz, $CDCl_3$, 25 °C).

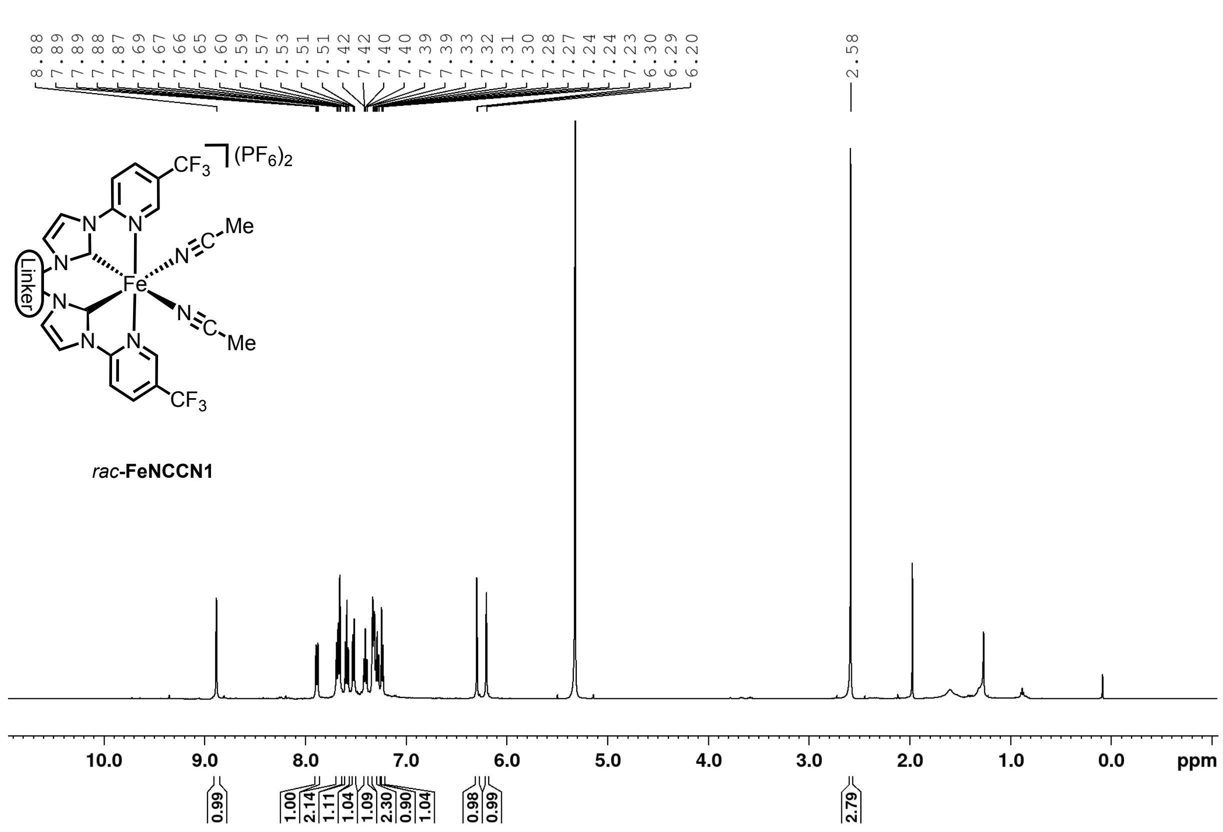

Figure S26: ^{1}H-NMR spectrum of *rac*-**FeNCCN1** (500 MHz, CD_2Cl_2, 25 °C).

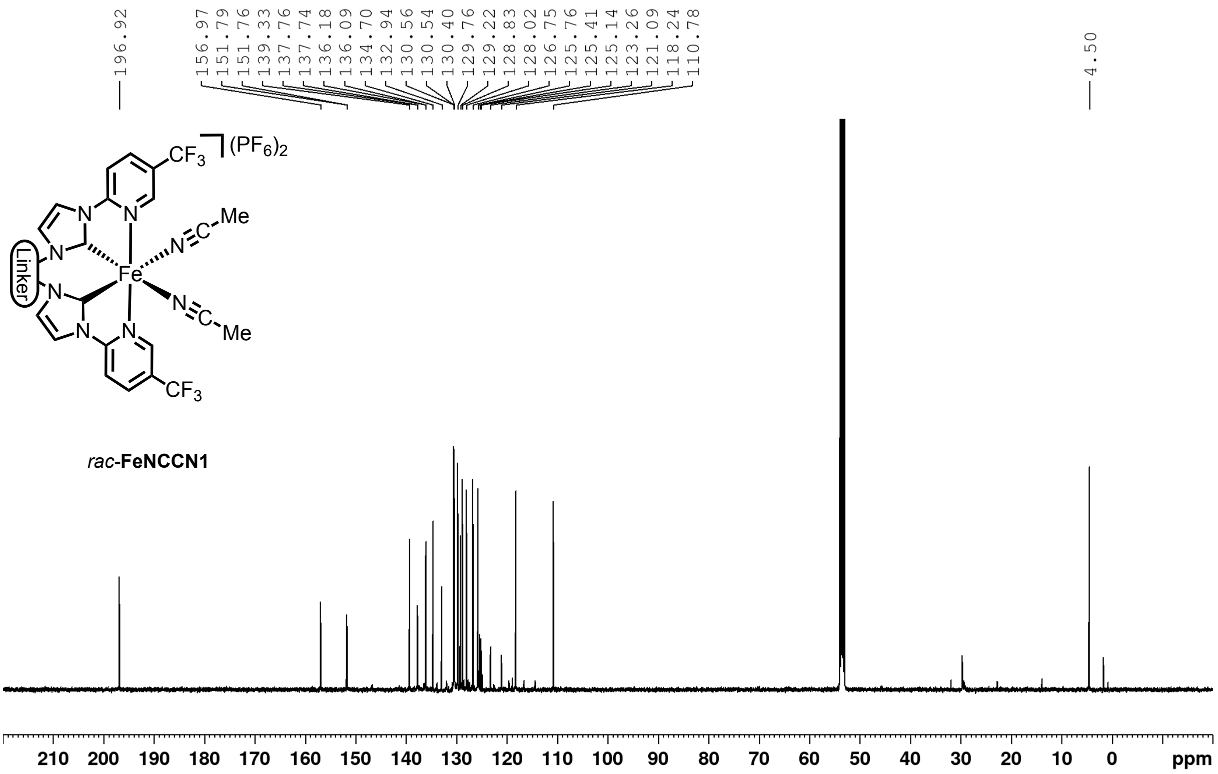

Figure S27: ^{13}C-NMR spectrum of *rac*-**FeNCCN1** (126 MHz, CD_2Cl_2, 25 °C).

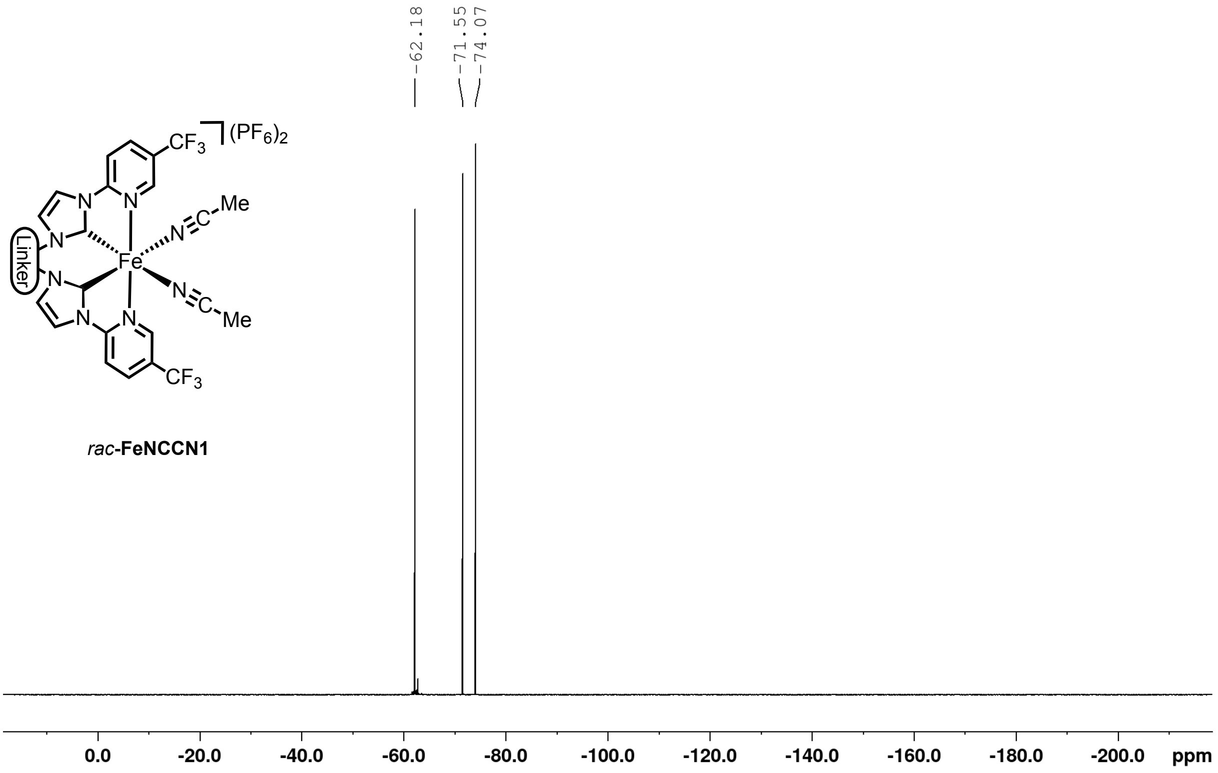

Figure S28: ^{19}F-NMR spectrum of *rac*-**FeNCCN1** (282 MHz, CD_2Cl_2, 25 °C).

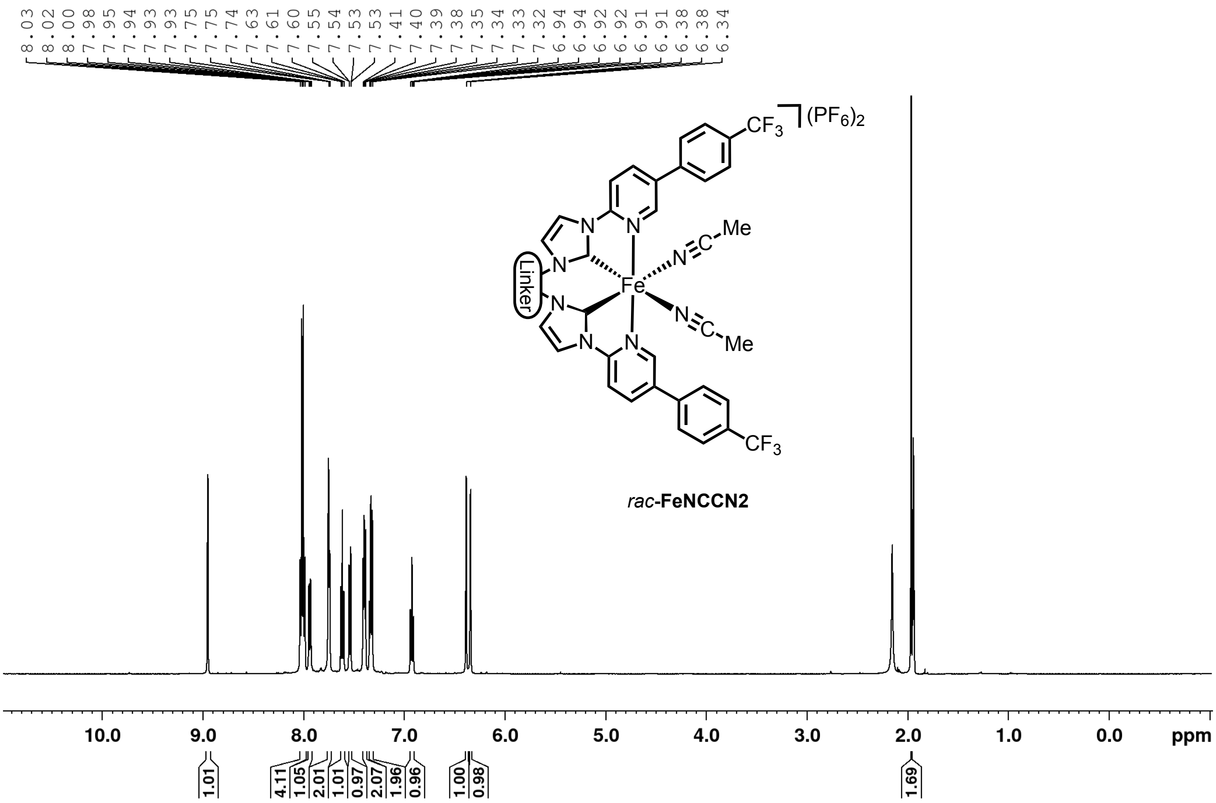

Figure S29: ^{1}H-NMR spectrum of *rac*-**FeNCCN2** (500 MHz, CD_3CN, 25 °C).

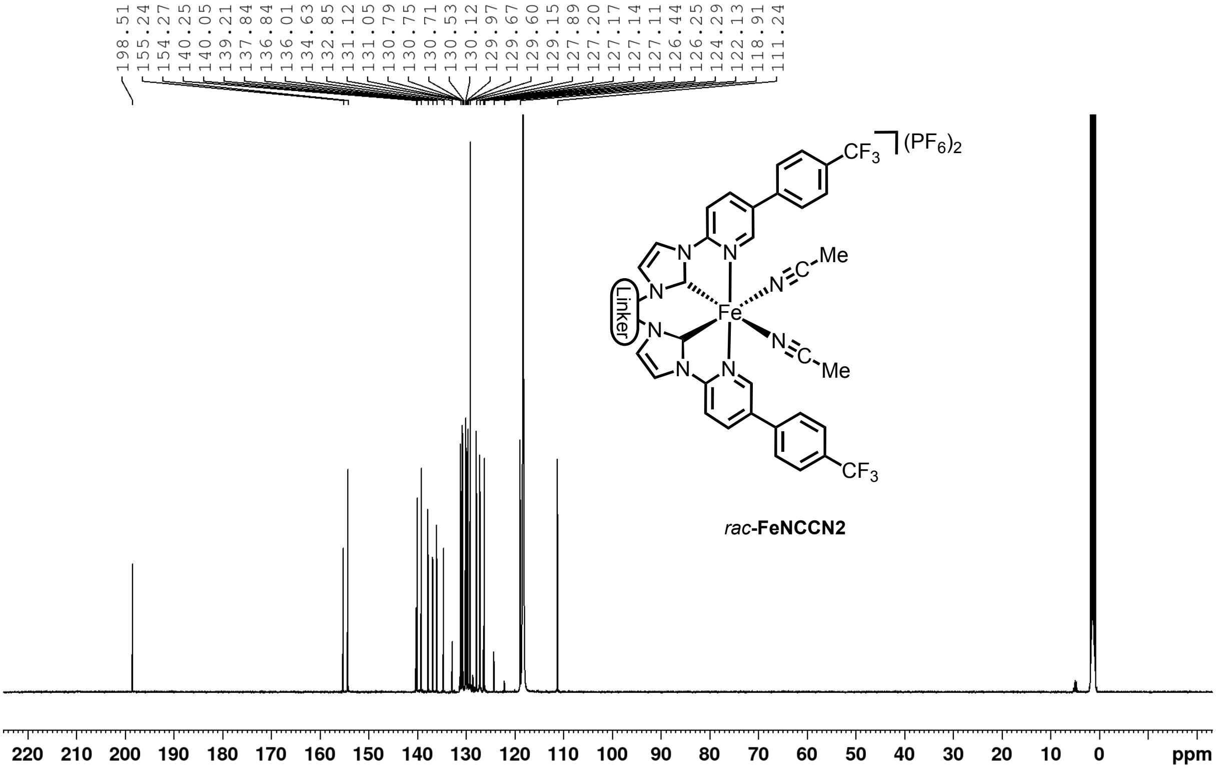

Figure S30: ^{13}C-NMR spectrum of *rac*-**FeNCCN2** (126 MHz, CD_3CN, 25 °C).

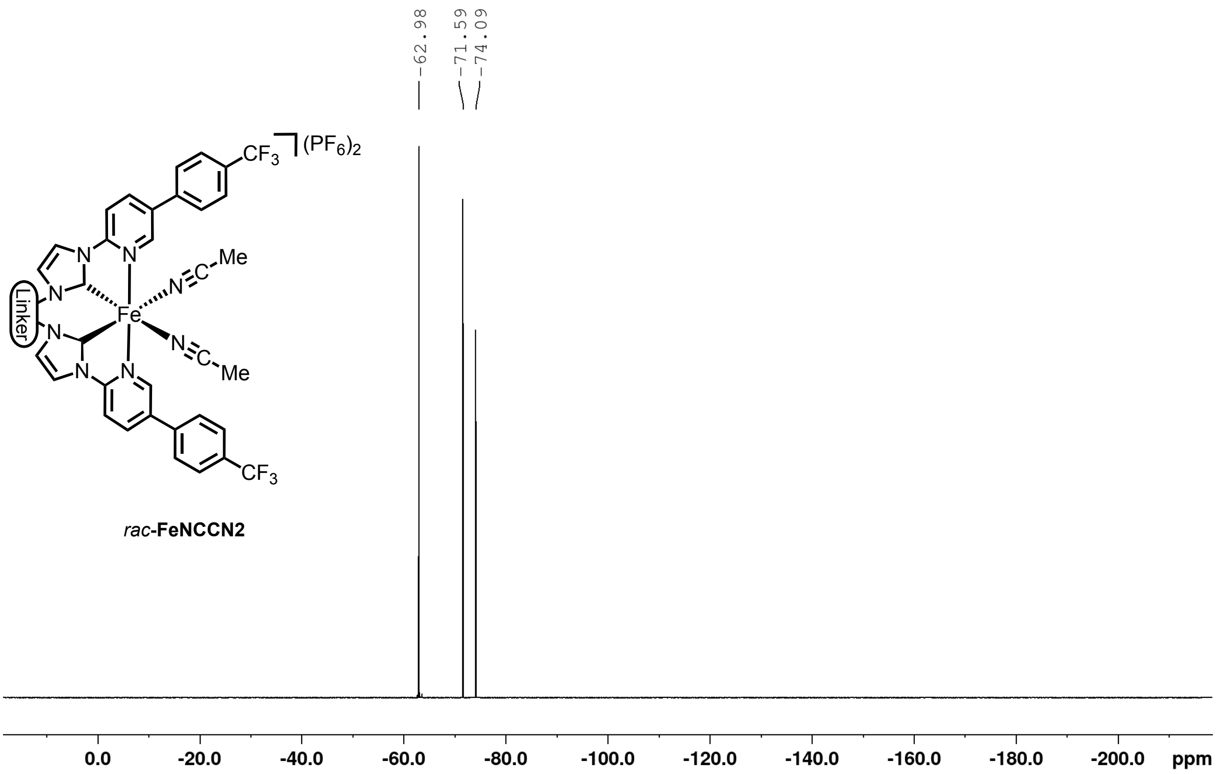

Figure S31: ^{19}F-NMR spectrum of *rac*-**FeNCCN2** (282 MHz, CD_3CN, 25 °C).

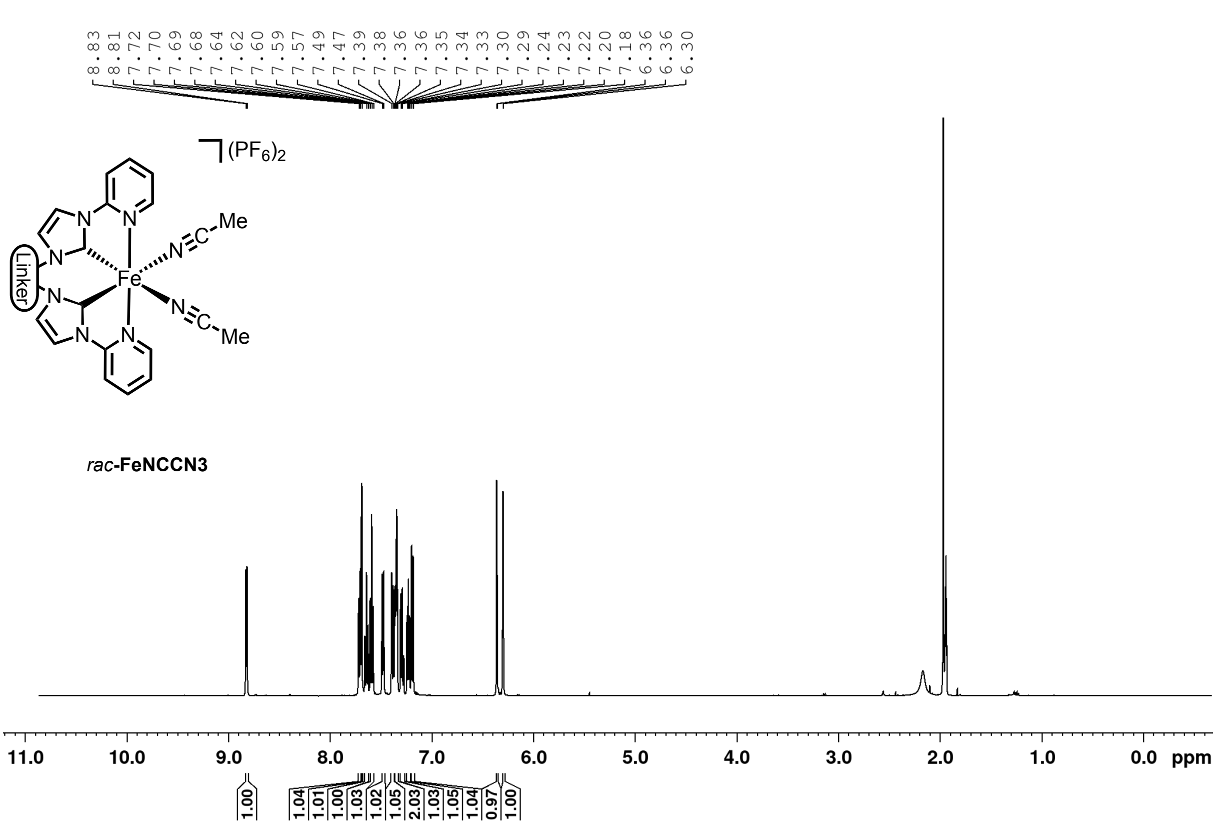

Figure S32: ^{1}H-NMR spectrum of *rac*-**FeNCCN3** (500 MHz, CD_3CN, 25 °C).

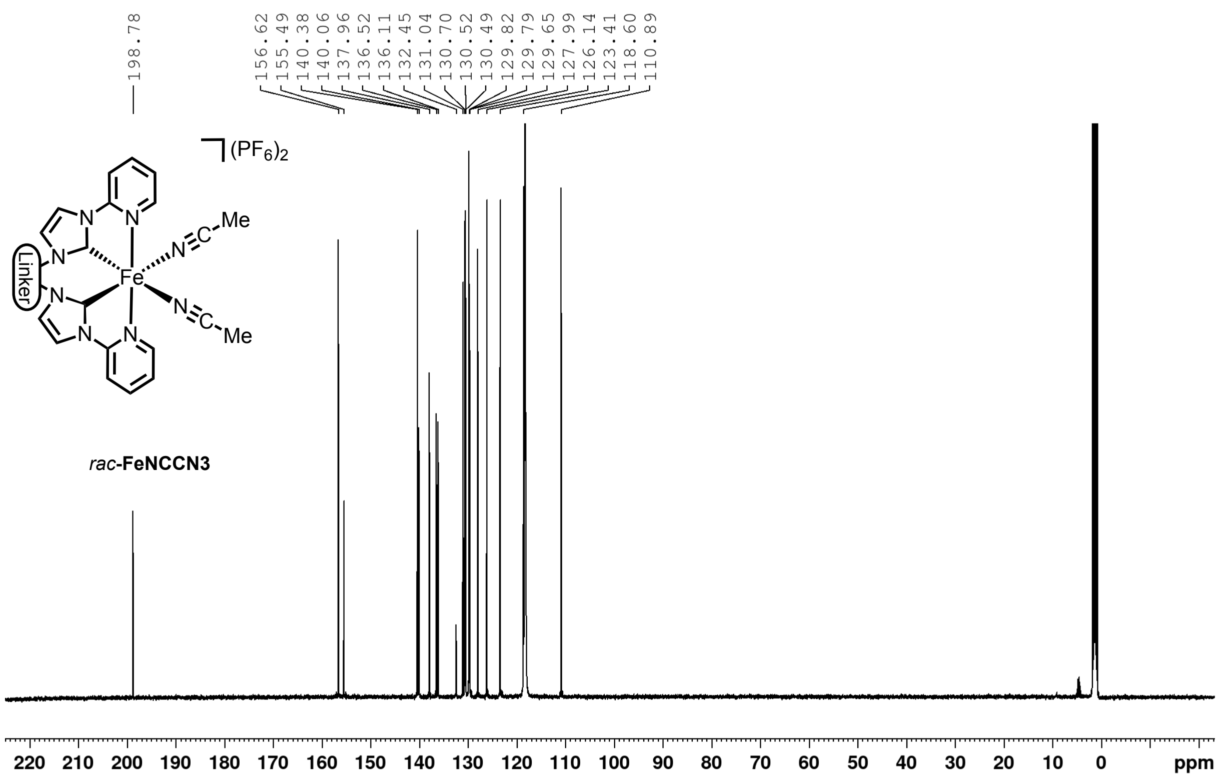

Figure S33: ^{13}C-NMR spectrum of *rac*-**FeNCCN3** (126 MHz, CD_3CN, 25 °C).

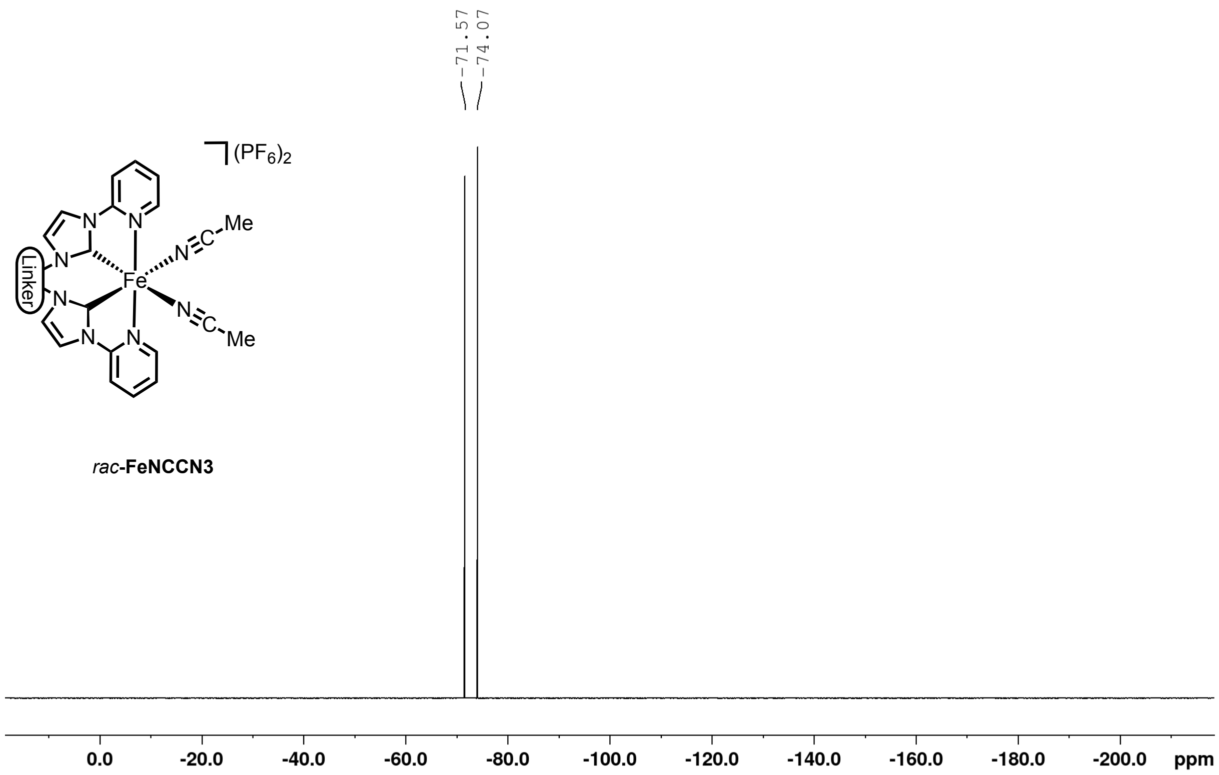

Figure S34: ^{19}F-NMR spectrum of *rac*-**FeNCCN3** (282 MHz, CD_3CN, 25 °C).

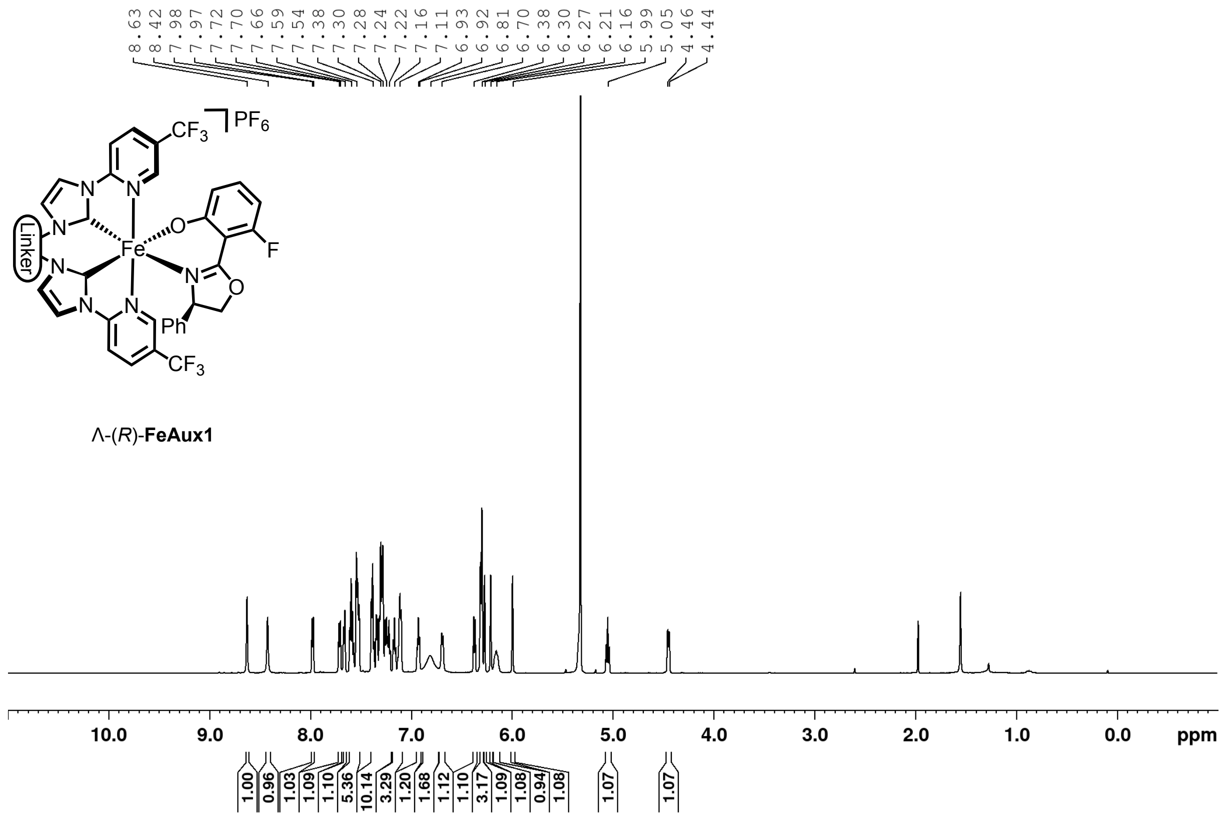

Figure S35: ^{1}H-NMR spectrum of Λ-(*R*)-**FeAux1** (600 MHz, CD_2Cl_2, 25 °C).

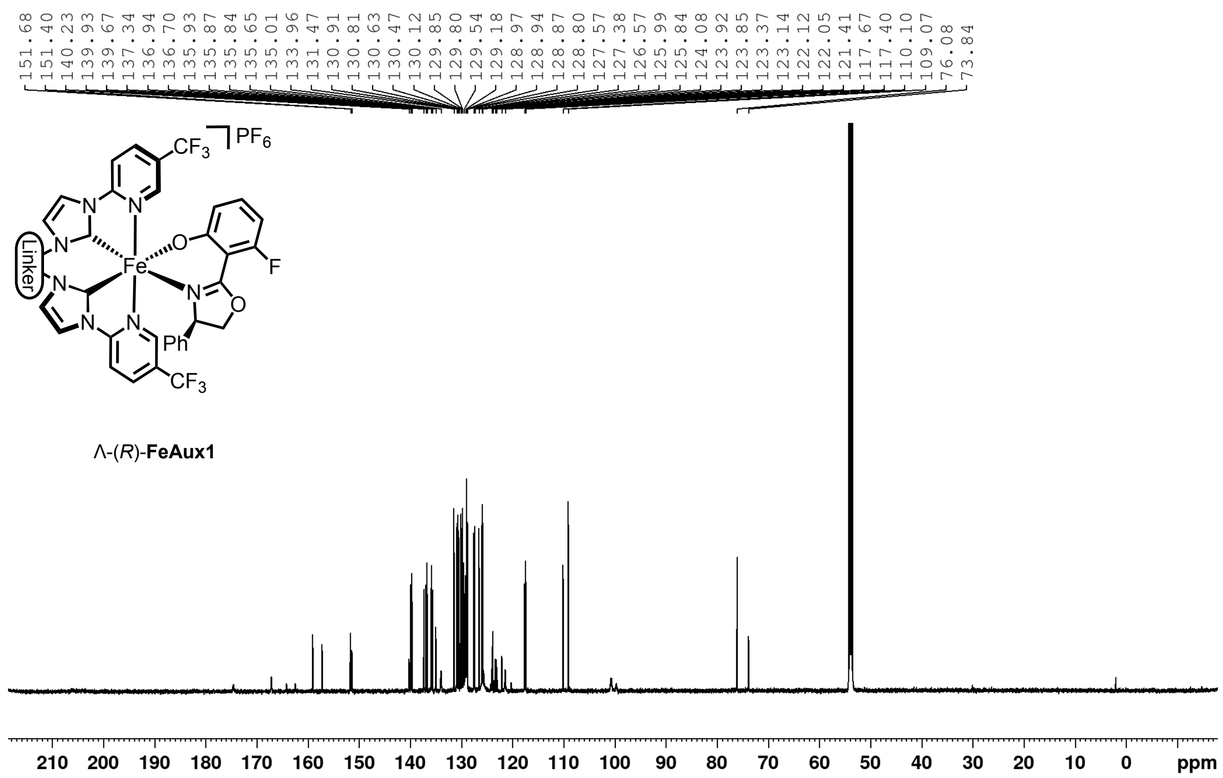

Figure S36: ^{13}C-NMR spectrum of Λ-(*R*)-**FeAux1** (151 MHz, CD_2Cl_2, 25 °C).

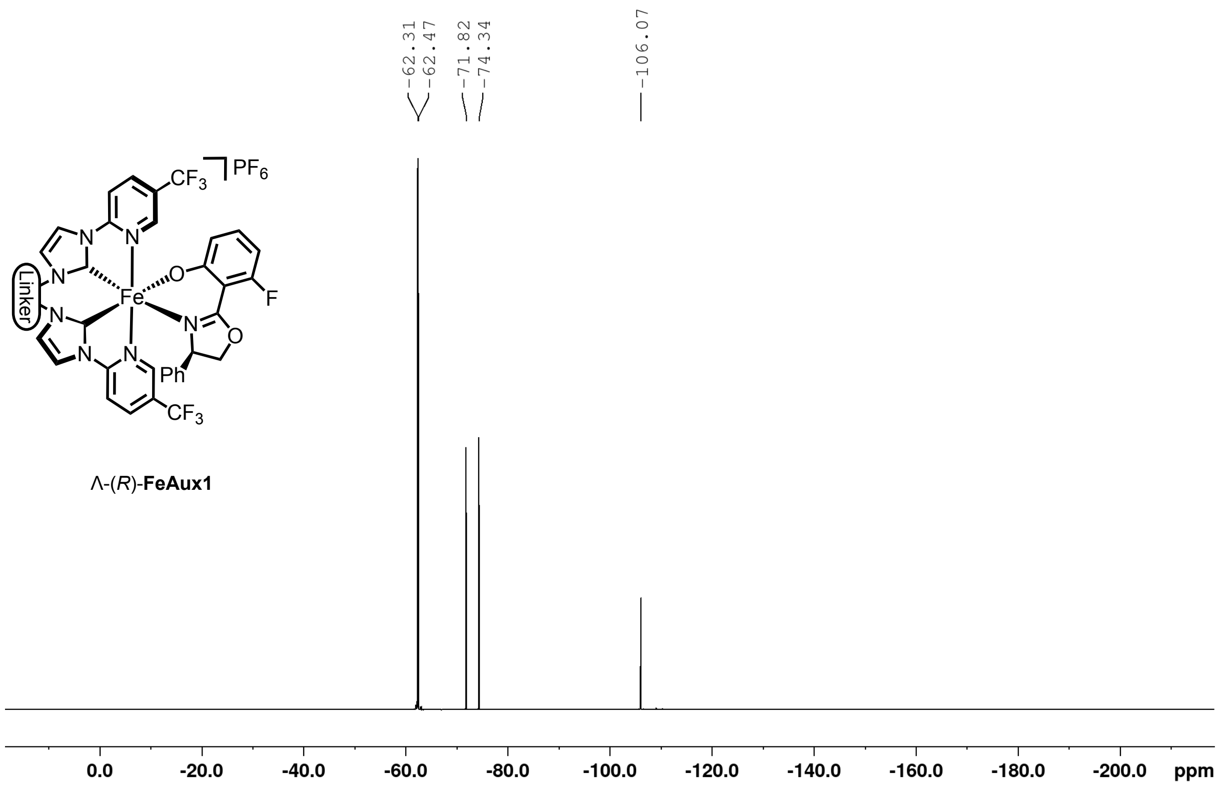

Figure S37: ^{19}F-NMR spectrum of Λ-(*R*)-**FeAux1** (282 MHz, CD_2Cl_2, 25 °C).

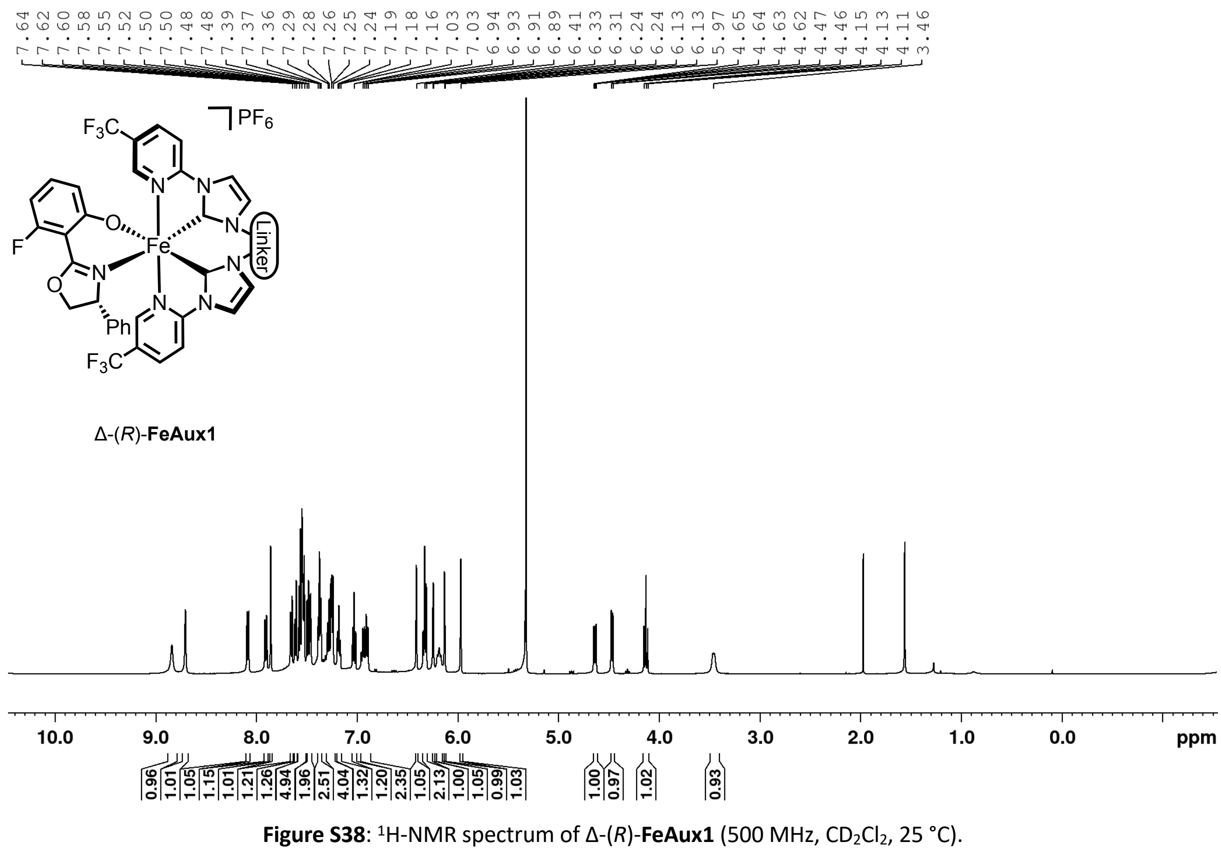

Figure S38: ^{1}H-NMR spectrum of Δ-(*R*)-**FeAux1** (500 MHz, CD_2Cl_2, 25 °C).

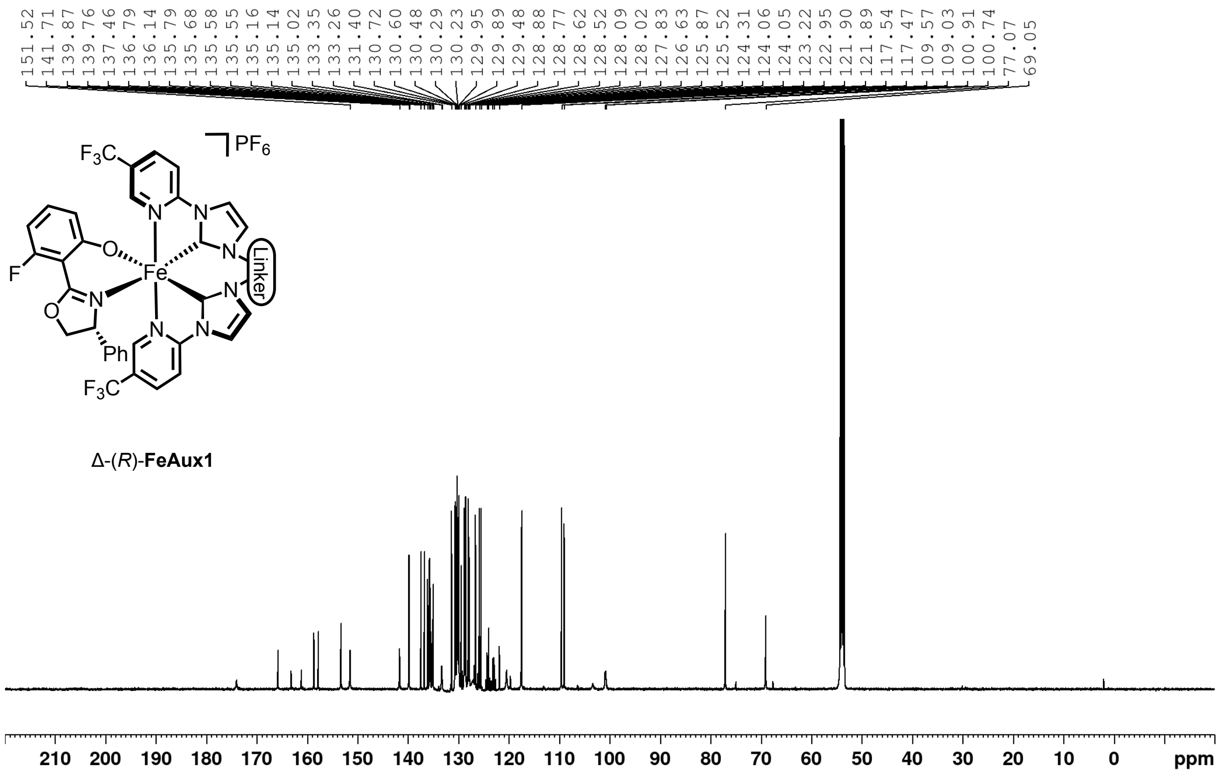

Figure S39: ^{13}C-NMR spectrum of Δ-(*R*)-**FeAux1** (126 MHz, CD_2Cl_2, 25 °C).

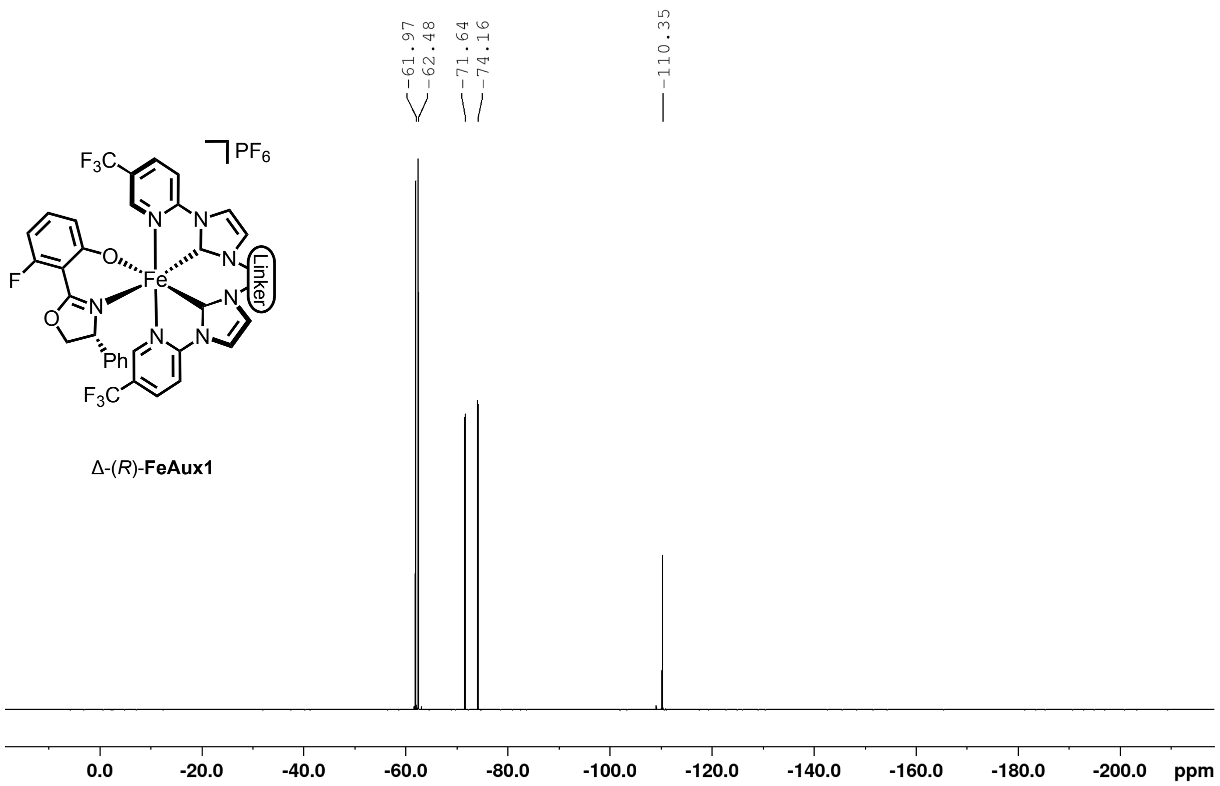

Figure S40: ^{19}F-NMR spectrum of Δ-(*R*)-**FeAux1** (282 MHz, CD_2Cl_2, 25 °C).

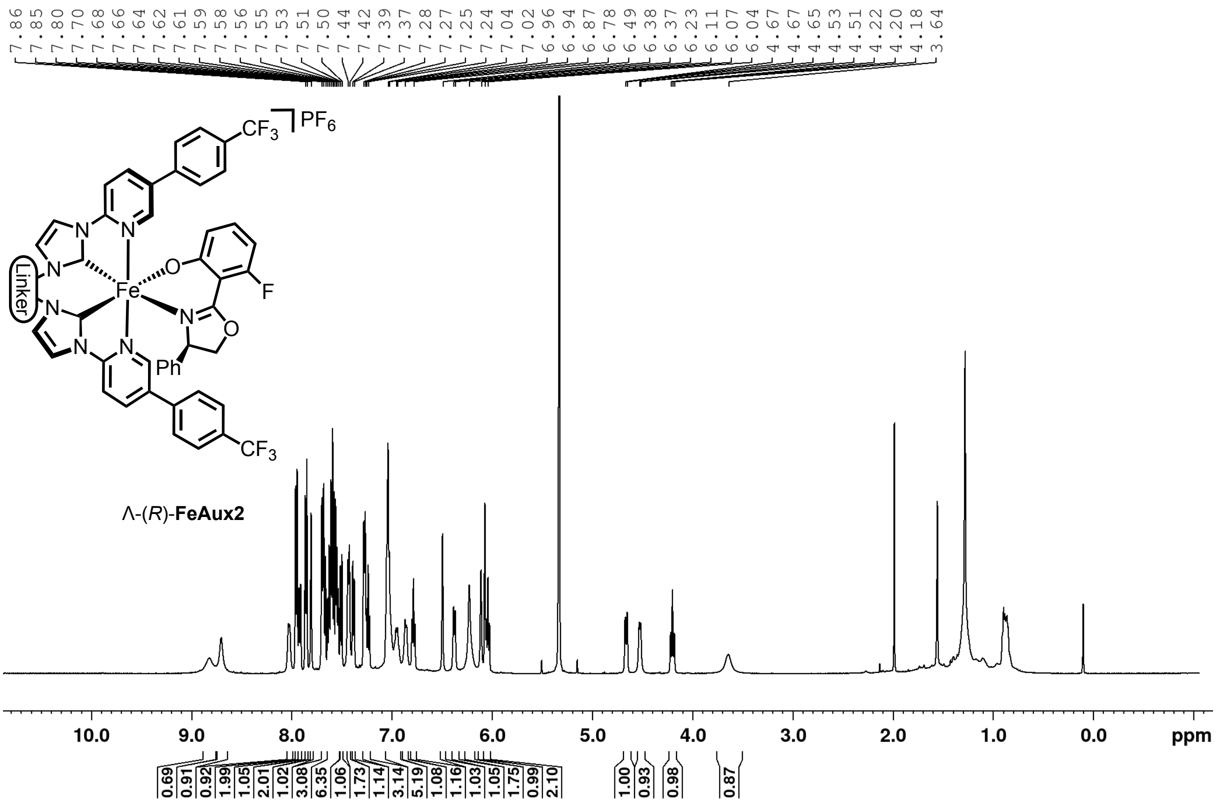

Figure S41: ^{1}H-NMR spectrum of Λ-(*R*)-**FeAux2** (500 MHz, CD_2Cl_2, 25 °C).

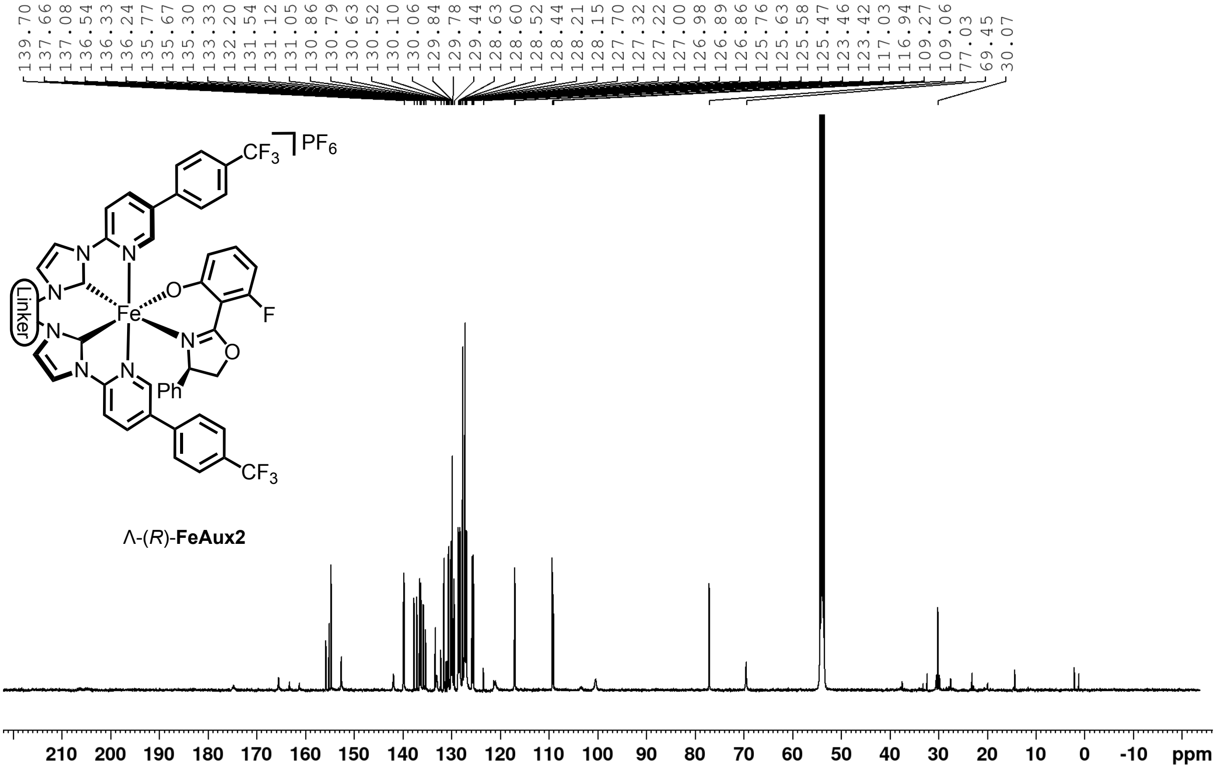

Figure S42: ^{13}C-NMR spectrum of Λ-(*R*)-**FeAux2** (126 MHz, CD_2Cl_2, 25 °C).

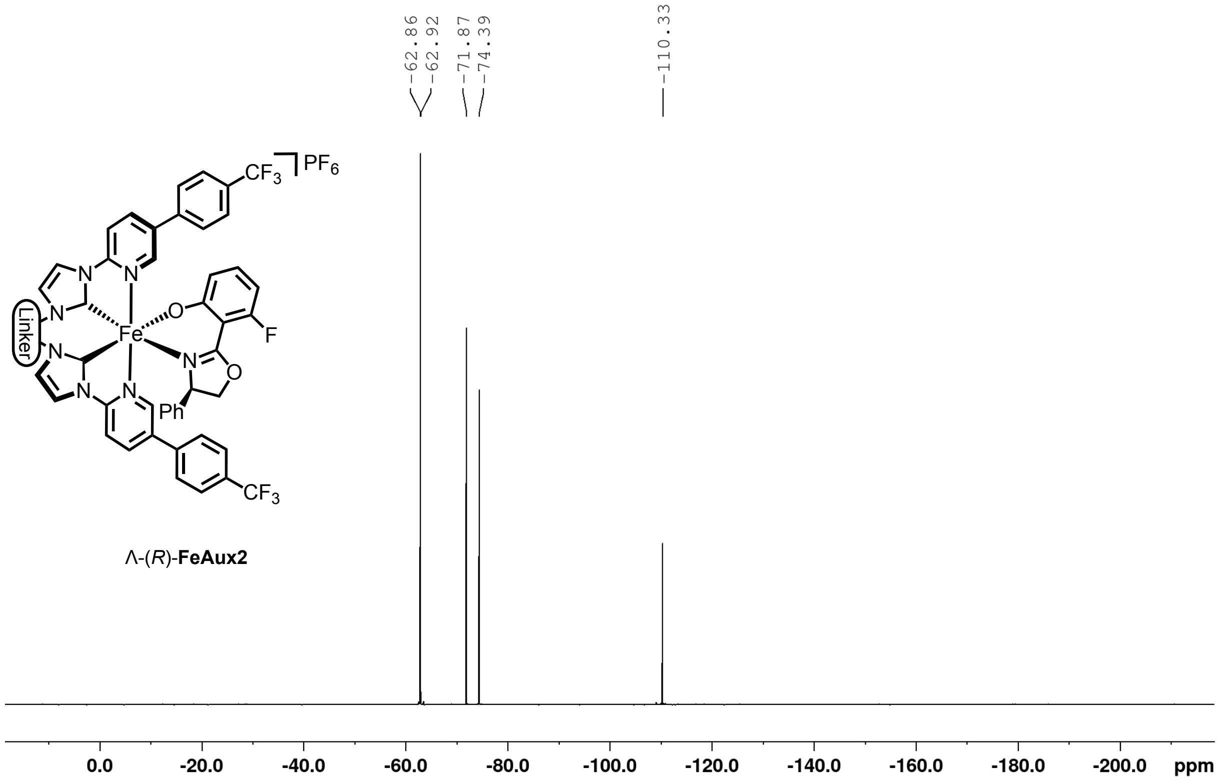

Figure S43: ^{19}F-NMR spectrum of Λ-(*R*)-**FeAux2** (282 MHz, CD_2Cl_2, 25 °C).

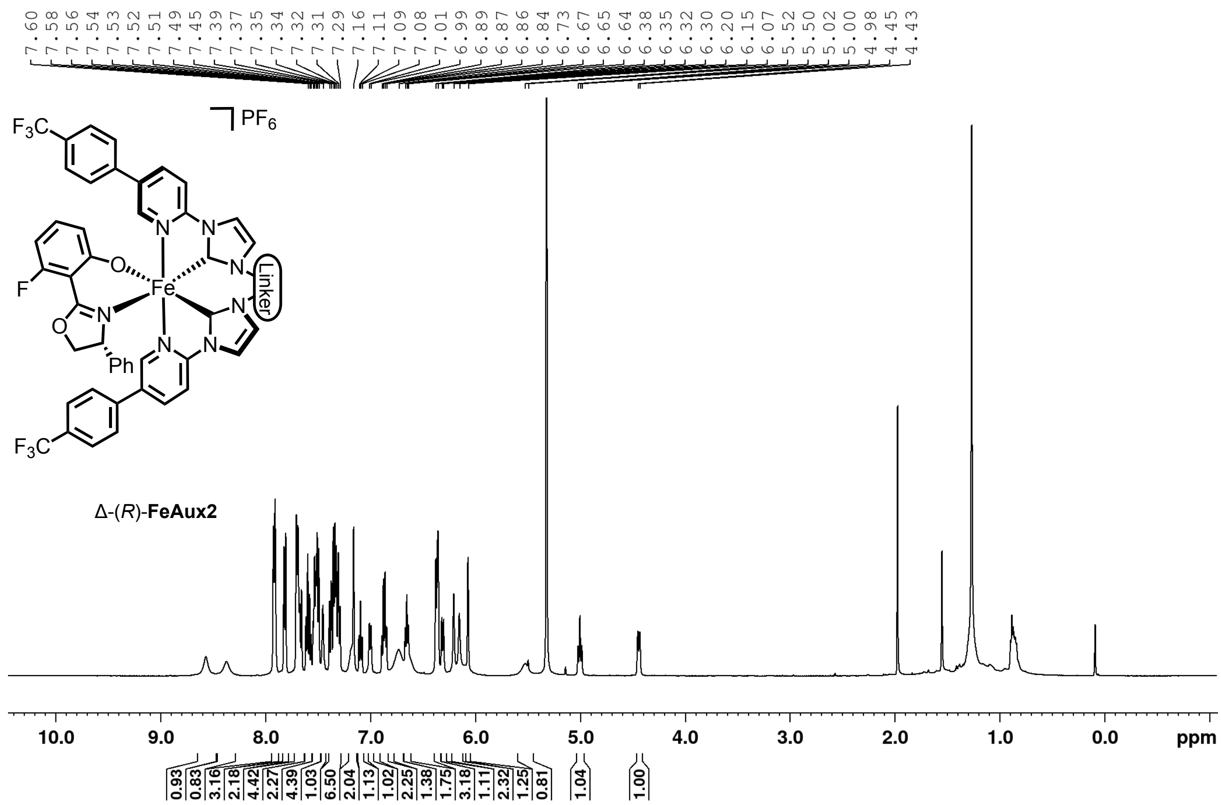

Figure S 44: ^{1}H-NMR spectrum of Δ-(*R*)-**FeAux2** (500 MHz, CD_2Cl_2, 25 °C).

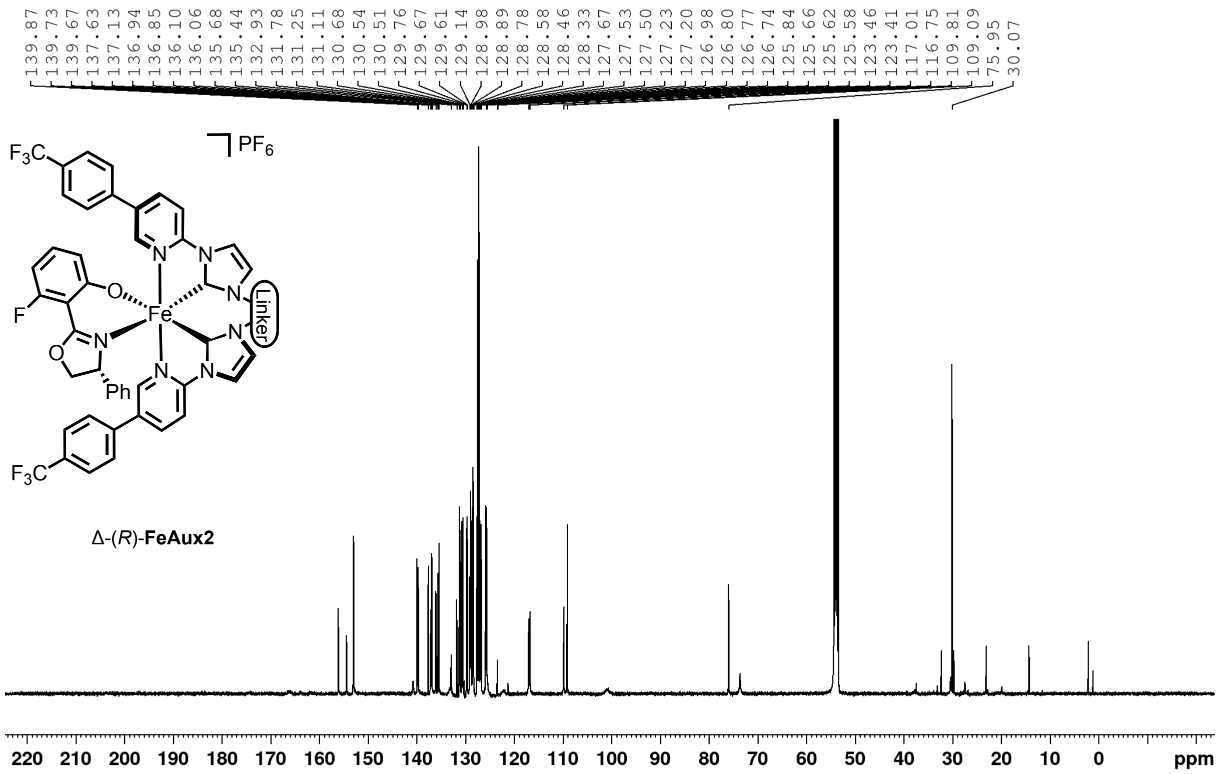

Figure S45: ^{13}C-NMR spectrum of Δ-(*R*)-**FeAux2** (126 MHz, CD_2Cl_2, 25 °C).

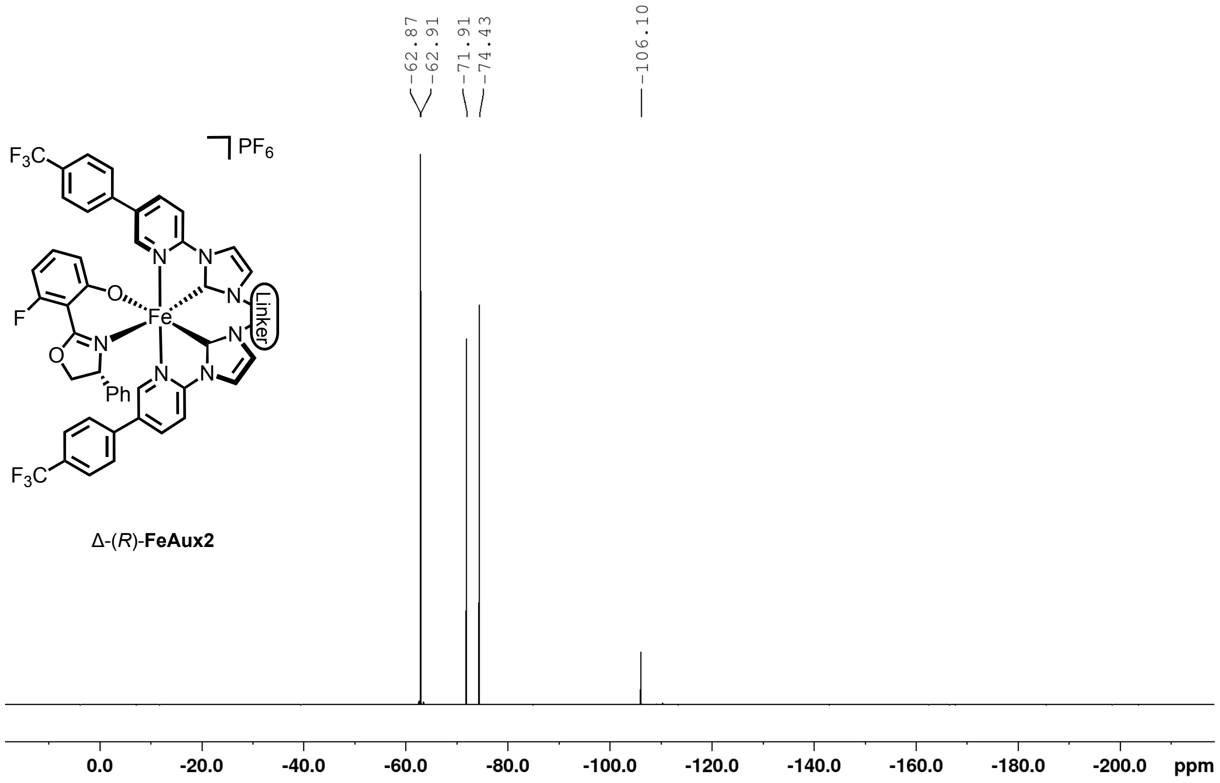

Figure S46: ^{19}F-NMR spectrum of Δ-(*R*)-**FeAux2** (282 MHz, CD_2Cl_2, 25 °C).

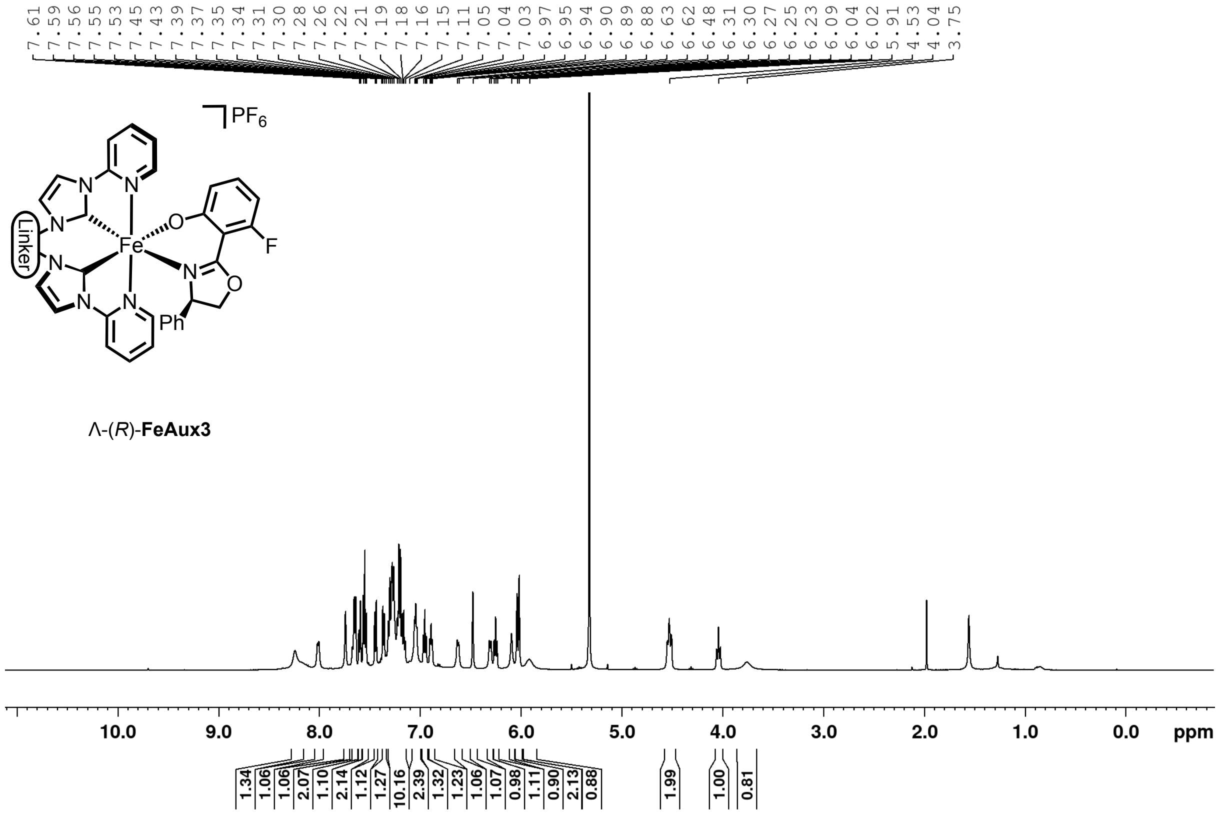

Figure S47: ^{1}H-NMR spectrum of Λ-(*R*)-**FeAux3** (500 MHz, CD_2Cl_2, 25 °C).

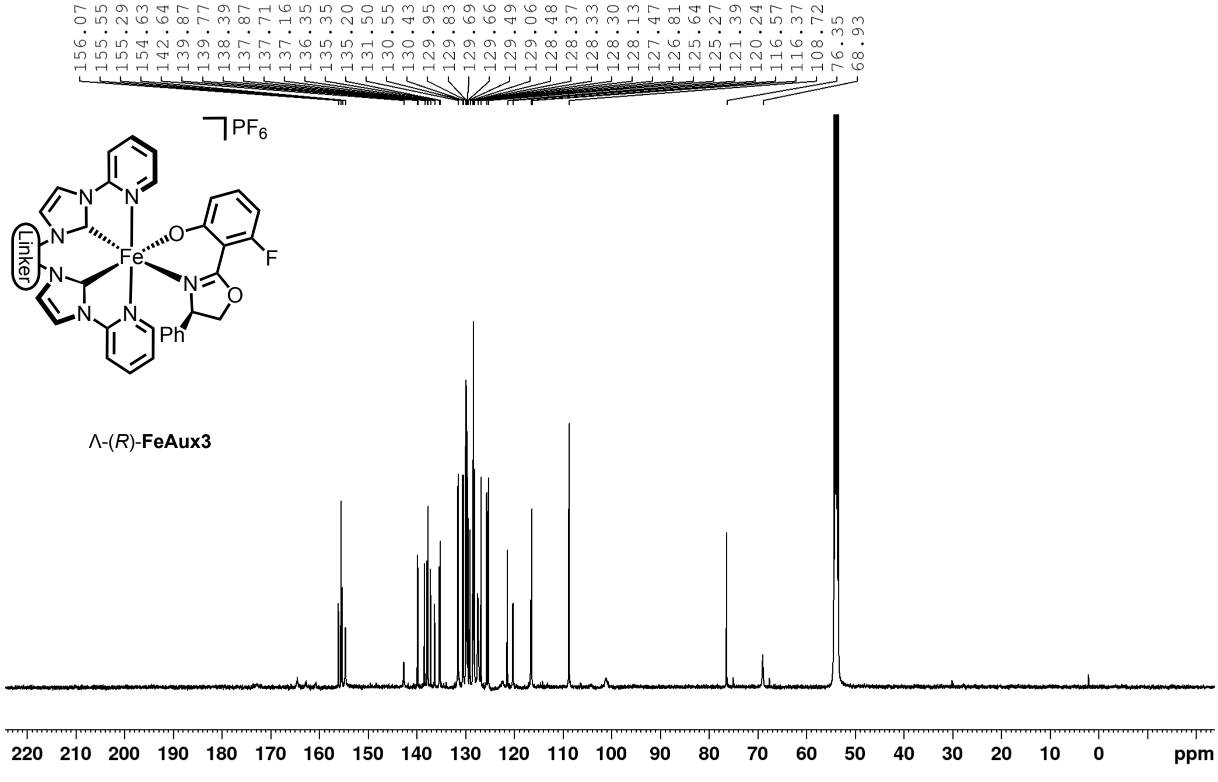

Figure S48: ^{13}C-NMR spectrum of Λ-(*R*)-**FeAux3** (126 MHz, CD_2Cl_2, 25 °C).

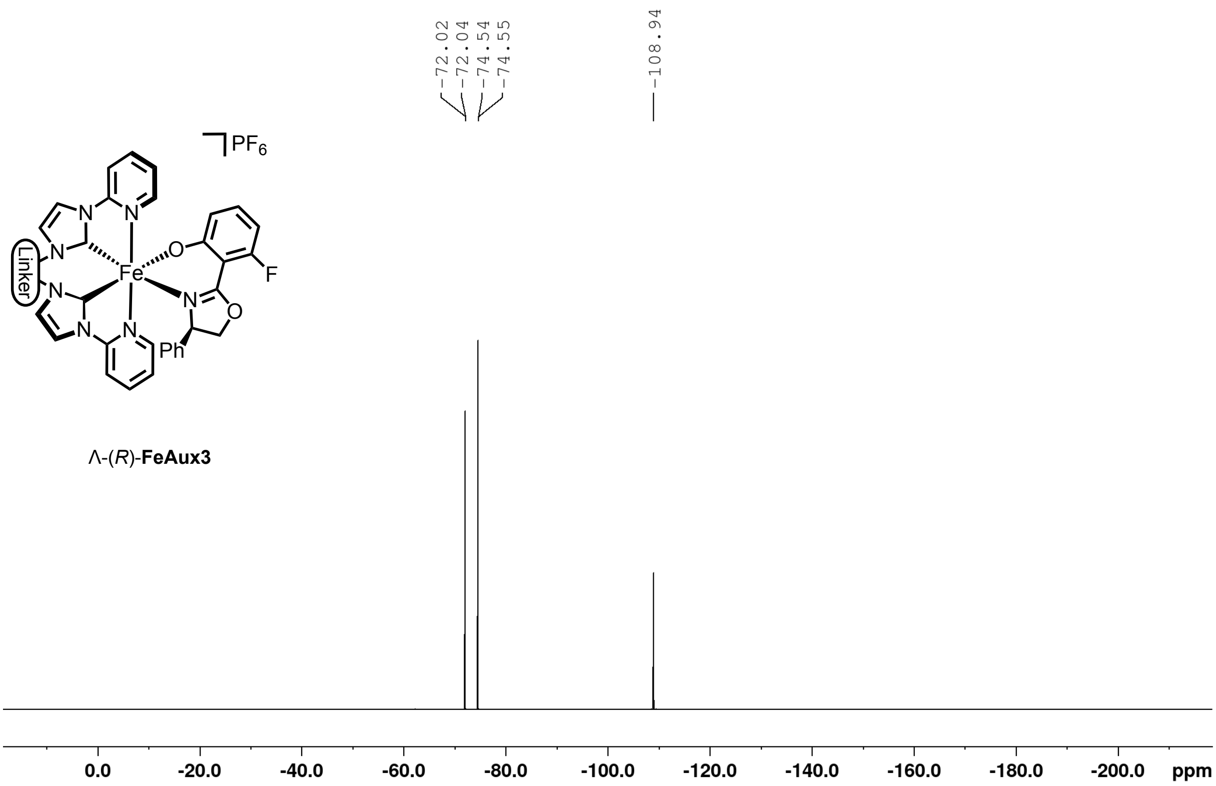

Figure S49: ^{19}F-NMR spectrum of Λ-(*R*)-**FeAux3** (282 MHz, CD_2Cl_2, 25 °C).

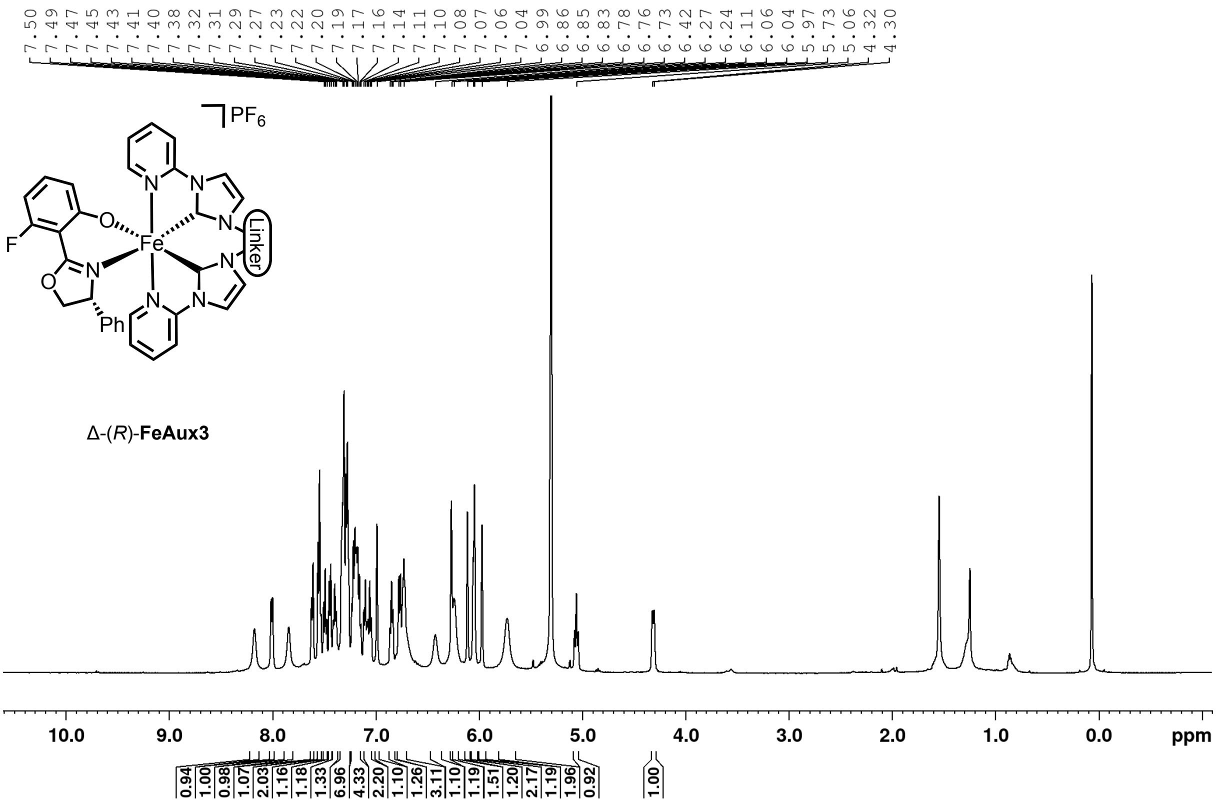

Figure S50: ^{1}H-NMR spectrum of Δ-(*R*)-**FeAux3** (500 MHz, CD_2Cl_2, 25 °C).

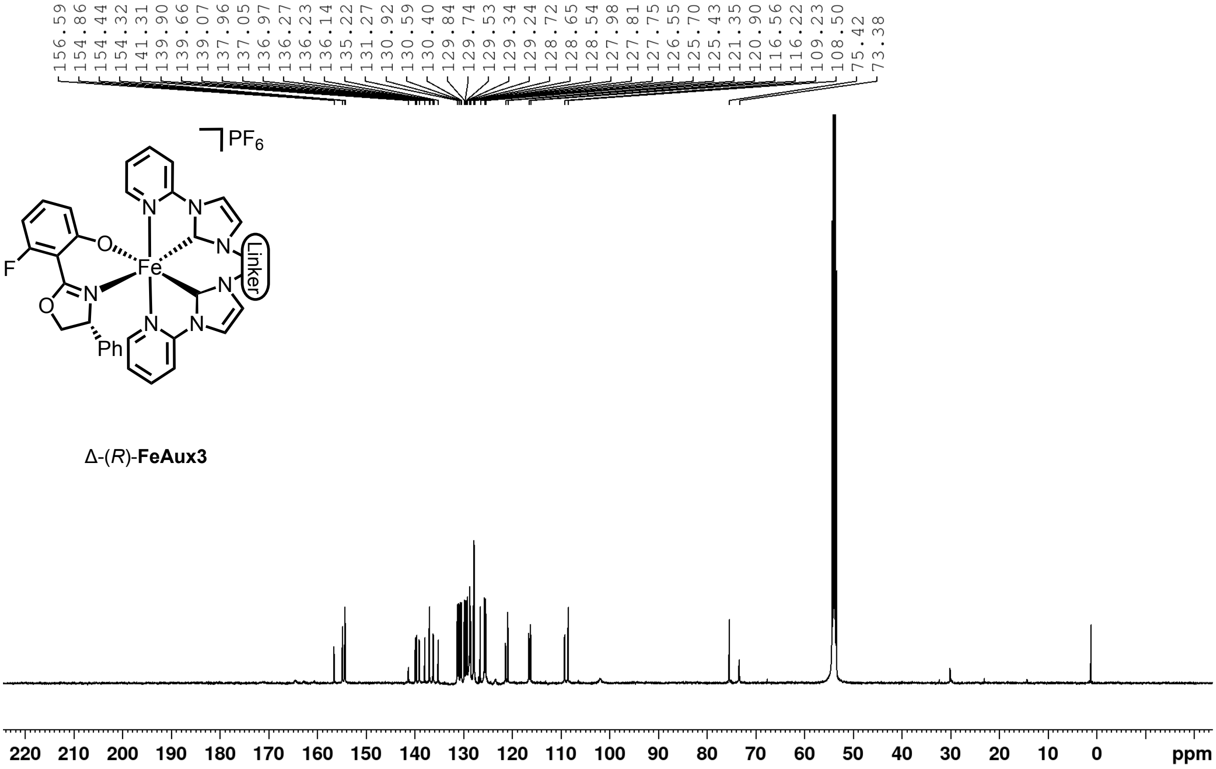

Figure S51: ^{13}C-NMR spectrum of Δ-(*R*)-**FeAux3** (126 MHz, CD_2Cl_2, 25 °C).

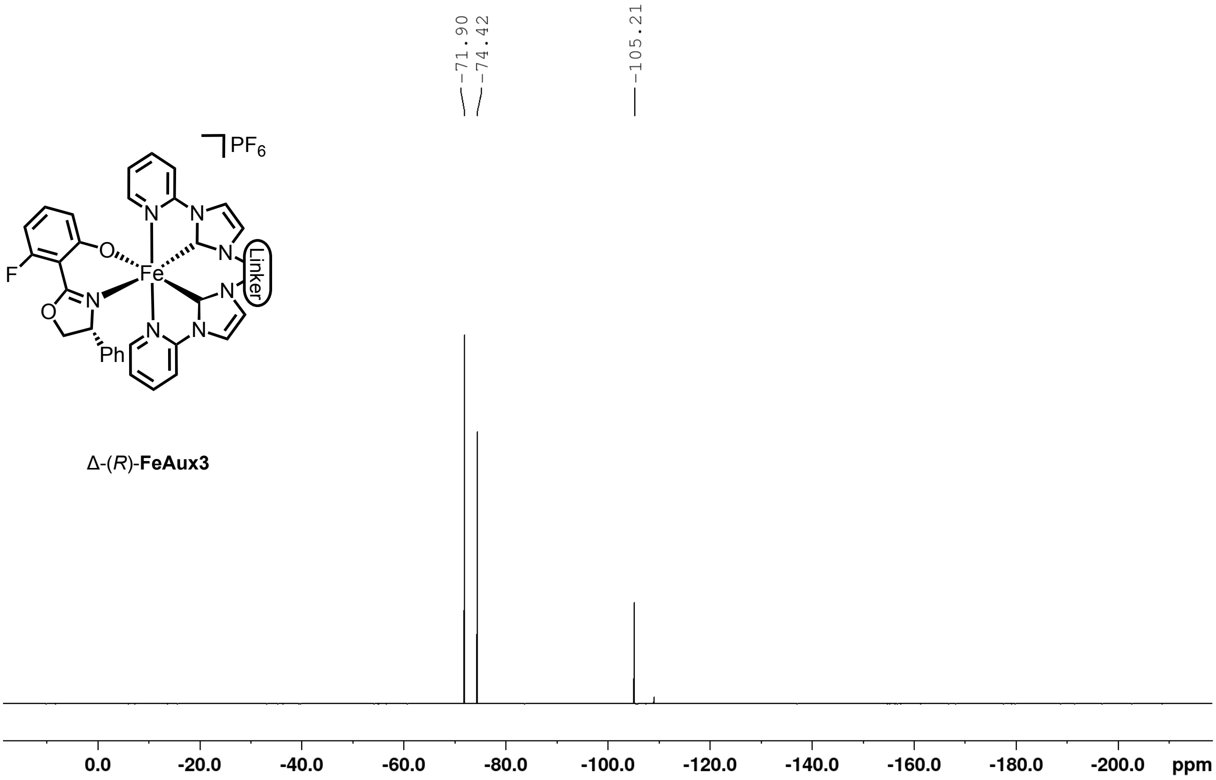

Figure S52: ^{19}F-NMR spectrum of Δ-(*R*)-**FeAux3** (282 MHz, CD_2Cl_2, 25 °C).

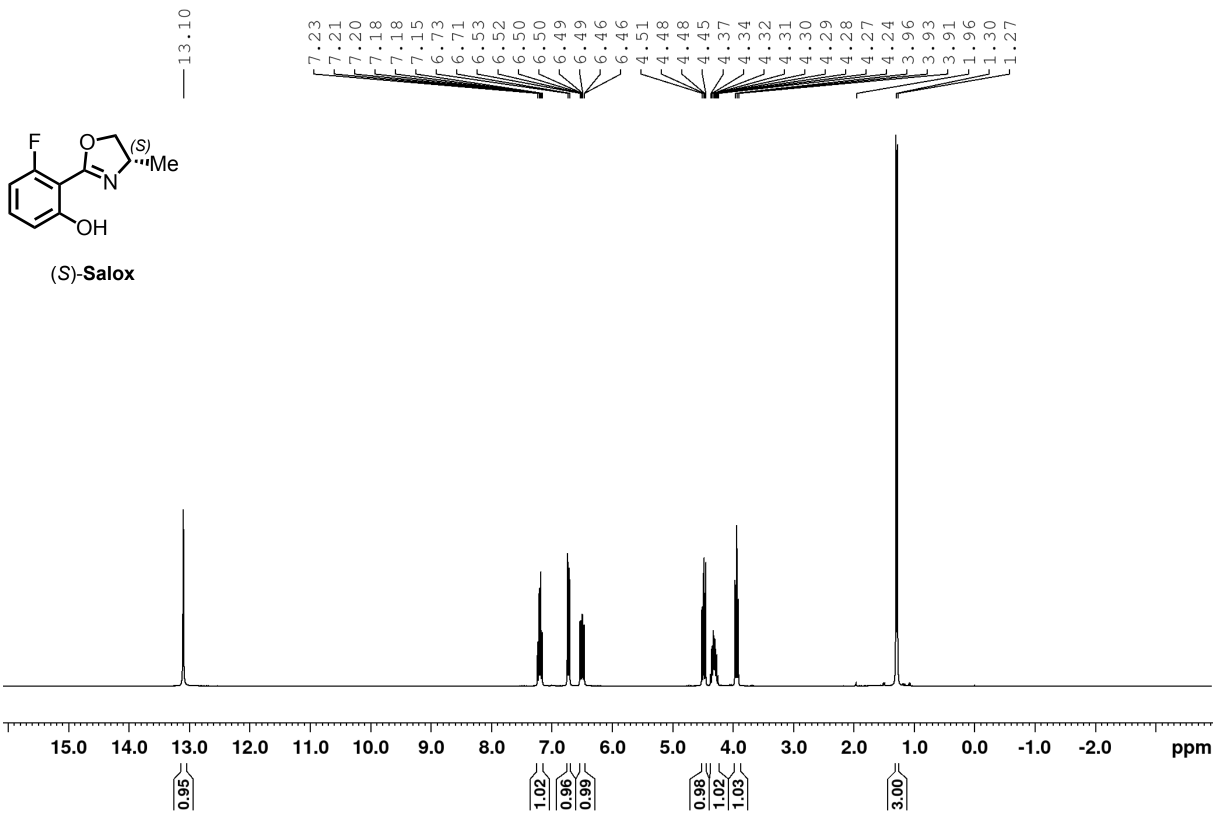

Figure S53: ^{1}H-NMR spectrum of (*S*)-**Salox** (300 MHz, $CDCl_3$, 25 °C).

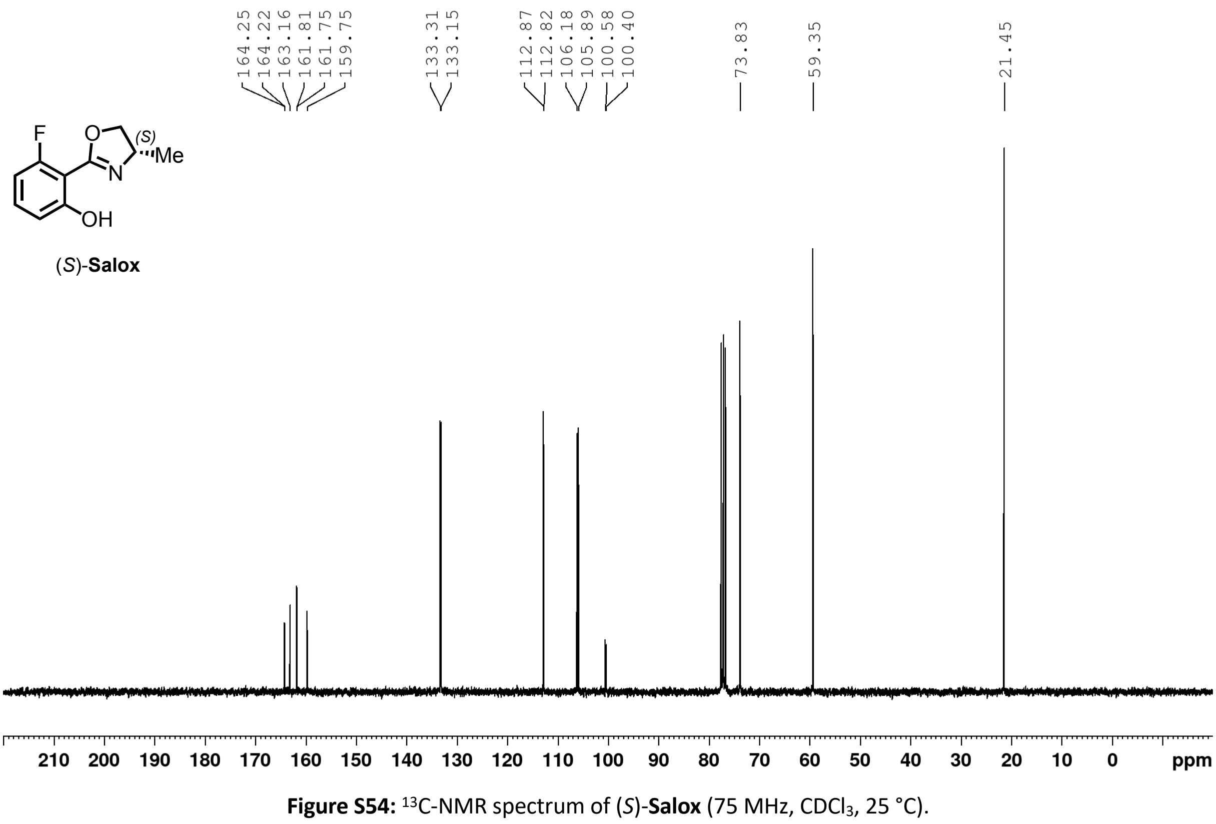

Figure S54: ^{13}C-NMR spectrum of (*S*)-**Salox** (75 MHz, $CDCl_3$, 25 °C).

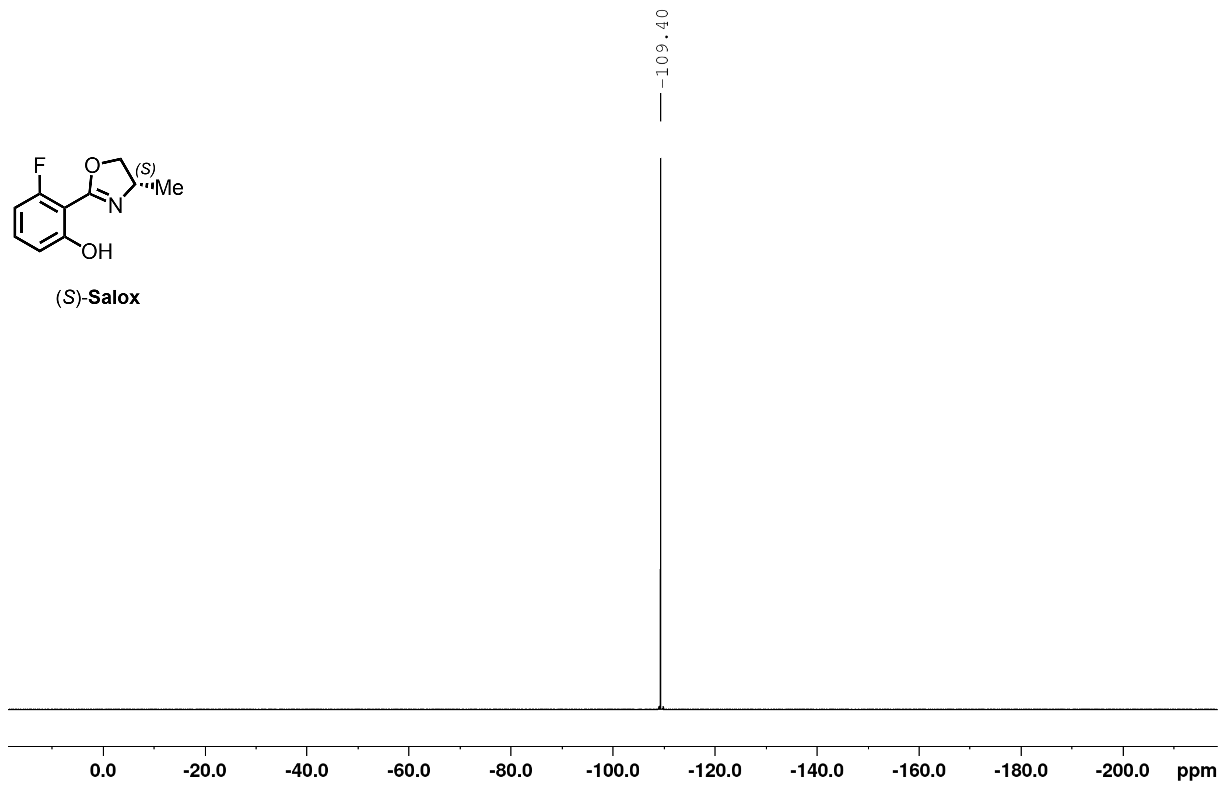

Figure S55: ^{19}F-NMR spectrum of (*S*)-**Salox** (282 MHz, $CDCl_3$, 25 °C).

10. Chiral HPLC Traces

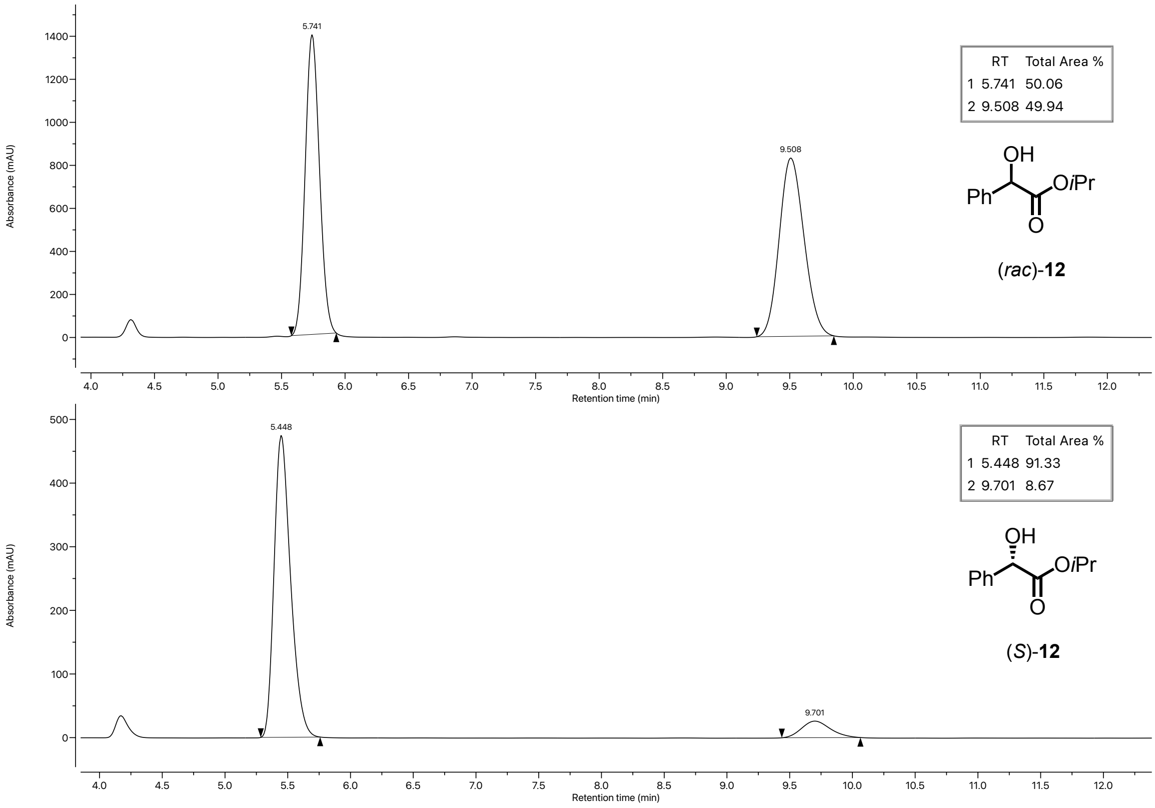

Figure S56: HPLC chromatogram of (*S*)-**12** with 82% ee.

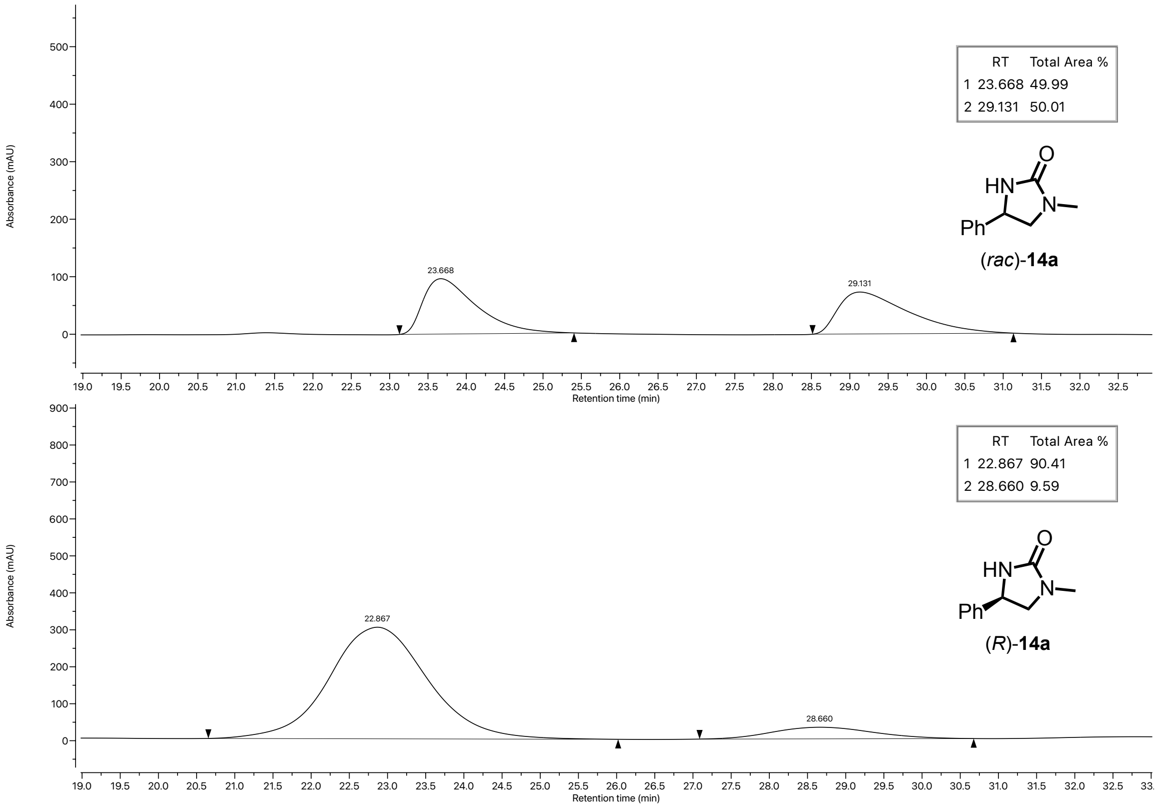

Figure S57: HPLC chromatogram of (*R*)-**14a** with 81% ee.

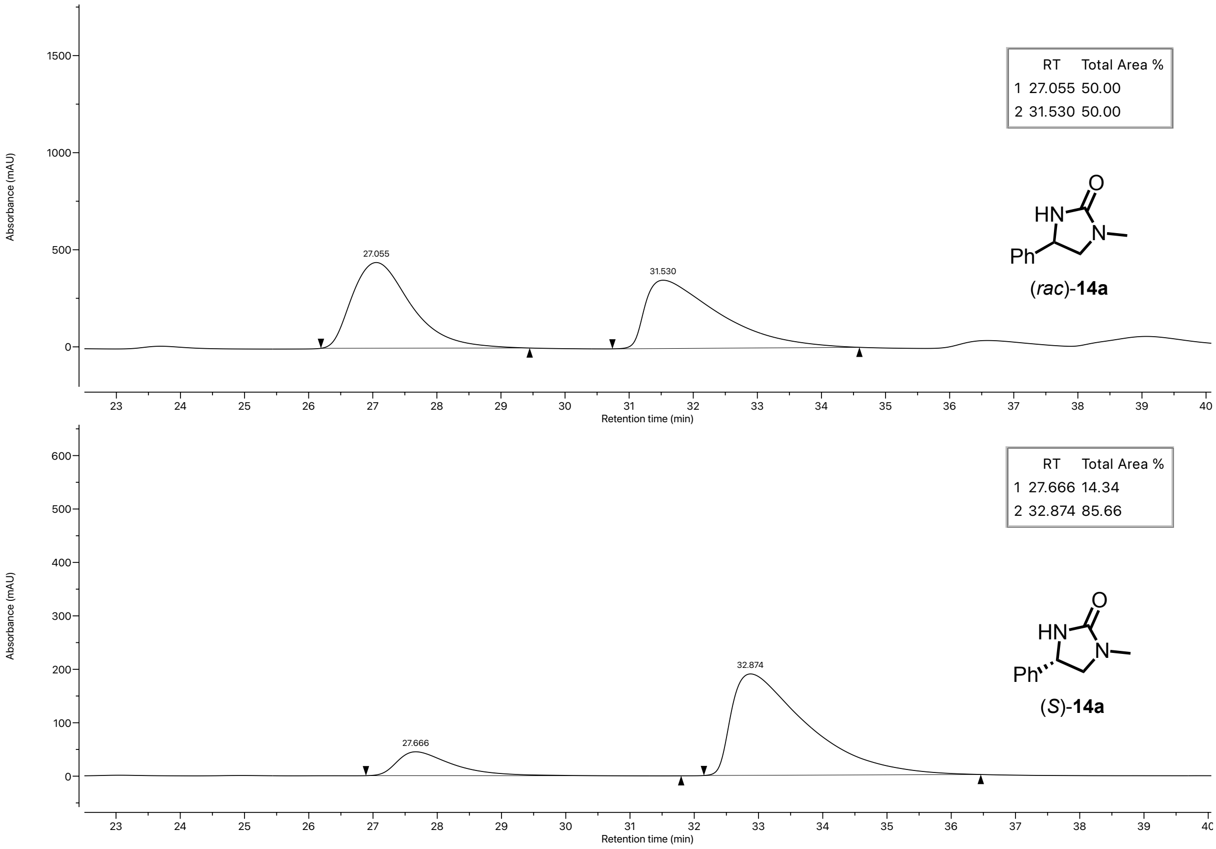

Figure S58: HPLC chromatogram of (*S*)-**14a** with 71% ee.

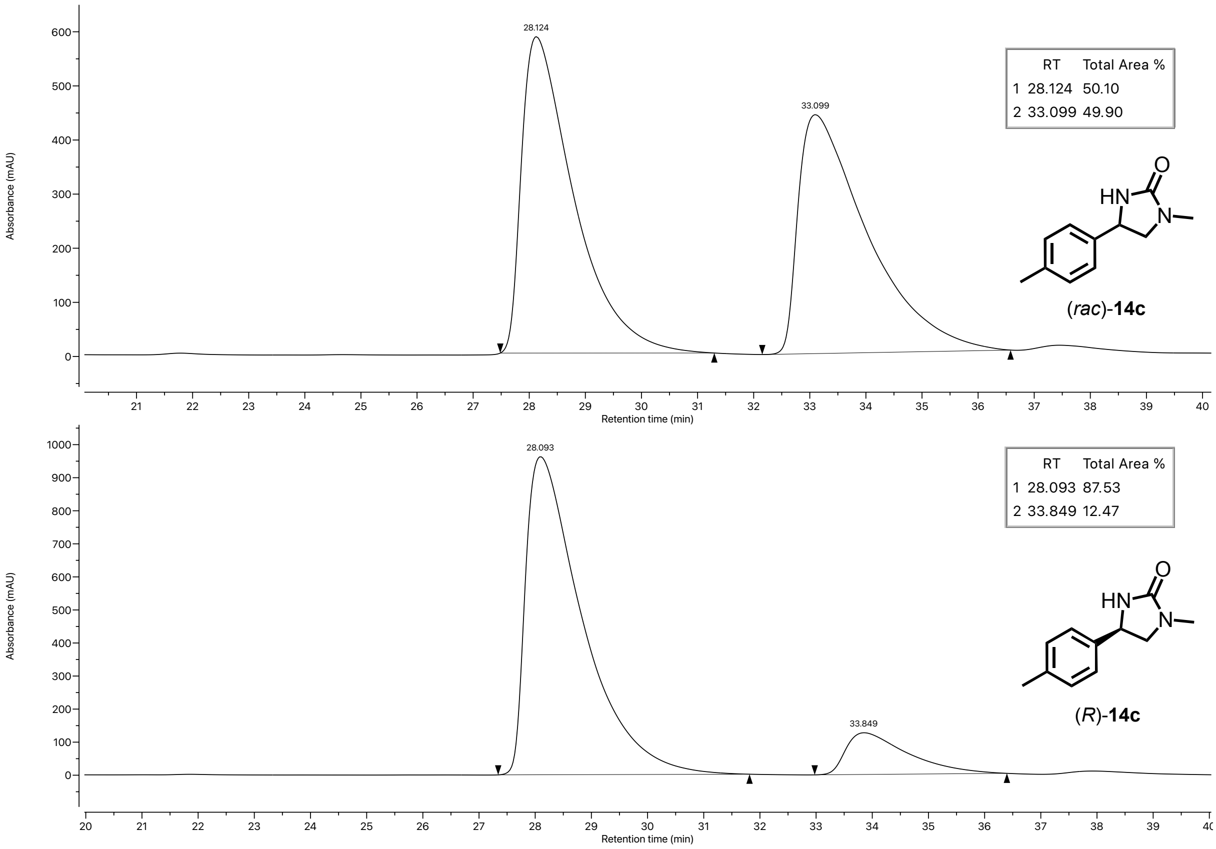

Figure S59: HPLC chromatogram of (*R*)-**14c** with 74% ee.

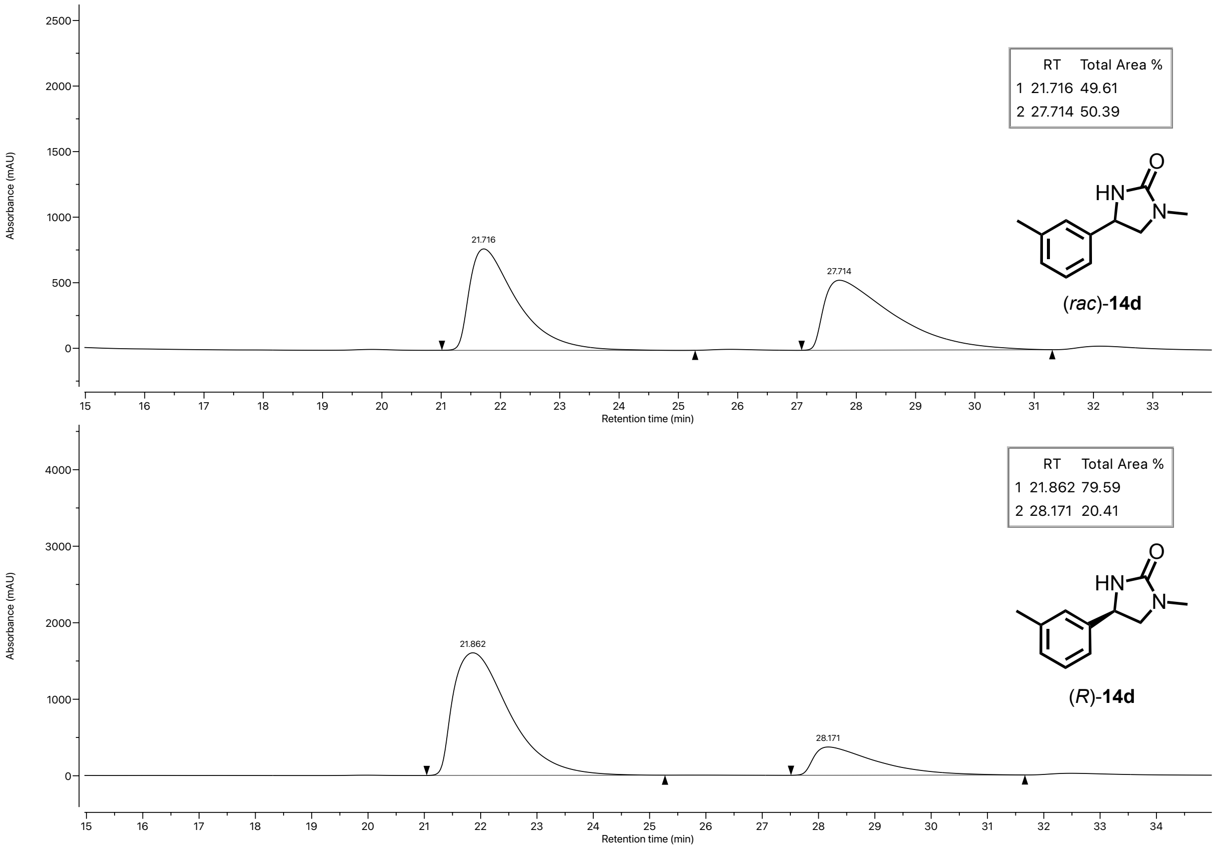

Figure S60: HPLC chromatogram of (*R*)-**14d** with 59% ee.

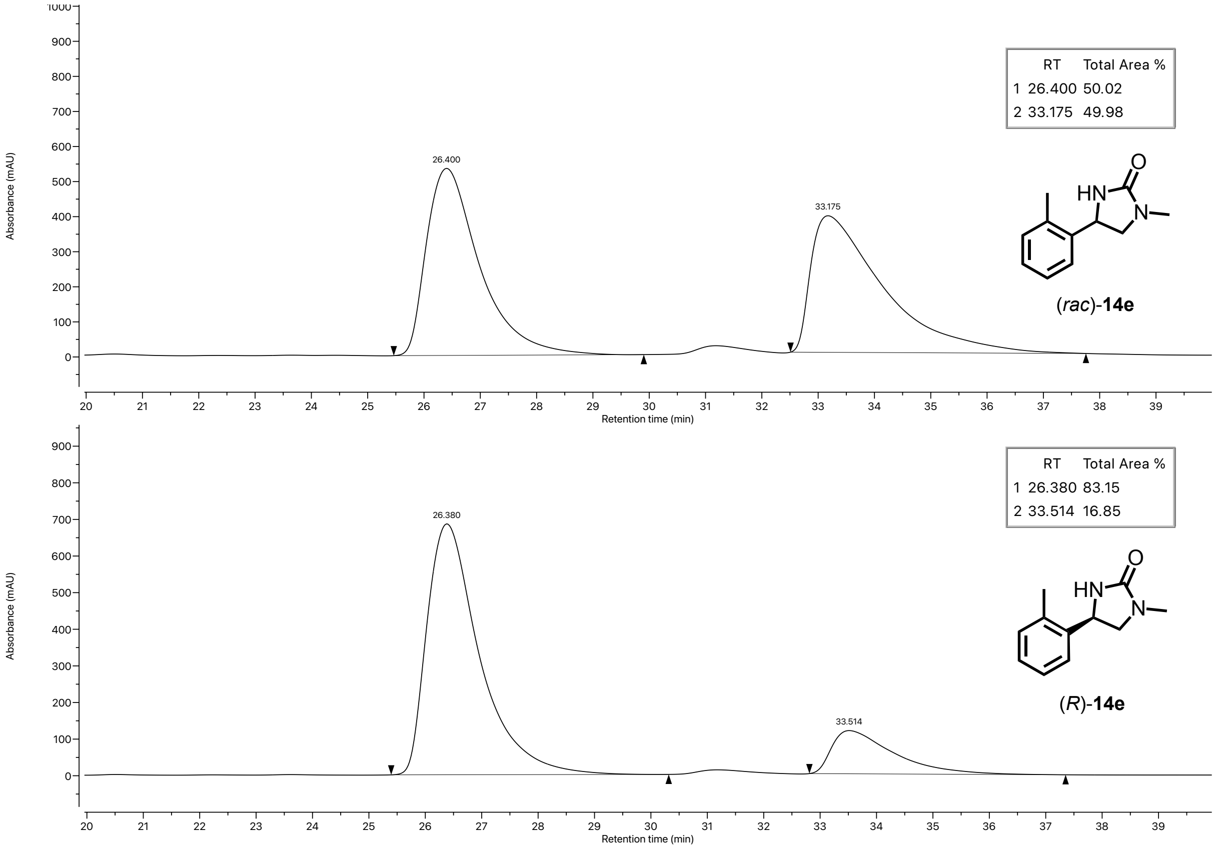

Figure S61: HPLC chromatogram of (*R*)-**14e** with 66% ee.

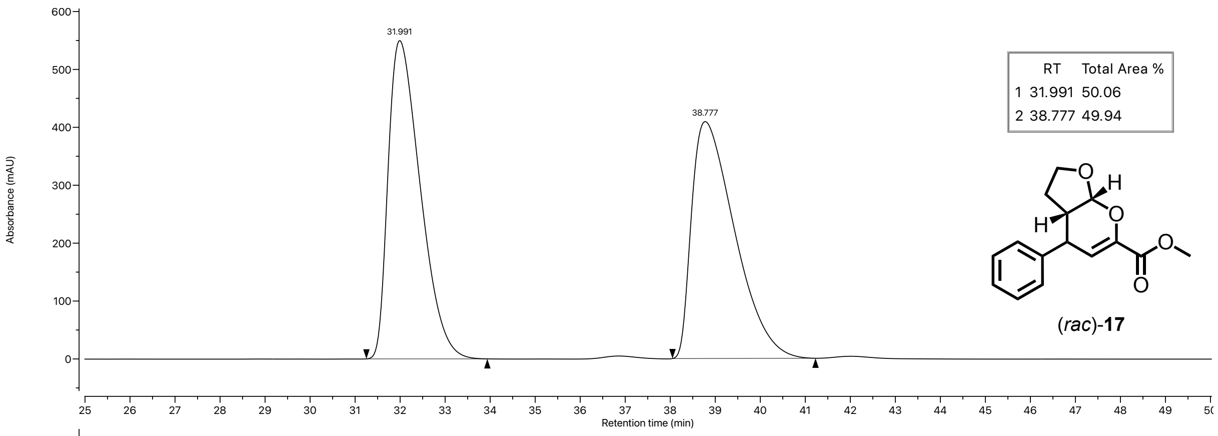

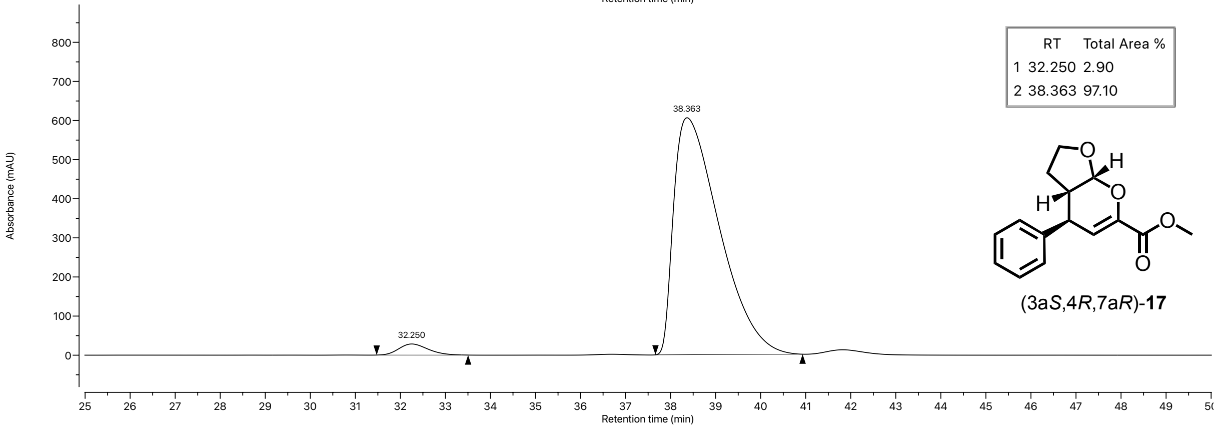

Figure S62: HPLC chromatogram of (3a*S*,4*R*,7a*R*)-**17** with 94% ee.

11. CD-Spectra

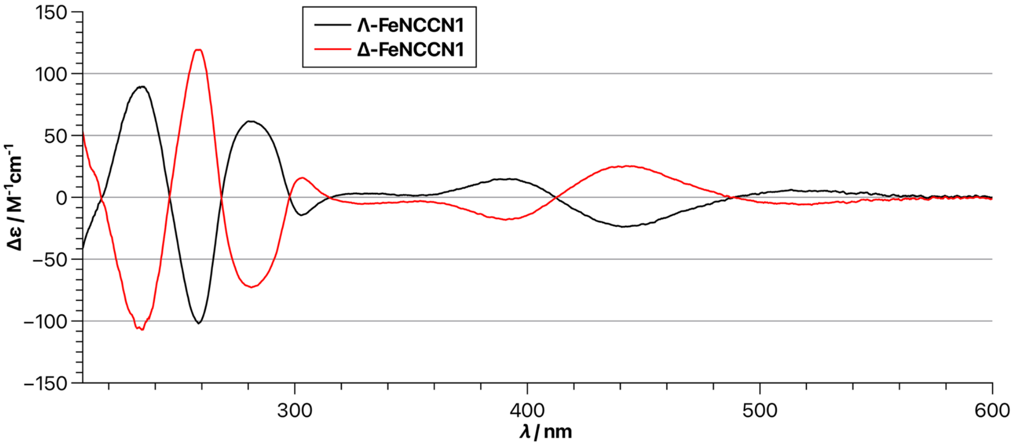

Figure S63: CD-spectra of Λ-**FeNCCN1** and Δ-**FeNCCN1** in MeCN (0.25 mM).

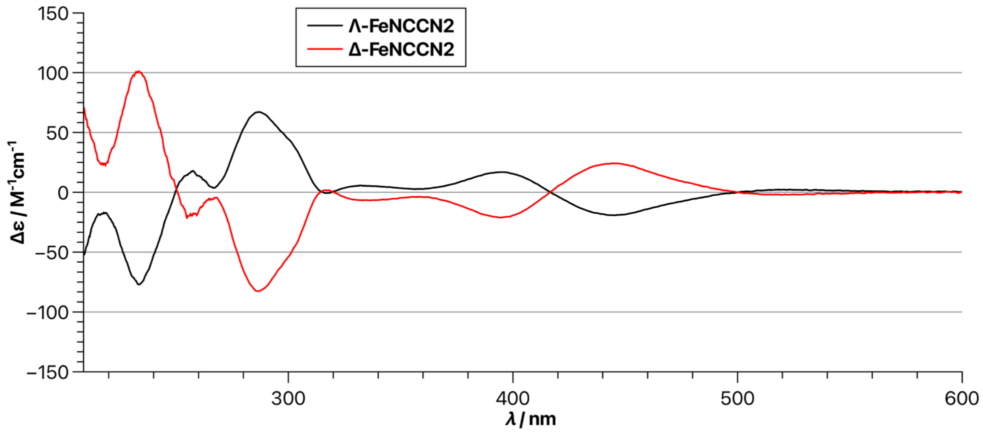

Figure S64 CD-spectra of Λ-**FeNCCN2** and Δ-**FeNCCN2** in MeCN (0.25 mM).

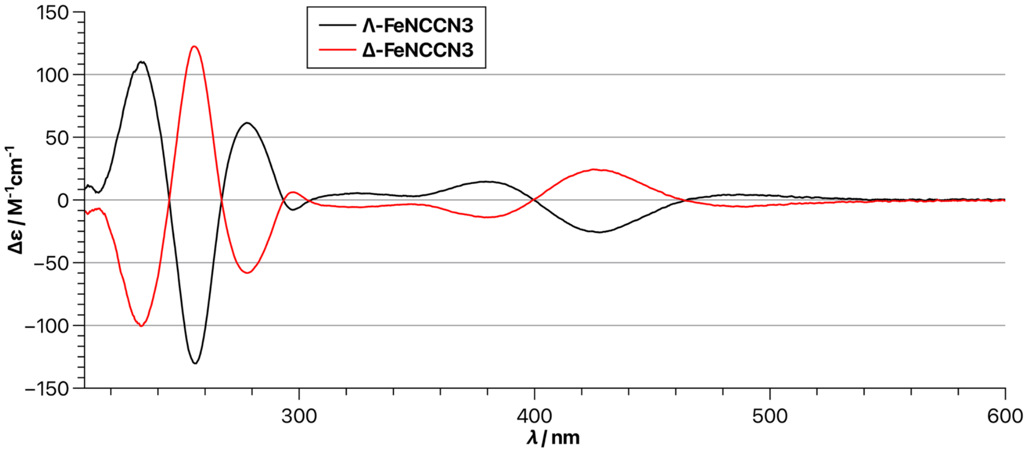

Figure S65: CD-spectra of Λ-**FeNCCN3** and Δ-**FeNCCN3** in MeCN (0.25 mM).

12. Single Crystal X-Ray Diffraction

rac-**FeNCCN1**

A suitable crystal of $C_{46}H_{32}F_6FeN_8(PF_6)_2 \cdot C_4H_{10}O \cdot C_2H_3N$ was selected under inert oil and mounted using a MiTeGen loop. Intensity data of the crystal were recorded with a STADIVARI diffractometer (Stoe & Cie). The diffractometer was operated with Cu-Kα radiation (1.54186 Å, microfocus source) and equipped with a Dectris PILATUS 300K detector. Evaluation, integration and reduction of the diffraction data was carried out using the X-Area software suite.[16] Multi-scan and numerical absorption corrections were applied with the LANA and X-RED32 modules of the X-Area software suite.[17,18] The structure was solved using dual-space methods (SHELXT-2018/2) and refined against F^2 (SHELXL-2019/1 using ShelXle interface).[19-21] All non-hydrogen atoms were refined with anisotropic displacement parameters. The hydrogen atoms were refined using the “riding model” approach with isotropic displacement parameters 1.2 times (1.5 times for the methyl groups) of that of the preceding carbon atom. Two out of three PF_6^- anions were refined disordered. The residual diffuse electron density belonging to disordered solvent molecules was eliminated using the SQUEEZE algorithm in the PLATON software.[22,23] CCDC 2543340 contains the supplementary crystallographic data for this paper. These data can be obtained free of charge from The Cambridge Crystallographic Data Centre via www.ccdc.cam.ac.uk/structures.

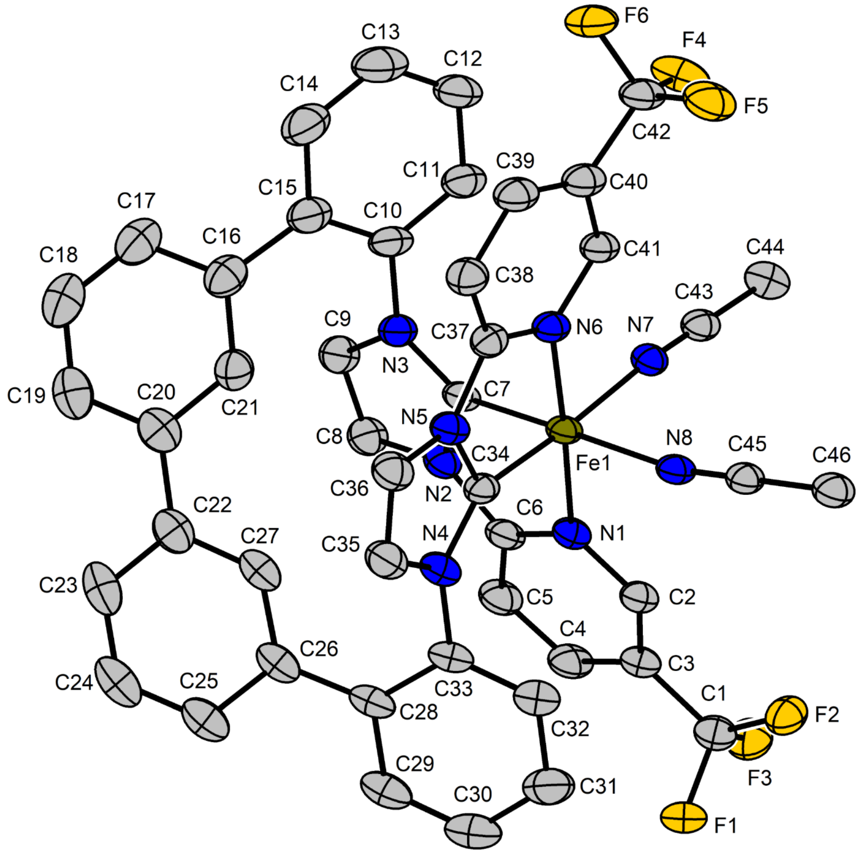

Figure S66: Crystal structure of the *rac*-**FeNCCN1** complex. The $[PF_6]^-$anions, hydrogen atoms, and solvent molecules were omitted for clarity. The displacement ellipsoids are shown at a probability level of 50% at 100 K.

Table S1: Selected crystallographic data and details of the structure determination for $C_{46}H_{32}F_6FeN_8(PF_6)_2 \cdot C_4H_{10}O \cdot C_2H_3N$

Identification code	LHM017
Empirical formula	$C_{46}H_{32}F_{18}FeN_8P_2$
Molar mass / g·mol^{-1}	1271.76
Space group (No.)	$P\bar{1}$ (2)
a / Å	11.9443(5)
b / Å	13.3381(4)
c / Å	20.0297(8)
α / °	97.029(3)
β / °	94.144(3)
γ / °	107.841(3)
V / Å^3	2994.3(2)
Z	2
$\rho_{calc.}$ / g·cm^{-3}	1.411
μ / mm^{-1}	3.426
Color	orange
Crystal habitus	plate
Crystal size / mm^3	0.144 x 0.077 x 0.028
T / K	100
λ / Å	1.54186 (Cu-K$_\alpha$)
ϑ range / °	4.732 to 75.500
Range of Miller indices	$-14 \le h \le 9$
	$-16 \le k \le 16$
	$-24 \le l \le 25$
Absorption correction	multi-scan and numerical
T_{min}, T_{max}	0.6164, 0.9102
R_{int}, R_σ	0.1021, 0.0652
Completeness of the data set	0.989
No. of measured reflections	65430
No. of independent reflections	12097
No. of parameters	820
No. of restraints	484
S (all data)	1.073
$R(F)$ ($I \ge 2\sigma(I)$, all data)	0.0651, 0.1074
$wR(F^2)$ ($I \ge 2\sigma(I)$, all data)	0.1554, 0.1795
Extinction coefficient	not refined
$\Delta\rho_{\text{max}}$, $\Delta\rho_{\text{min}}$ / e·Å^{-3}	0.771, –0.476

* Reflection contributions from highly disordered solvent were accounted for using the SQUEEZE algorithm in the PLATON software (Spek, 2015). The solvent accessible void contents were determined to be 93 e$^-$ per 484 Å^3. Because the exact identity and amount of solvent could not be established, no solvent was included in chemical formula entries. As a result, all calculated quantities that derive from the formula (e.g. density, molecular mass, etc.) are known to be inaccurate.

rac-**FeNCCN3**

A suitable crystal of $2C_{44}H_{34}FeN_8(PF_6)_2 \cdot C_4H_{10}O \cdot 2C_2H_3N$ was selected under inert oil and mounted using a MiTeGen loop. Intensity data of the crystal were recorded with a STADIVARI diffractometer (Stoe & Cie). The diffractometer was operated with Cu-Kα radiation (1.54186 Å, microfocus source) and equipped with a Dectris PILATUS 300K detector. Evaluation, integration and reduction of the diffraction data was carried out using the X-Area software suite.[16] Multi-scan and numerical absorption corrections were applied with the LANA and X-RED32 modules of the X-Area software suite.[17,18] The structure was solved using dual-space methods (SHELXT-2018/2) and refined against F^2 (SHELXL-2019/1 using ShelXle interface).[19-21] All non-hydrogen atoms were refined with anisotropic displacement parameters. The hydrogen atoms were refined using the "riding model" approach with isotropic displacement parameters 1.2 times (1.5 times for the methyl groups) of that of the preceding carbon atom. Two PF_6^- anions were refined disordered using the DSR plugin implemented in ShelXle.[24] CCDC 2543343 contains the supplementary crystallographic data for this paper. These data can be obtained free of charge from The Cambridge Crystallographic Data Centre via www.ccdc.cam.ac.uk/structures.

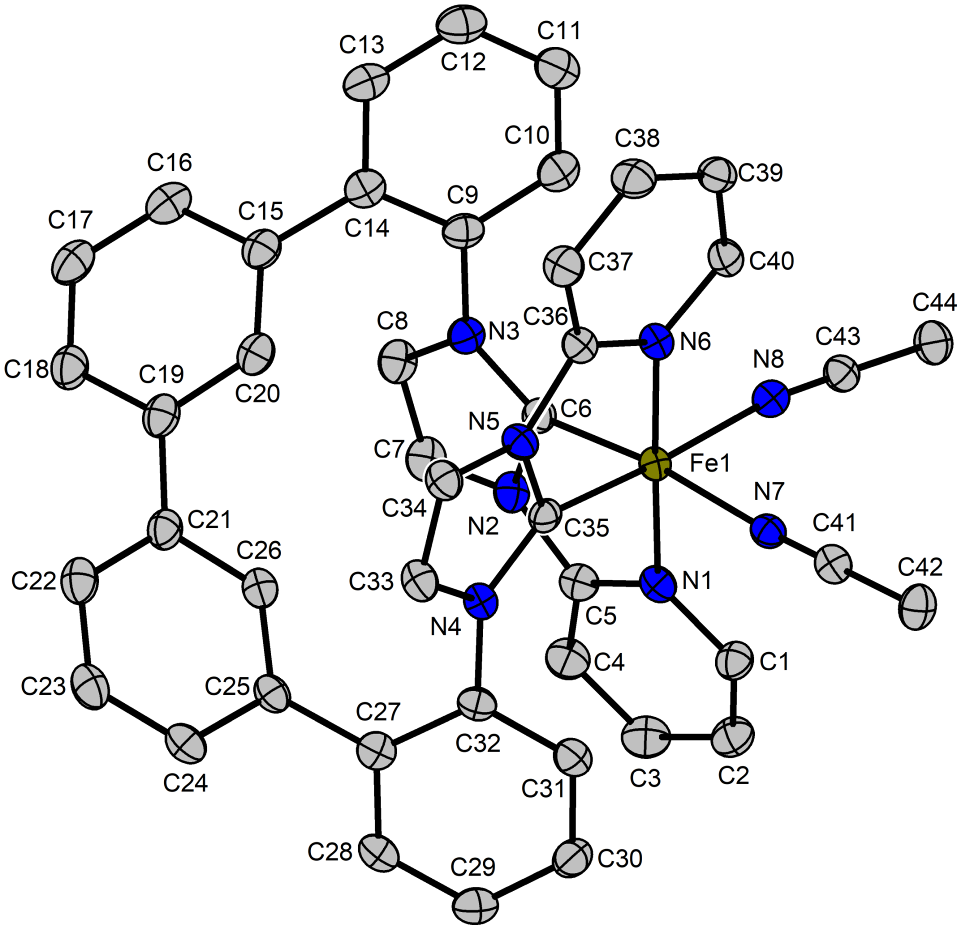

Figure S67: Crystal structure of the *rac*-**FeNCCN3** complex. The $[PF_6]^-$anions, hydrogen atoms, and solvent molecules were omitted for clarity. The displacement ellipsoids are shown at a probability level of 50% at 100 K.

Table S2: Selected crystallographic data and details of the structure determination for $2C_{44}H_{34}FeN_8(PF_6)_2 \cdot C_4H_{10}O \cdot 2C_2H_3N$

Identification code	LHP029
Empirical formula	$C_{96}H_{84}F_{24}Fe_2N_{18}OP_4$
Molar mass / g·mol^{-1}	2197.39
Space group (No.)	$P2_1/n$ (14)
a / Å	20.3825(3)
b / Å	11.31660(10)
c / Å	41.6685(6)
β / °	94.3410(10)
V / Å^3	9583.7(2)
Z	4
$\rho_{calc.}$ / g·cm^{-3}	1.523
μ / mm^{-1}	3.989
Color	orange
Crystal habitus	plate
Crystal size / mm^3	0.204 x 0.153 x 0.026
T / K	100
λ / Å	1.54186 (Cu-K$_\alpha$)
ϑ range / °	2.491 to 77.228
Range of Miller indices	$-24 \leq h \leq 25$
	$-7 \leq k \leq 13$
	$-51 \leq l \leq 52$
Absorption correction	multi-scan and numerical
$T_{\min}$, $T_{\max}$	0.4486, 0.9029
R_{int}, R_σ	0.0746, 0.0484
Completeness of the data set	0.995
No. of measured reflections	113854
No. of independent reflections	19858
No. of parameters	1442
No. of restraints	1360
S (all data)	0.955
$R(F)$ ($I \geq 2\sigma(I)$, all data)	0.0500, 0.0741
$wR(F^2)$ ($I \geq 2\sigma(I)$, all data)	0.1282, 0.1406
Extinction coefficient	not refined
$\Delta\rho_{\max}$, $\Delta\rho_{\min}$ / e·Å^{-3}	0.741, –0.857
Identification code	LHP029
Empirical formula	$C_{96}H_{84}F_{24}Fe_2N_{18}OP_4$

Δ-**FeAux1**

A suitable crystal of $C_{57}H_{37}F_7FeN_7O_2(PF_6) \cdot 2\ CH_2Cl_2$ was selected under inert oil and mounted using a MiTeGen loop. Intensity data of the crystal were recorded with a D8 Quest diffractometer (Bruker AXS). The instrument was operated with Mo-Kα radiation (0.71073 Å, microfocus source) and equipped with a PHOTON III C14 detector. Evaluation, integration and reduction of the diffraction data was carried out using the Bruker APEX 5 software suite.[25] Multi-scan and numerical absorption corrections were applied using the SADABS program.[26,27] The structure was solved using dual-space methods (SHELXT-2018/2) and refined against F^2 (SHELXL-2019/1 using ShelXle interface).[19-21] All non-hydrogen atoms were refined with anisotropic displacement parameters. The hydrogen atoms were refined using the "riding model" approach with isotropic displacement parameters 1.2 times (1.5 times for terminal methyl groups) of that of the preceding carbon atom. The $[PF_6]^-$ anion was refined disordered using the DSR plugin implemented in ShelXle.[24] CCDC 2543341 contains the supplementary crystallographic data for this paper. These data can be obtained free of charge from The Cambridge Crystallographic Data Centre via www.ccdc.cam.ac.uk/structures.

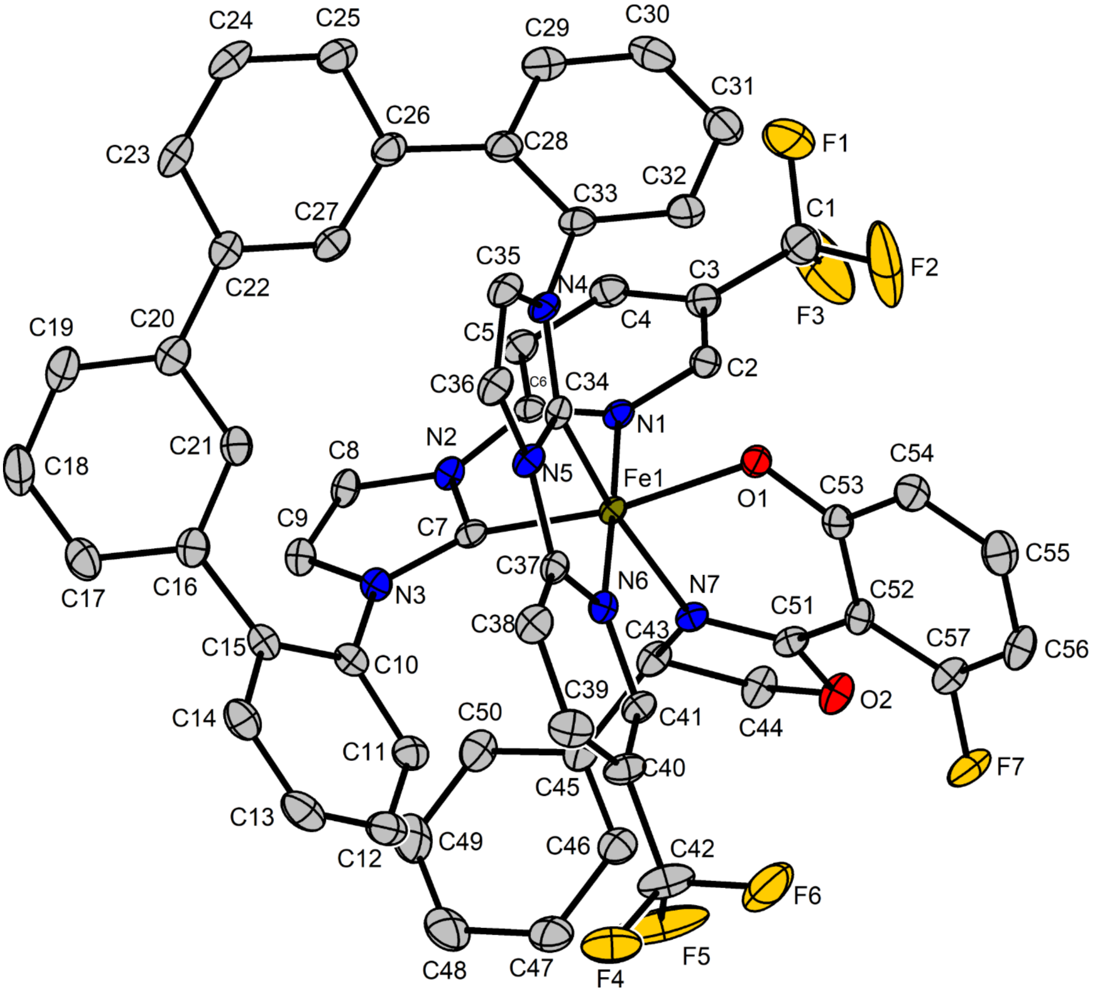

Figure S68: Crystal structure of the Δ-**FeAux1** complex. The $[PF_6]^-$anions, hydrogen atoms, and solvent molecules were omitted for clarity. The displacement ellipsoids are shown at a probability level of 50% at 100 K.

Table S3: Selected crystallographic data and details of the structure determination for $C_{57}H_{37}F_7FeN_7O_2(PF_6) \cdot 2\ CH_2Cl_2$.

Identification code	LHM065
Empirical formula	$C_{59}H_{41}Cl_4F_{13}FeN_7O_2P$
Molar mass / g·mol^{-1}	1355.61
Space group (No.)	$P1$ (1)
a / Å	11.1606(3)
b / Å	11.6916(4)
c / Å	12.4057(4)
α / °	92.2540(10)
β / °	109.5100(10)
γ / °	107.6130(10)
V / Å^3	1436.42(8)
Z	1
$\rho_{calc.}$ / g·cm^{-3}	1.567
μ / mm^{-1}	0.570
Color	red
Crystal habitus	plate
Crystal size / mm^3	0.194 x 0.142 x 0.032
T / K	100
λ / Å	0.71073 (Mo-K$_\alpha$)
ϑ range / °	1.850 to 28.426
Range of Miller indices	$-14 \leq h \leq 14$
	$-15 \leq k \leq 15$
	$-16 \leq l \leq 16$
Absorption correction	multi-scan and numerical
T_{min}, T_{max}	0.9397, 0.9873
R_{int}, R_σ	0.0500, 0.0342
Completeness of the data set	1.000
No. of measured reflections	90365
No. of independent reflections	14369
No. of parameters	848
No. of restraints	439
S (all data)	1.053
$R(F)$ ($I \geq 2\sigma(I)$, all data)	0.0405, 0.0446
$wR(F^2)$ ($I \geq 2\sigma(I)$, all data)	0.0945, 0.0966
Extinction coefficient	not refined
Flack parameter x	0.010(4)
$\Delta\rho_{max}$, $\Delta\rho_{min}$ / e·Å^{-3}	0.639, –0.675

Λ-**FeAux3**

A suitable crystal of $C_{55}H_{39}FFeN_7O_2(PF_6) \cdot 2CH_2Cl_2$ was selected under inert oil and mounted using a MiTeGen loop. Intensity data of the crystal were recorded with a D8 Venture diffractometer (Bruker AXS). The instrument was operated with Mo-Kα radiation (0.71073 Å, microfocus source) and equipped with a PHOTON III C14 detector. Evaluation, integration and reduction of the diffraction data was carried out using the Bruker APEX 5 software suite.[25] Multi-scan and numerical absorption corrections were applied using the SADABS program.[26,27] The structure was solved using dual-space methods (SHELXT-2018/2) and refined against F^2 (SHELXL-2019/1 using ShelXle interface).[19-21] All non-hydrogen atoms were refined with anisotropic displacement parameters. The hydrogen atoms were refined using the "riding model" approach with isotropic displacement parameters 1.2 times (1.5 times for terminal methyl groups) of that of the preceding carbon atom. The $[PF_6]^-$ anion was refined disordered using the DSR plugin implemented in ShelXle.[24] The residual electron density corresponding to disordered solvent was eliminated using the SQUEEZE algorithm in the PLATON software.[23,24] CCDC 2543342 contains the supplementary crystallographic data for this paper. These data can be obtained free of charge from The Cambridge Crystallographic Data Centre via www.ccdc.cam.ac.uk/structures.

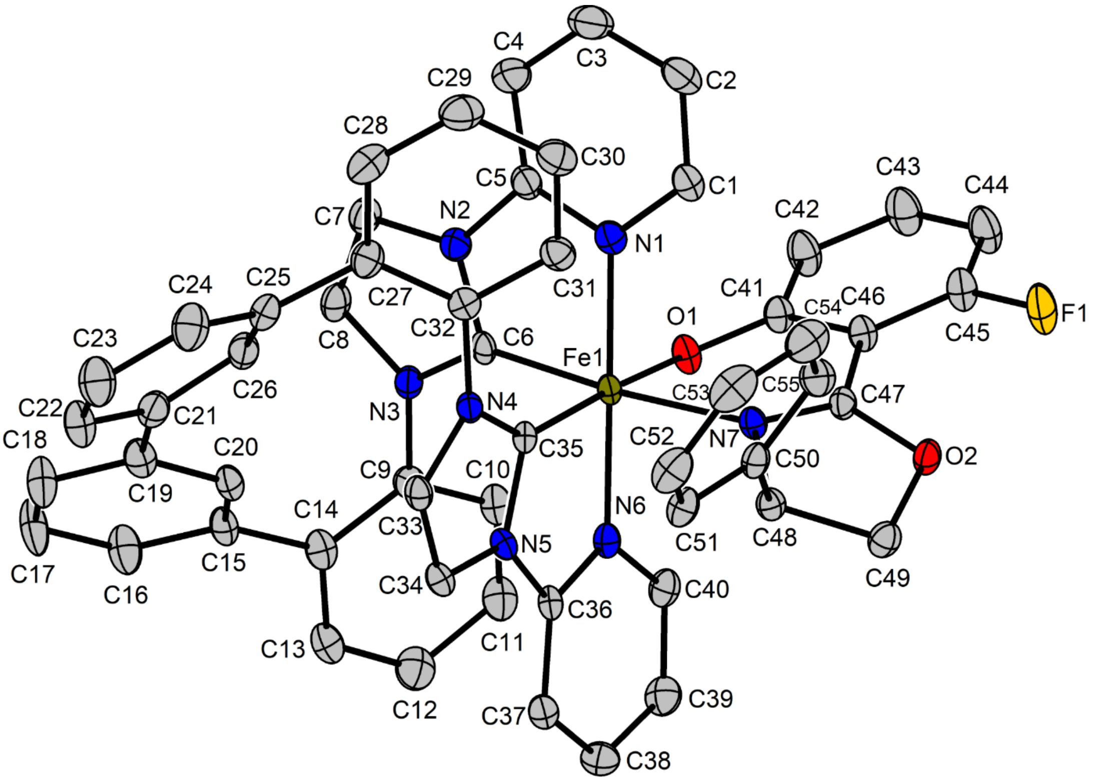

Figure S69: Crystal structure of the Λ-**FeAux3** complex. The $[PF_6]^-$anions, hydrogen atoms, and solvent molecules were omitted for clarity. The displacement ellipsoids are shown at a probability level of 50% at 100 K.

Table S4: Selected crystallographic data and details of the structure determination for $C_{55}H_{39}FFeN_7O_2(PF_6) \cdot 2CH_2Cl_2$.

Identification code	LHP028F1
Empirical formula	$C_{57}H_{43}Cl_4F_7FeN_7O_2P$
Molar mass / g·mol^{-1}	1219.60
Space group (No.)	$P2_12_12_1$ (19)
a / Å	12.6909(4)
b / Å	16.7271(5)
c / Å	28.5491(7)
V / Å^3	6060.5(3)
Z	4
$\rho_{calc.}$ / g·cm^{-3}	1.337
μ / mm^{-1}	0.519
Color	red
Crystal habitus	block
Crystal size / mm^3	0.924 x 0.420 x 0.386
T / K	100
λ / Å	0.71073 (Mo-K$_\alpha$)
ϑ range / °	2.014 to 28.334
Range of Miller indices	$-16 \le h \le 16$
	$-22 \le k \le 22$
	$-38 \le l \le 36$
Absorption correction	multi-scan and numerical
T_{min}, T_{max}	0.7551, 0.9141
R_{int}, R_σ	0.0395, 0.0194
Completeness of the data set	0.999
No. of measured reflections	181879
No. of independent reflections	15091
No. of parameters	776
No. of restraints	442
S (all data)	1.047
$R(F)$ ($I \ge 2\sigma(I)$, all data)	0.0418, 0.0443
$wR(F^2)$ ($I \ge 2\sigma(I)$, all data)	0.1035, 0.1050
Extinction coefficient	not refined
Flack parameter x	0.020(3)
$\Delta\rho_{max}$, $\Delta\rho_{min}$ / e·Å^{-3}	1.264, –0.795

* Reflection contributions from highly disordered solvent were accounted for using the SQUEEZE algorithm in the PLATON software (Spek, 2015). The solvent accessible void contents were determined to be 170 e$^-$ per 570 Å^3 for the first void and 170 e$^-$ per 569 Å^3 for the second void. Because the exact identity and amount of solvent could not be established, no solvent was included in chemical formula entries. As a result, all calculated quantities that derive from the formula (e.g. density, molecular mass, etc.) are known to be inaccurate.

Δ-**FeAux3**

A suitable crystal of $C_{55}H_{39}FFeN_7O_2(PF_6) \cdot 3CH_2Cl_2$ was selected under inert oil and mounted using a MiTeGen loop. Intensity data of the crystal were recorded with a D8 Quest diffractometer (Bruker AXS). The instrument was operated with Mo-Kα radiation (0.71073 Å, microfocus source) and equipped with a PHOTON III C14 detector. Evaluation, integration and reduction of the diffraction data was carried out using the Bruker APEX 5 software suite.[25] Multi-scan and numerical absorption corrections were applied using the SADABS program.[26,27] The structure was solved using dual-space methods (SHELXT-2018/2) and refined against F^2 (SHELXL-2019/1 using ShelXle interface).[19-21] All non-hydrogen atoms were refined with anisotropic displacement parameters. The hydrogen atoms were refined using the "riding model" approach with isotropic displacement parameters 1.2 times (1.5 times for terminal methyl groups) of that of the preceding carbon atom. One DCM solvent molecule was refined disordered using the DSR plugin implemented in ShelXle.[24] CCDC 2543344 contains the supplementary crystallographic data for this paper. These data can be obtained free of charge from The Cambridge Crystallographic Data Centre via www.ccdc.cam.ac.uk/structures.

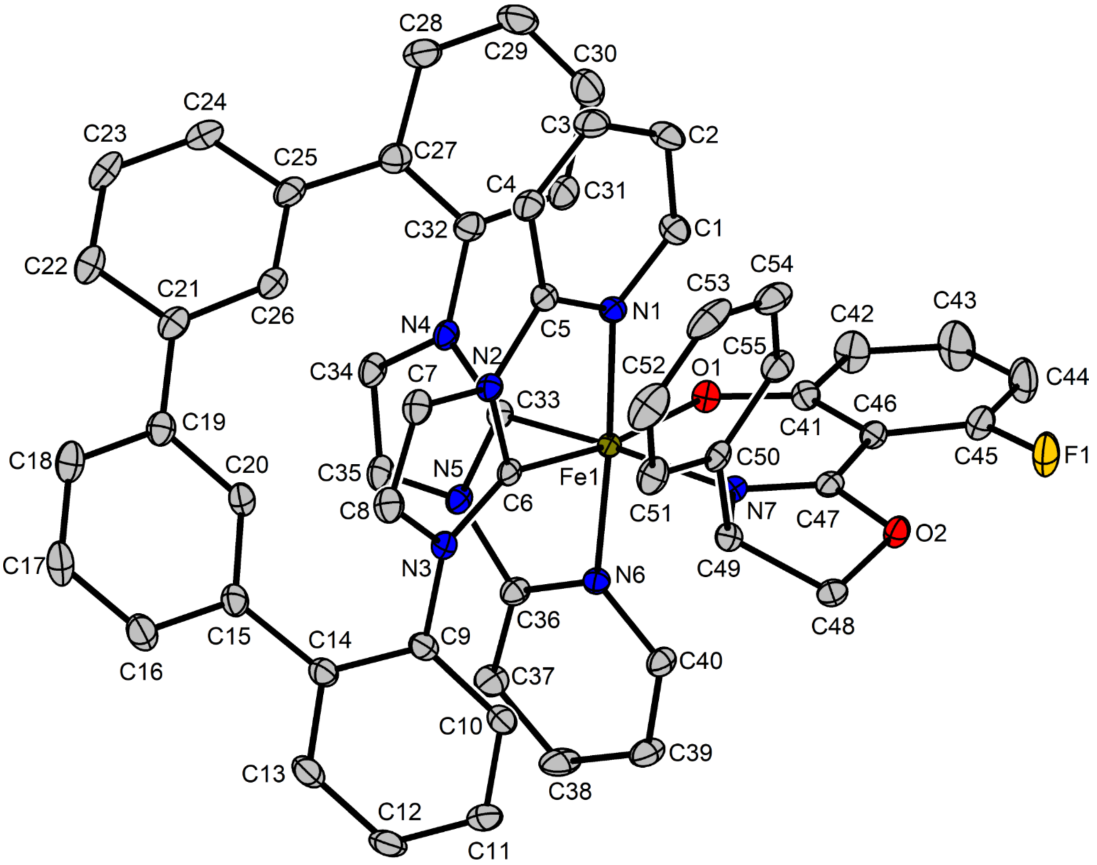

Figure S70: Crystal structure of the Δ-**FeAux3** complex. The $[PF_6]^-$anions, hydrogen atoms, and solvent molecules were omitted for clarity. The displacement ellipsoids are shown at a probability level of 50% at 100 K.

Table S5: Selected crystallographic data and details of the structure determination for $C_{55}H_{39}FFeN_7O_2(PF_6) \cdot 3CH_2Cl_2$.

Identification code	LHP071F2
Empirical formula	$C_{58}H_{45}Cl_6F_7FeN_7O_2P$
Molar mass / g·mol^{-1}	1304.53
Space group (No.)	$P2_12_12_1$ (19)
a / Å	12.9806(6)
b / Å	14.0833(5)
c / Å	30.4194(12)
V / Å^3	5561.0(4)
Z	4
$\rho_{calc.}$ / g·cm^{-3}	1.558
μ / mm^{-1}	0.664
Color	red
Crystal habitus	needle
Crystal size / mm^3	0.780 x 0.190 x 0.138
T / K	100
λ / Å	0.71073 (Mo-K$_\alpha$)
ϑ range / °	1.971 to 28.292
Range of Miller indices	$-17 \leq h \leq 17$
	$-18 \leq k \leq 18$
	$-40 \leq l \leq 40$
Absorption correction	multi-scan and numerical
T_{min}, T_{max}	0.7529, 0.9970
R_{int}, R_σ	0.0366, 0.0191
Completeness of the data set	0.999
No. of measured reflections	137807
No. of independent reflections	13828
No. of parameters	767
No. of restraints	65
S (all data)	1.105
$R(F)$ ($I \geq 2\sigma(I)$, all data)	0.0307, 0.0317
$wR(F^2)$ ($I \geq 2\sigma(I)$, all data)	0.0721, 0.0727
Extinction coefficient	not refined
Flack parameter x	0.009(3)
$\Delta\rho_{max}$, $\Delta\rho_{min}$ / e·Å^{-3}	0.734, –0.643

13. References

[S1] Cui, T.; Ye, C.-X.; Thelemann, J.; Jenisch, D.; Meggers, E. Enantioselective and Enantioconvergent Iron-Catalyzed C(sp^3)-H Aminations to Chiral 2-Imidazolidinones. *Chin. J. Chem.* **2023**, *41*, 2065–2070.

[S2] Gremaud, L.; Alexakis, A. Enantioselective Copper-Catalyzed Conjugate Addition of Trimethylaluminium to β,γ-Unsaturated α-Ketoesters. *Angew. Chem. Int. Ed*. **2012**, *51*, 794-797.

[S3] Zhao, Y.; Gilbertson, S. R. Synthesis of Proline-Based N-Heterocyclic Carbene Ligands. *Org. Lett.* **2014**, *16*, 1033.

[S4] Matsumura, M.; Kitamura, Y.; Yamauchi, A.; Kanazawa, Y.; Murata, Y.; Hyodo, T.; Yamaguchi, K.; Yasuike, S. Synthesis of benzo[*d*]imidazo[2,1-*b*]benzoselenoazoles: Cs_2CO_3-mediated cyclization of 1-(2-bromoaryl)benzimidazoles with selenium. *Beilstein J. Org. Chem.* **2019**, *15*, 2029.

[S5] Nandi, R.; Niyogi, S.; Kundu, S.; Mondal, A.; Roy, N. K.; Bisai, A. Highly Regioselective Oxidative Csp^2–H Amination for Indolosesquiterpene Alkaloids: Total Synthesis of (+)-dioridamycin. *Org. Chem. Front.* **2025**, *12*, 928.

[S6] Zhou, Z.; Chen, S.; Qin, J.; Nie, X.; Harms, K.; Meggers, E. Catalytic Enantioselective Intramolecular C(sp^3)–H Amination of 2-Azidoacetamides. *Angew. Chem. Int. Ed.* **2019**, *58*, 1088.

[S7] Demirel, N.; Haber, J.; Ivlev, S. I.; Meggers, E. Improving the Configurational Stability of Chiral-at-Iron Catalysts Containing Two *N*-(2Pyridyl)-Substituted *N*-Heterocyclic Carbene Ligands. *Organometallics* **2022**, *41*, 3852–3860.

[S8] Hong, Y.; Jarrige, L.; Harms, K.; Meggers, E. Chiral-at-Iron Catalyst: Expanding the Chemical Space for Asymmetric Earth-Abundant Metal Catalysis. *J. Am. Chem. Soc.* **2019**, *141*, 4569–4572.

[S9] Demirel, N.; Moths, P.; Xie, X.; Ivlev, S. I.; Meggers, E. Development of Chiral-at-Ruthenium Mesoionic Carbene Catalysts. *Chem. Eur. J.* **2025**, *31*, e202403792.

[S10] Demirel, N.; Dawor, M.; Nadler, G.; Ivlev, S. I.; Meggers, E. Stereogenic-at-iron Mesoionic Carbene Complex for Enantioselective C-H-Amidation. *Chem. Sci.* **2024**, *15*, 15625–15631.

[S11] Zhou, Z.; Tan, Y.; Yamahira, T.; Ivlev, S.; Xie, X.; Riedel, R.; Hemming, M.; Kimura, M.; Meggers, E. Enantioselective Ring-Closing C–H Amination of Urea Derivatives. *Chem* **2020**, *6*, 2024–2034.

[S12] Hong, Y.; Cui, T.; Ivlev, S.; Xie, X.; Meggers, E. Chiral-at-Iron Catalyst for Highly Enantioselective and Diastereoselective Hetero-Diels-Alder Reaction. *Chem. Eur. J.* **2021**, *27*, 8557–8563.

[S13] Franco, D.; Gómez, M.; Jiménez, F.; Muller, G.; Rocamora, M.; Maestro, M. A.; Mahía, J. Exo- and Endocyclic Oxazolinyl–Phosphane Palladium Complexes: Catalytic Behavior in Allylic Alkylation Processes. *Organometallics* **2004**, *23*, 3197.

[S14] Ma, J.; Zhang, X.; Huang, X.; Luo, S.; Meggers, E. Preparation of chiral-at-metal catalysts and their use in asymmetric photoredox chemistry. *Nat. Protoc.* **2018**, *13*, 605–632.

[S15] Hallen, L.; Horan, A. M.; Twamley, B.; McGarrigle, E. M.; Draper, S. M. Accessing unsymmetrical Ru(II) bipyridine complexes: a versatile synthetic mechanism for fine tuning photophysical properties. *Chem. Commun*. **2023**, *59*, 330–333.

[S16] *X-Area 1.8.1*, STOE & Cie GmbH, Darmstadt, Germany, **2018**.

[S17] *LANA - Laue Analyzer V 1.76.6*, STOE & Cie GmbH, Darmstadt, Germany, **2019**.

[S18] *X-RED32 V 1.65*, STOE & Cie GmbH, Darmstadt, Germany, **2018**.

[S19] Sheldrick, G. M. SHELXT – Integrated space-group and crystal-structure determination. *Acta Crystallogr., Sect. A: Found. Adv*. **2015**, *71*, 3-8.

[S20] Sheldrick, G. M. Crystal structure refinement with SHELXL. *Acta Crystallogr., Sect. C: Struct. Chem*. **2015**, *71*, 3-8.

[S21] Hübschle, C.B.; Sheldrick, G. M.; Dittrich, B. ShelXle: a Qt graphical user interface for SHELXL. *J. Appl. Crystallogr*. **2011**, *44*, 1281-1284.

[S22] Spek, A. L. PLATON SQUEEZE: a tool for the calculation of the disordered solvent contribution to the calculated structure factors. *Acta Crystallogr., Sect. C: Struct. Chem*. **2015**, *71*, 9–18.

[S23] Spek, A. L. PLATON - A Multipurpose Crystallographic Tool, Utrecht University, Utrecht, The Netherlands, **2019**.

[S24] Kratzert, D.; Krossing, I. Recent improvements in DSR. *J. Appl. Crystallogr*. **2018**, *51*, 928–934.

[S25] *APEX5*, Bruker AXS Inc., Madison, Wisconsin, USA, **2023**.

[S26] *SADABS*, Bruker AXS Inc., Madison, Wisconsin, USA, **2016**.

[S27] Krause, L.; Herbst-Irmer, R.; Sheldrick, G. M.; Stalke, D. Comparison of silver and molybdenum microfocus X-ray sources for single-crystal structure determination. *J. Appl. Crystallogr*. **2015**, *48*, 3.
